# CRISPR/Cas9-based genome-wide screening of the deubiquitinase subfamily identifies USP3 as a protein stabilizer of REST blocking neuronal differentiation and promotes neuroblastoma tumorigenesis

**DOI:** 10.1186/s13046-023-02694-1

**Published:** 2023-05-12

**Authors:** Janardhan Keshav Karapurkar, Min-Seong Kim, Jencia Carminha Colaco, Bharathi Suresh, Neha Sarodaya, Dong-Ho Kim, Chang-Hwan Park, Seok-Ho Hong, Kye-Seong Kim, Suresh Ramakrishna

**Affiliations:** 1 Graduate School of Biomedical Science and Engineering, Hanyang University, Seoul, 04763 South Korea; 2 College of Medicine, Hanyang University, Seoul, 04763 South Korea; 3 Department of Internal Medicine, School of Medicine, Kangwon National University, Chuncheon, South Korea

**Keywords:** Neuronal tumors, Prognostic marker, Protein stabilization, Protein turn-over, RA-induced differentiation, Ubiquitination, Ubiquitin-specific protease

## Abstract

**Background:**

The repressor element-1 silencing transcription factor (REST), a master transcriptional repressor, is essential for maintenance, self-renewal, and differentiation in neuroblastoma. An elevated expression of REST is associated with impaired neuronal differentiation, which results in aggressive neuroblastoma formation. E3 ligases are known to regulate REST protein abundance through the 26 S proteasomal degradation pathway in neuroblastoma. However, deubiquitinating enzymes (DUBs), which counteract the function of E3 ligase-mediated REST protein degradation and their impact on neuroblastoma tumorigenesis have remained unexplored.

**Methods:**

We employed a CRISPR/Cas9 system to perform a genome-wide knockout of ubiquitin-specific proteases (USPs) and used western blot analysis to screen for DUBs that regulate REST protein abundance. The interaction between USP3 and REST was confirmed by immunoprecipitation and Duolink in situ proximity assays. The deubiquitinating effect of USP3 on REST protein degradation, half-life, and neuronal differentiation was validated by immunoprecipitation, in vitro deubiquitination, protein-turnover, and immunostaining assays. The correlation between USP3 and REST expression was assessed using patient neuroblastoma datasets. The USP3 gene knockout in neuroblastoma cells was performed using CRISPR/Cas9, and the clinical relevance of USP3 regulating REST-mediated neuroblastoma tumorigenesis was confirmed by in vitro and in vivo oncogenic experiments.

**Results:**

We identified a deubiquitinase USP3 that interacts with, stabilizes, and increases the half-life of REST protein by counteracting its ubiquitination in neuroblastoma. An *in silico* analysis showed a correlation between USP3 and REST in multiple neuroblastoma cell lines and identified USP3 as a prognostic marker for overall survival in neuroblastoma patients. Silencing of USP3 led to a decreased self-renewal capacity and promoted retinoic acid-induced differentiation in neuroblastoma. A loss of USP3 led to attenuation of REST-mediated neuroblastoma tumorigenesis in a mouse xenograft model.

**Conclusion:**

The findings of this study indicate that USP3 is a critical factor that blocks neuronal differentiation, which can lead to neuroblastoma. We envision that targeting USP3 in neuroblastoma tumors might provide an effective therapeutic differentiation strategy for improved survival rates of neuroblastoma patients.

**Supplementary Information:**

The online version contains supplementary material available at 10.1186/s13046-023-02694-1.

## Introduction

Neuroblastoma is the most common pediatric extracranial solid tumor. It arises from abnormal development of the neural crest lineage and is characterized by aberrant neuronal differentiation. Neuroblastoma originates as a heterogeneous group of tumors from neural crest lineage-derived sympathoadrenal precursor cells and predominantly consists of poorly differentiated neuroblasts [1, 2]. A variety of studies have demonstrated that the overall survival of neuroblastoma patients is highly dependent on the degree of differentiation of the neuroblastoma cells [1]. Retinoic acid (RA) has been successfully used in preclinical and clinical trials as an effective inducer of neuroblastoma differentiation post-chemotherapy. However, more than 50% of neuroblastoma patients achieve clinical remission and develop resistance to RA-treatment [2]. Therefore, investigating the molecular mechanism that enhances neuroblastoma differentiation is of great clinical significance.

Transcriptional regulation of the differentiation and maintenance of neural stem cells plays a critical role in a variety of neural cancers and neurodegenerative disorders [3]. The repressor element-1 (RE-1) silencing transcription factor (REST) is a master transcriptional repressor of the neuronal genes involved in self-renewal, neurogenesis, and neural differentiation [4–6]. REST binds to RE-1, a highly conserved 21–23 bp DNA sequence located in several neuronal genes, and causes silencing of the transcription of target genes by recruiting specific co-repressors [7]. During neural differentiation, REST expression decreases rapidly and is maintained at low levels in mature neurons [8, 9]. The aberrant expression of the REST protein has been associated with a variety of tumors and neurodegenerative disorders [10]. In neural tumors such as neuroblastoma, glioblastoma, and medulloblastoma, a high level of REST expression was correlated with the severity of a tumor [11]. High REST expression was also associated with the undifferentiated phenotype [12]. In contrast, interfering with REST protein levels abrogates tumor proliferation, invasion, and self-renewal both in vitro and in vivo [13], suggesting that the REST protein level is a critical factor during neuroblastoma formation.

Several reports suggest that REST undergo post-translational regulation by a ubiquitination system to maintain cellular proteostasis [9, 12, 14]. During differentiation of neural precursor cells, the REST protein level is decreased due to rapid degradation through the proteasomal degradation pathway by the E3 ubiquitin ligase complex SCF-β-TrCP [9, 14]. In contrast, the SCF-β-TrCP E3 ligase expression is increased in differentiated neuroblastoma tumors, indicating a negative correlation between the expression of REST and SCF-β-TrCP E3 ligase [9, 12]. Thus, SCF-β-TrCP E3 ligase-mediated REST protein degradation consequently rescues the neuronal differentiation blockade. However, the reversal of REST protein degradation by deubiquitinating enzymes (DUBs) may play an equally critical role in REST protein stabilization and therefore in neuroblastoma malignant transformation. Thus, elucidation of the deubiquitinating mechanism that controls REST protein turnover and its implications for self-renewal and tumorigenic competence in neuroblastoma would provide us with new insights for effective alternative therapeutics.

Ubiquitin-specific proteases (USPs) are the largest subfamily of DUBs and are involved in diverse cellular functions, such as cell signaling, DNA repair, cell-cycle regulation, and stem cell differentiation [15, 16]. DUBs catalyze proteolytic removal of ubiquitin moieties conjugated to their target substrate prior to protein degradation. Extensive research has suggested that DUBs could be potential therapeutic targets in the treatment of neuroblastoma [1, 17, 18]. USP14, HAUSP, and UCHL1 have been identified as key regulators of neuroblastoma differentiation and may play a critical role in neuroblastoma pathogenesis [1, 17, 19]. Despite considerable study on the expression level of several DUBs in neuroblastoma, DUBs regulating REST protein turnover and its impact on neuroblastoma tumorigenesis are yet to be explored.

In this study, we implemented our recently developed CRISPR-Cas9-based single-guide RNA (sgRNA) library to target genome-wide DUBs that alter REST protein levels in neuroblastoma. The loss-of-function-based screening for DUB genes identified USP3 as a regulator of REST protein levels in neuroblastoma. We demonstrate that USP3 is a bona fide DUB that interacts with and regulates REST protein turnover by rescuing its protein degradation through the ubiquitin-proteasomal pathway. Furthermore, we found that USP3 regulates self-renewal and promotes RA-induced differentiation in neuroblastoma tumors. In contrast, loss of USP3 reduced neuroblastoma cell proliferation, migration, and invasion in vitro, and suppressed tumor growth in vivo. Altogether, our results elucidate a novel mechanism of USP3-mediated regulation of REST protein levels in regulating neuroblastoma cell differentiation, self-renewal, and tumorigenesis.

## Materials and methods

### Plasmids, shRNAs, and sgRNAs

Flag-REST (#41,903), Flag-USP3 (#22,582), and HA-ubiquitin (#18,712) were purchased from Addgene (Watertown, MA, USA). Myc-tagged REST was obtained by subcloning REST into a pCDNA3-6XMyc-vector. The USP3 catalytic mutant was generated by replacing the active cysteine residue at position 168 with serine by site-directed mutagenesis on the Flag-USP3 construct to generate Flag-USP3 C168S (USP3CS). All the constructs were confirmed by Sanger sequencing. The shRNA targeting USP3 was purchased from Santa Cruz Biotechnology (sc-76,835-SH; Dallas, TX, USA).

To screen the DUBs that regulated the REST protein level, we purchased plasmids encoding Cas9-2 A-mRFP-2 A-PAC (puromycin N-acetyl-transferase, puromycin resistance gene) and single-guide RNAs (sgRNAs) vectors from Toolgen (Seoul, South Korea). As described previously [20], the sgRNA target sequences were designed using an online application (https://portals.broadinstitute.org/gppx/crispick/public) and cloned into the pRG2-CT sgRNA vector. Briefly, the sgRNA target sequences were synthesized by Bioneer (Seoul, South Korea) and the terminal phosphates was added using T4 polynucleotide kinase to the annealed oligonucleotides (Bio-Rad, CA, USA). The sgRNA vector was digested with *BsaI* restriction enzyme and ligated with the annealed oligonucleotide sequence. The target sgRNA oligonucleotide sequence is listed in Supplementary Table S1.

### Antibodies and reagents

Rabbit polyclonal antibodies were obtained against human REST (07-579, Merck at dilution 1:1000 for western blot, 1:500 for immunofluorescence, and 1:100 for immunohistochemistry), USP3 (Abcam ab101473, Genetex GTX128238) at a dilution of 1:1000 for western blots, USP3 (Genetex GTX128238) at a dilution of 1:500 for immunofluorescence, USP3 (Novus NB100-77284) 1:100 for immunohistochemistry, and Oct-4 (Abcam ab18976, 1:1000). Antibodies against Vimentin (V9) (sc-6260, 1:1000), GFAP (2E1) (sc-33,673, 1:1000) Phox2A (37 K-2) (sc-81,978, 1:500), Phox2b (sc-376,997, 1:500), Trk (B-3) (sc- 7268, 1:1000), PRX1 (1E2) (sc-293,386, 1:500), c-Myc (sc-40, 1:1000), ubiquitin (sc-8017, 1:1000), HA (sc-7392, 1:1000), β-actin (sc-47,778, 1:1000), USP3 (sc-135,597, 1:1000), GAPDH (sc-32,233, 1:1000), Sox-2 (sc-365,964, 1:1000), and normal mouse IgG (sc-2025, 1:1000) were purchased from Santa Cruz Biotechnology (Dallas, TX, USA). Mouse monoclonal antibodies were acquired against Flag Anti-DDDDK-tag (M185-3 L, 1:1000, MBL Life Science), NESTIN (MAB1259; 1:1000 for western blot and 1:500 for immunofluorescence, R&D System), TH (MAB318; 1:1000 for western blot), Tuj-1 (MAB1637, 1:1000 for western blot and 1:500 for immunofluorescence, Chemicon), MAP2 (Abcam, ab11267, 1:1000 for western blot) and DLK1 (3A10) (Genetex, GTX60511). The Alexa Fluor conjugated secondary antibodies, Alexa Fluor^™^ 488 (A21202) and Alexa Fluor^™^ 594 (A21207) were purchased from Invitrogen, Life Technologies. Protein A/G Plus agarose beads (sc-2003) were acquired from Santa Cruz Biotech. The protease inhibitor cocktail was purchased from Roche (Cat. no. 11,836,153,001). The immunoprecipitation (IP) lysis buffer was purchased from Thermo Fisher (Cat. no. 87,787). The cell lysis buffer (Cat. no. R2002) was purchased from Biosesang. Protein 5X sample buffer (Cat. no. EBA-1052) was purchased from Elpis Biotech. Protein translation inhibitor cycloheximide (CHX; Cat. no. 239,765) was bought from Merck. Proteasomal inhibitor MG132 (Cat. no. S2619) was acquired from Selleckchem. Puromycin (Cat. no. 12,122,530) was purchased from Gibco, and the DUB inhibitor PR-619 (ab144641) was purchased from Abcam. The ubiquitin activating enzyme inhibitor MLN7243 (also called TAK243, Cat no. HY-100,487) was bought from Med Chem Express. Autophagy inhibitor chloroquine (Cat. no. C6628) and *all-trans* retinoic acid (Cat. No. R2500) was purchased from Sigma Aldrich. DAPI (Cat. no. H-1200) was purchased from Vector Laboratories.

### Cell culture and transfection

Human embryonic kidney (HEK293) (KCLB: 21,573), SH-SY5Y (KCLB: 22,266), and SK-N-SH (KCLB: 30,011) cells were purchased from the Korean Cell Line Bank (Seoul, South Korea). SK-N-DZ and SK-N-AS were kindly provided by Prof. Sung-Oh Huh from Hallym University, South Korea. SK-N-DZ, SK-N-AS and HEK293 cells were maintained in Dulbecco’s Modified Eagle Medium (DMEM) (PAN^™^ BIOTECH, P04-03590, Aidenbach, Germany) supplemented with 10% fetal bovine serum (FBS) (Gibco BRL, Cat. No. 10,082,147, Rockville, MD, USA) and 1% penicillin and streptomycin (Gibco BRL, Cat. No. 15,140,122, Rockville, MD, USA), while the SH-SY5Y and SK-N-SH cells were maintained in a 1:1 mixture of minimum essential medium (MEM): F12 medium supplemented with 10% FBS and 1% penicillin and streptomycin at 37 °C in a humidified atmosphere with 5% CO_2_.

The transfection of plasmids and shRNA in the HEK293 cell line was performed using polyethylenimine (Polysciences, Cat. no. 25449-100, Warrington, USA). The SK-N-DZ, SK-N-AS, SH-SY5Y, and SK-N-SH cells were transfected with Lipofectamine 3000 (Thermo Fisher Scientific Cat. no. L3000001, USA) according to the manufacturer’s protocol.

### Cell viability assay

For the cell proliferation assay, SH-SY5Y SK-N-SH, SK-N-AS and SK-N-DZ were seeded into 96-well plates at a density of 1 ⋅ 10^4^ cells/well post-transfection and incubated for 48 h. Next, 10 µL of CCK-8 assay reagent (Dojindo Molecular Technologies, Cat. No. CK04-11, MD, USA) was added to each well, and the absorbance was measured at 450 nm using a spectrophotometer (Bio-Rad Laboratories, Inc., Korea).

### T7 endonuclease 1 assay

The T7 endonuclease assay was performed as described previously [21, 22]. Briefly, genomic DNA was isolated using DNeasy Blood and Tissue kits (Promega, Madison, WI, USA) according to the manufacturer’s protocol. The region of DNA containing the nuclease target site was PCR-amplified using nested primers. Amplicons were denatured by heating and annealed to form heteroduplex DNA, which was then treated with 5 units of T7E1 (New England Biolabs, MA, USA) for 15 to 20 min at 37 °C, followed by 2% agarose gel electrophoresis. Mutation frequencies were calculated based on band intensity using ImageJ software and the following equation: mutation frequency (%) = 100 × (1 - [1 - fraction cleaved]^1/2^), where the fraction cleaved was the total relative density of the cleavage bands divided by the sum of the relative density of the cleaved and uncut bands. The oligonucleotide sequences used to obtain the PCR amplicons from the on-target genes for the T7E1 assay are listed in Supplementary Table S2. The oligonucleotide sequences used to obtain the PCR amplicons from the off-target genes for the T7E1 assay is previously described in [20]. The amplicon sizes of the USP3 and their expected cleavage sizes after the T7E1 assay are summarized in Supplementary Table S3.

### Quantitative reverse transcription PCR analysis

Total RNA was isolated using Trizol reagent (Favorgen, Kaohsiung, Taiwan). RNA pellets were resuspended in 30 µL of nuclease-free water, and the RNA concentration was measured. Next, 500 ng of total RNA was reverse transcribed using a SuperScript III First-Strand Synthesis System (Life Technologies, USA) with an oligo-dT primer according to the manufacturer’s protocol. Quantitative PCR was performed in triplicate using Fast SYBR Green I Master Mix (Life Technologies) and a Step One Plus Real-Time PCR System (Life Technologies). The gene specific oligonucleotide primers that were used for Real-Time PCR analysis are listed in Supplementary Table S4. The relative expression of each gene was normalized to GAPDH as a control for all experiments. We determined the relative quantification of the gene expression using the 2-ΔΔCq method [23].

### Generation of a *USP3*-knockout SH-SY5Y cell line using CRISPR/Cas9

The SH-SY5Y cells were co-transfected with a plasmid encoding Cas9-2 A-mRFP-2 A-PAC and sgRNA1 targeting USP3 or scrambled sgRNA (mock control) at a 1:2 ratio using Lipofectamine 3000 Reagent (Thermo Fisher Scientific, MA, USA) according to the manufacturer’s instructions. The next day, the cells were selected using puromycin (2 µg/mL) for 2 days. The selected cells were then seeded into 96-well plates at an average density of 25 cells/plate. Fifteen days after seeding, each well was microscopically evaluated, and single cell-derived colonies were selected. The selected colonies were trypsinized and reseeded into 24-well cell culture plates. A small portion of the cells was screened for USP3 gene disruption using the T7E1 assay. T7E1-positive clones were expanded and stored in a liquid nitrogen tank for later use. The USP3 gene disruption was confirmed by Sanger sequencing.

### Immunoprecipitation

For the co-IP assay, the SH-SY5Y cells were harvested 48 h post-transfection and lysed for 20 min in IP lysis buffer (25 mM Tris-HCl (pH 7.4), 150 mM sodium chloride, 1 mM EDTA, 1% NP-40, 5% glycerol, 1 mM PMSF, and protease inhibitor cocktail), and the amount of protein was estimated using Bradford reagent. Then, 2–3 mg of the cell lysates were immunoprecipitated with the respective antibodies at 4 °C overnight and incubated with 25 µL of protein agarose beads at 4 °C for 3 h. Before loading the samples on the SDS-PAGE gels, the beads were washed with lysis buffer and eluted in 2X sodium dodecyl sulfate (SDS) sample loading buffer (5X SDS sample loading buffer containing 4% SDS, 20% glycerol, 10% 2-mercaptoethanol, 0.004% bromophenol blue, and 0.125 M Tris-HCl [pH 6]). The samples were then boiled at 95–100 °C for 5 min and analyzed by western blotting. Mouse IgG (ab-99,697, 1:10000, Abcam) and rabbit IgG (CST-58,802 S, 1:10000, Cell Signaling Technology) light chain-specific secondary antibody was used to prevent interference from heavy and light immunoglobulin chains for the binding assay.

### Tandem ubiquitin-binding entities assay

The ubiquitination status of the REST protein was determined using a tandem ubiquitin binding entities (TUBEs) assay (Cat. no. UM402, LifeSensors, PA, USA) as previously described [24, 25]. Briefly, the mock control and Flag-USP3 transfected SH-SY5Y cells were pretreated with the proteasome inhibitor MG132 (10 µM/mL) for 6 h before harvesting for protein extraction. The harvested cells were lysed in IP lysis buffer containing 150 mM sodium chloride, 1% Triton X-100, 25 mM Tris (pH 7.5), 1 mM EDTA, 10% glycerol, and protease inhibitor cocktail. The whole-cell protein extracts were incubated with 20 µL of ubiquitin affinity matrices-TUBE2 at 4 °C for 3 h with rotation. The beads were washed three times with IP lysis buffer and eluted in a 30 µL 2X SDS sample loading buffer (5X SDS sample loading buffer containing 4% SDS, 20% glycerol, 10% 2-mercaptoethanol, 0.004% bromophenol blue, and 0.125 M Tris-HCl (pH 6.8)) and boiled at 95–100 °C for 5 min. The samples were then loaded onto SDS-PAGE gels and analyzed by western blotting.

### Deubiquitination assay

**Fig. 1:**
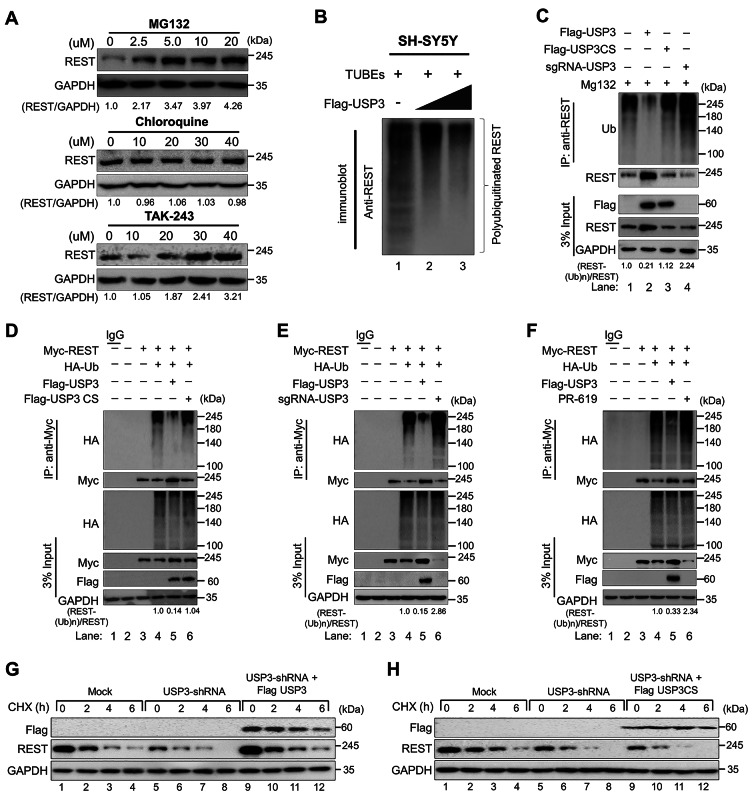
USP3 deubiquitinates REST and extends its half-life. **(A)** SH-SY5Y cells were treated with the indicated concentrations of proteasomal inhibitor (MG132), lysosomal inhibitor (CQ), or inhibitor of ubiquitin activating enzyme (TAK-243) for 6 h prior to harvest. Immunoblotting was performed with the indicated antibodies. The protein band intensities were estimated using ImageJ software with reference to the GAPDH control band (REST/GAPDH) and presented below the blots. **(B)** SH-SY5Y cells were transfected with increasing concentrations of Flag-USP3 and treated with MG132 for 6 h prior to harvest. A TUBEs assay was performed to assess the ubiquitination status of the REST protein in mock control and Flag-USP3 transfected cells. Cell lysates were immunoprecipitated with TUBEs antibodies, followed by immunoblotting with the indicated antibodies. **(C)** The ubiquitination and deubiquitination of endogenous REST were analyzed by transfecting SH-SY5Y cells with Flag-USP3, Flag-USP3CS, or sgRNA targeting *USP3* followed by immunoprecipitation (IP) with an anti-REST antibody and immunoblotting with an anti-ubiquitin antibody. The cells were treated with MG132 for 6 h prior to harvest. **(D)** The ubiquitination and deubiquitination of ectopically expressed Myc-REST were analyzed by transfecting HEK293 cells with Flag-USP3 and Flag-USP3CS **(E)** Flag-USP3 and sgRNA targeting *USP3***(F)** treatment of DUB-inhibitor PR-619 for 48 h in the HEK293 cell line prior to harvest, followed by IP with a Myc antibody and immunoblotting with an anti-ubiquitin antibody. The relative protein expression of REST-(Ub)n with respect to input REST for (C-F) was quantified using ImageJ software and represented as (REST-(Ub)n/REST) below the blot. **(G-H)** The knockdown effect of USP3 and reconstitution of either **(G)** Flag-USP3 or **(H)** Flag-USP3CS in USP3 depleted neuroblastoma on the half-life of endogenous REST in SH-SY5Y cells. CHX (150 µg/mL) was administered for the indicated time, and the cells were then harvested for western blotting with the indicated antibodies

The DUB activity of USP3 on endogenous and ectopically expressed REST protein ubiquitination was assessed in SH-SY5Y and HEK293 cells, respectively, by an in vitro deubiquitination assay. After 48 h, cells were treated with MG132 (10 µM/mL for 6 h) and harvested for protein extraction. The cells were lysed for 20 min in denaturing lysis buffer containing 150 mM sodium chloride, 1% Triton X-100, 1% sodium deoxycholate, 1% SDS, 50 mM Tris-HCl (pH 7.4), 2 mM EDTA, 1 mM PMSF, and protease inhibitor cocktail. Then, 2–3 mg of cell lysates was immunoprecipitated with the respective antibodies at 4 °C overnight and incubated with 25 µL of protein agarose beads for 2–3 h at 4 °C. The beads were then washed with lysis buffer and eluted in 2X SDS sample loading buffer and boiled at 95–100 °C for 5 min. The samples were then loaded onto SDS-PAGE gels and analyzed by western blotting using ubiquitin and HA-antibodies. To confirm the specificity of REST ubiquitination and to avoid non-specific binding of polyubiquitin molecules to the REST protein, protein-bound beads were washed with stringent lysis buffer containing 300 mM NaCl for the experiments represented in Fig. 1.

### Immunofluorescence

Neuroblastoma cells were grown on glass coverslips and incubated at 37 °C in a humidified atmosphere with 5% CO_2_. After a wash with phosphate-buffered saline (PBS) (Gibco, Cat. No. 10,010,023, Scotland, United Kingdom) the cells were fixed using 4% paraformaldehyde (PFA, PC2031-100-00, Biosesang) for 15 min and permeabilized in PBS containing 0.1% Triton X for 5 min. The cells were then thoroughly washed in PBS and blocked with 5% bovine serum albumin, followed by incubation with appropriate primary antibodies overnight at 4 °C. The next day, the cells were washed and incubated with the appropriate Alexa Fluor 488/594-conjugated secondary antibodies, or with Alexa Fluor^™^ 568 phalloidin (Invitrogen^™^, A12380) for 1 h. The cells were then incubated with DAPI and mounted using VectaShield (Vector Laboratories, H-1000-10, CA, USA). The cells were visualized, and images were produced using a Leica fluorescence microscope (Leica, DM 5000B; Leica CTR 5000; Wetzlar, Germany).

### Duolink proximity ligation assay

The interaction between USP3 and REST was assessed using a Duolink in situ proximity ligation assay (PLA) kit (Cat. no. DUO92101, Sigma Aldrich). SH-SY5Y cells were fixed in 4% PFA for 10 min at room temperature and then blocked with blocking solution. The cells were treated with primary antibodies targeting USP3 and REST for 1 h at 37 °C, followed by incubation with PLA probes for 1 h at 37 °C in a humidified chamber. After three washes, a ligation-ligase solution was added, and the cells were incubated for 30 min at 37 °C. The slides were incubated for 100 min in an amplified polymerase solution at 37 °C in the dark. Finally, the cells were stained with mounting medium containing DAPI. A Leica fluorescence microscope was used to capture the images (Leica, DM 5000B, Leica CTR 5000, Wetzlar, Germany).

### RA-induced neural differentiation

For the RA-induced neural differentiation assay, neuroblastoma cells were treated with 10 µM *all trans*-RA (Sigma-Aldrich, R2625, USA) and dissolved in dimethyl sulphoxide (DMSO) (Sigma-Aldrich, D2650, USA) for 1, 3, 5 and 7 days. Cells treated with pure DMSO were used as a negative control. Bright field microscopy images were taken to study the morphological changes of the cells during differentiation.

### Neurosphere formation and limiting dilution assay

Neuroblastoma cells transfected with mock control, USP3-shRNA, and USP3-shRNA reconstituted with Flag-USP3 cells were seeded into low-attachment 6-well plates at a density of 2 ⋅ 10^4^ cells/well in neurosphere forming medium containing a 1:1 mixture of MEM and F12 medium supplemented with 10% FBS, 1% penicillin and streptomycin, 1% B27™ supplement (Gibco BRL, Cat. No. 17,504,044, Rockville, MD, USA), 1% N2 supplement (Gibco BRL, Cat. No. A1370701, Rockville, MD, USA), 20 ng/mL epidermal growth factor (hEGF) (Sigma-Aldrich, E2645, USA), 20 ng/mL basic fibroblast growth factor (bFGF) (Sigma-Aldrich, GF003AF, USA), and 0.1 mM β-mercaptoethanol (Gibco BRL, Cat. No. 31,350,010, Rockville, MD, USA). The cells were incubated at 37 °C in a humidified atmosphere with 5% CO_2_ for 2 weeks.

A neurosphere-forming, limiting dilution assay was performed as described previously [26]. Briefly, neuroblastoma transfected with mock control, USP3-shRNA, and USP3-shRNA reconstituted with Flag-USP3 cells were dissociated into single cells and seeded in low-attachment 96-well plates at a cell density of 1, 2, 5, 10 or 50 cells per well in neurosphere forming media. After 2 weeks of incubation at 37 °C in a humidified atmosphere with 5% CO_2_, the wells were checked for neurosphere formation. The neurosphere forming, limiting dilution assay was evaluated using the online application ELDA: Extreme Limiting Dilution Analysis available at http://bioinf.wehi.edu.au/software/elda [27].

### Soft agar assay

Neuroblastoma cells transfected with mock control, USP3-KO, and USP3-KO clones reconstituted with USP3 or REST were subjected to a colony formation assay. First, 1% agarose gel and 1X complete DMEM were mixed at a ratio of 1:1 and plated onto 35 mm culture dishes. The plates were then incubated overnight. The next day, cells resuspended in 0.75% agarose with DMEM (1:1 ratio) were seeded at a density of 1 ⋅ 10^4^ cells per well and incubated for 14 days. Crystal violet dye (0.01%) diluted in 20% methanol was used to stain the anchorage-independent colonies, which were counted using a light microscope (IX71, Olympus, Tokyo, Japan).

### Wound healing assay

Migration behavior was analyzed using a wound healing assay. Neuroblastoma cells transfected with mock control, USP3-KO, and USP3-KO clones reconstituted with USP3 or REST cells were cultured to near 90% confluence. Scratches were made in the monolayers with a sterile pipette tip in a definite array. The wounded cell layer was washed with PBS and incubated at 37 °C with 5% CO_2_. Wound closure was compared between conditions at 0 and 24 h using a light microscope and quantified using ImageJ software.

### Transwell cell invasion assay

Cellular invasion was assessed using 0.8 μm Transwell chambers coated with Matrigel for 1 h at 37 °C (Corning, NY, USA) according to the manufacturer’s instructions. Briefly, neuroblastoma cells transfected with mock control, USP3-KO, and USP3-KO clones reconstituted with USP3 or REST cells were seeded at a density of 3.0 ⋅ 10^4^ cells/well in 500 µL of serum-free DMEM in 24-well plates. The following day, the cells on the top surface of the insert were scraped off, and the cells on the bottom surface were fixed with ice-cold methanol followed by crystal violet staining. The number of cells was counted using light microscopy.

### Xenograft tumor experiment

Xenograft tumor experiments were performed in 5-week-old NOD SCID-gamma (NSG) mice. The animal study was approved by the Institutional Animal Care and Use Committees of Hanyang University. All mice were housed in a temperature-controlled room under standard conditions (12 h light/dark cycle and 55% relative humidity) with access to food and water *ad libitum*. SH-SY5Y cells transfected with mock control, USP3-KO, and USP3-KO clones reconstituted with USP3 or REST cells were prepared in DMEM: Matrigel (1:1) (BD Biosciences) and subcutaneously injected into the right flank of each mouse. The tumors were harvested at the end of the experiment and images were taken. Tumor growth was recorded by measuring two perpendicular diameters (short axis and long axis), and tumor volume was calculated using the formula V = D × d^2^ × 0.5, where D and d are the long and short axes of the tumor, respectively. For all animal studies, the animals were chosen randomly.

### Immunohistochemistry

The tumor-tissue samples obtained from the mice were fixed with 4% PFA and embedded in paraffin. Formalin-fixed paraffin-embedded (FFPE) tissues were then sectioned at a thickness of 5 μm and stained with USP3 and REST antibodies following the manufacturers’ recommendations. The samples were counterstained with hematoxylin, dehydrated, and mounted. All images were taken using a Leica DM5000 B microscope (Leica, Germany).

### Statistical analysis

Statistical analysis and graphical presentation were performed using GraphPad Prism 9.0. No statistical method was used to predetermine the sample size. All results are presented as the means and standard deviations of at least three independent experiments (unless otherwise stated in the figure legends). Comparisons between the two groups were analyzed using the Student’s *t*-test. Experiments involving three or more groups were analyzed by one-way or two-way analysis of variance (ANOVA) followed by Tukey’s test.

## Results

### Genome-scale screening of the ubiquitin-specific protease subfamily for REST protein using the DUB-knockout library

**Fig. 2:**
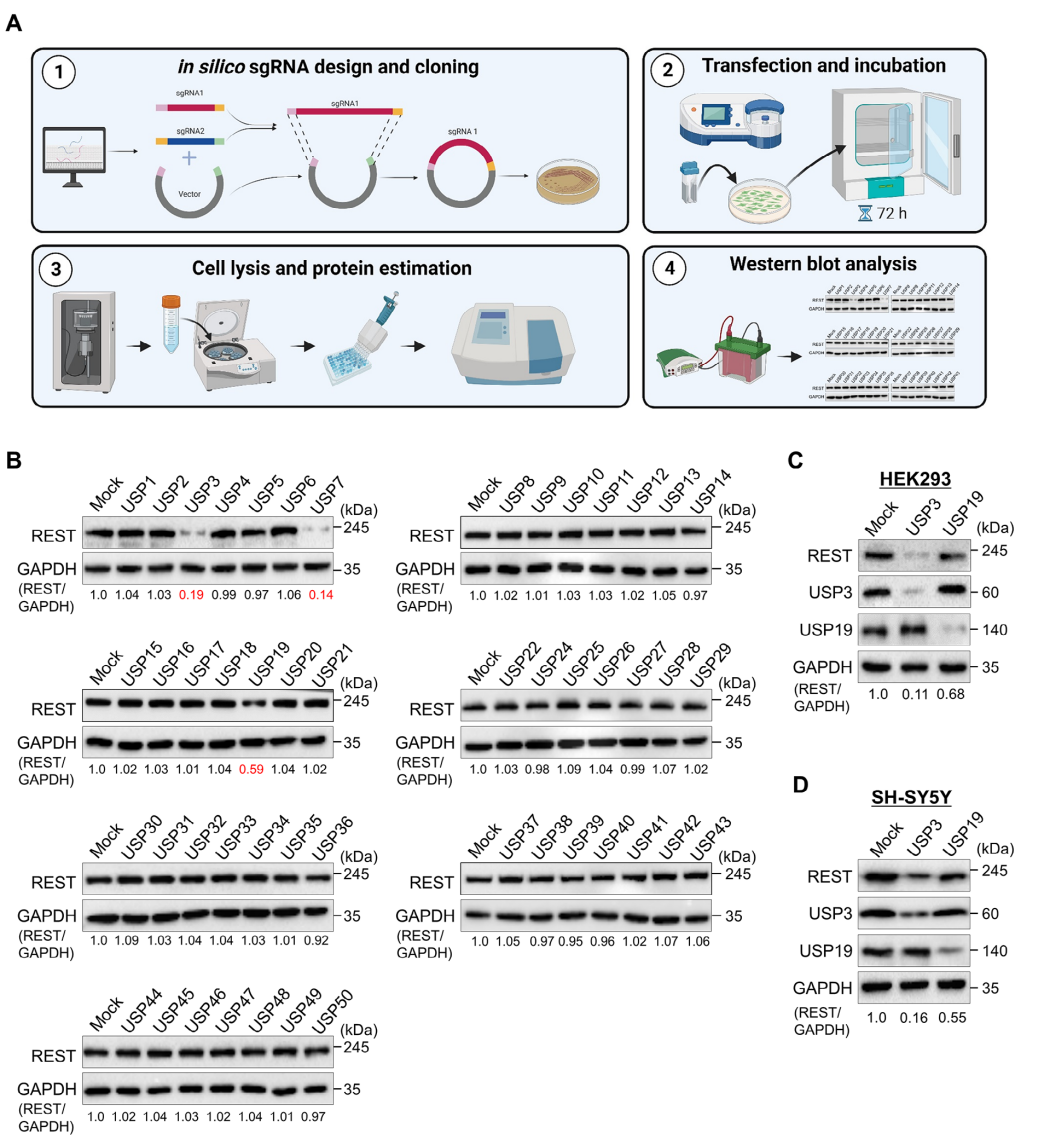
DUB knockout library-based screening for DUBs regulating REST protein level by Western blot analysis. **(A)** Schematic representation of CRISPR/Cas9-based sgRNA library screening for DUBs that alters REST protein levels. Steps 1–2: The designed DUB knockout sgRNA library, consisting of an entire set of genes encoding USPs, was co-transfected with Cas9 into HEK293 cells (day 1). The transfected cells were selected using puromycin (2 µg/mL) and incubated for 3 days at 37 °C in a humidified atmosphere with 5% CO_2_ (days 2–5). Step 3: The transfected cells were harvested and lysed in cell lysis buffer and protein was isolated. The protein concentration was estimated by Bradford protein assay. Step 4: Equal concentrations of all DUBKO cell lysates were loaded on SDS-PAGE and screened for DUB candidates regulating endogenous REST levels using western blot analysis. **(B)** The cell lysates having equal protein concentrations from DUB knockout library were subjected to western blotting to determine the endogenous REST protein level. For each blot, HEK293 cells co-transfected with scrambled sgRNA and Cas9 served as the mock control. GAPDH was used as a loading control. The protein band intensities were estimated using ImageJ software with reference to the GAPDH control for each individual sgRNA (REST/GAPDH) and presented below the blot. **(C)** The sgRNAs targeting putative DUB candidates and the REST protein level were estimated by western blotting in HEK293 cells. **(D)** Targeting putative DUB candidates by sgRNAs and its effect on the REST protein level were estimated by western blotting in SH-SY5Y cells. The protein band intensities were estimated using ImageJ software with reference to the GAPDH control band for each individual sgRNA (REST/GAPDH) and presented below the blot

To explore the post-translational regulation of REST protein, we used our recently developed CRISPR-Cas9 mediated DUB knockout library [20] to screen potential DUBs that regulate REST protein turnover. This library consists of 50 USP subfamily genes knocked out individually in HEK293 cell lines, where an equal concentration of all DUB knockout cell lysates was analyzed by western blotting (Fig. 2A). Any deviation in the endogenous REST protein level caused due to the loss-of-function of DUBs was considered as putative DUBs. From our screening, the loss of USP3, USP7, and USP19, compared to the mock control, was associated with a decrease in REST protein levels (Fig. 2B). USP7 was previously identified as DUB for REST protein involved in the maintenance of neural progenitor cells [9]. Next, we cross-confirmed the effect of putative DUBs on REST protein levels in both HEK293 cells and neuroblastoma cells. Our results showed that USP3 was the strongest DUB candidate among the other putative DUBs that showed a significant reduction in REST protein levels (Fig. 2C and D). Additionally, these putative DUBs regulating REST protein levels showed low cell viability in neuroblastoma cells when compared with some of the DUBs having no effect on the REST protein level (Supplementary Fig. 1). In line with a previous report [17], our result suggests that DUBs that alter REST protein levels may be a critical factor regulating cell proliferation in neuroblastoma.

### USP3 interacts with REST

**Fig. 3:**
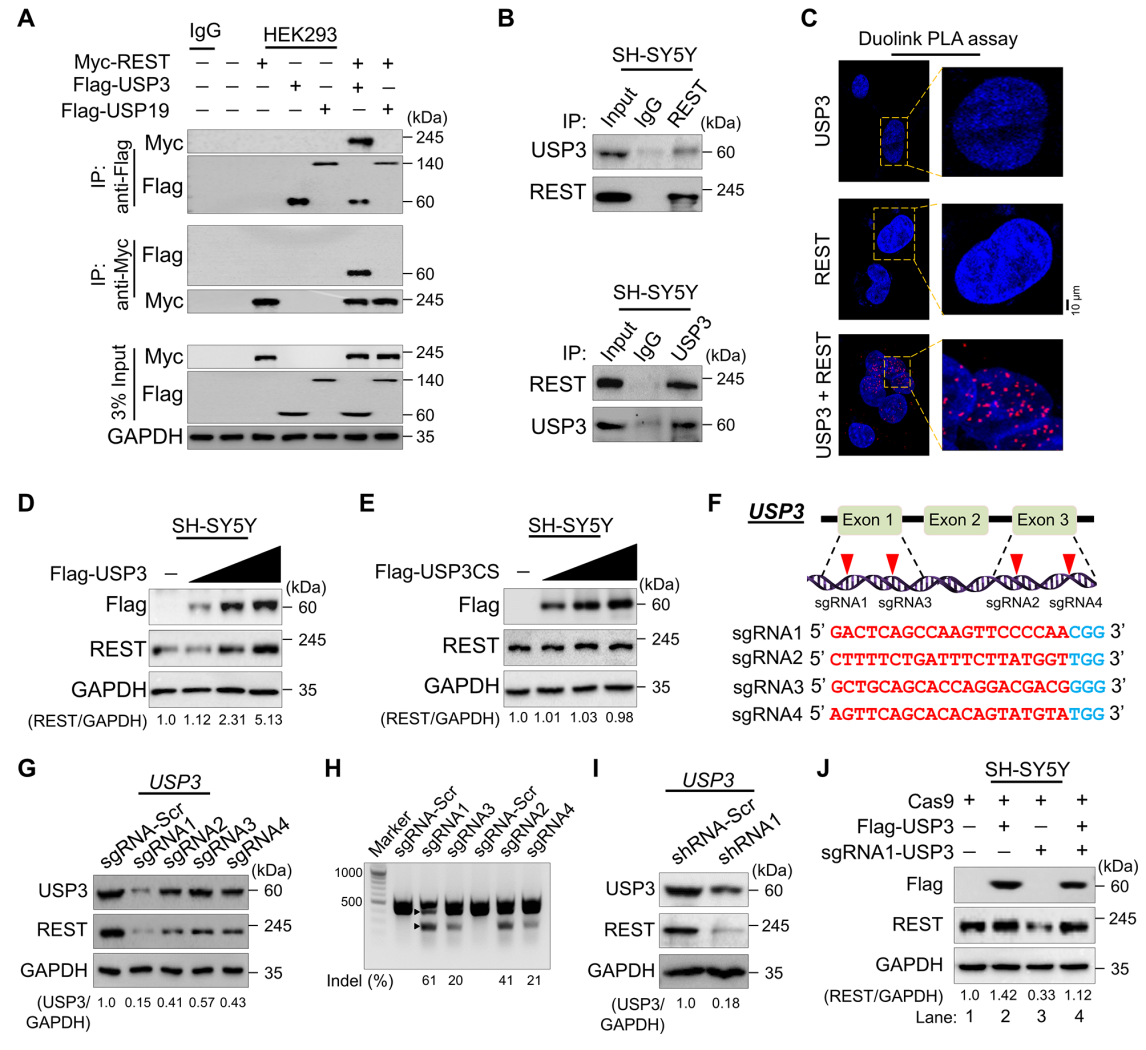
USP3 interacts with and increases REST protein levels. **(A)** The interaction between ectopically transfected Flag-USP3 or Flag-USP19 and Myc-REST proteins were analyzed in HEK293 cells. Cells were immunoprecipitated using indicated antibodies and USP3 and REST protein levels were checked by western blot. **(B)** The interaction between endogenous USP3 and REST was analyzed in SH-SY5Y cells. Cells were immunoprecipitated using the indicated antibodies and USP3 and REST protein levels were checked by western blot. **(C)** SH-SY5Y cells were subjected to the Duolink PLA assay to analyze the interaction between USP3 and REST using specific antibodies. The in situ USP3-REST interaction (PLA dots) was observed when USP3 and REST were immunostained together but not when they were stained with individual antibodies. Scale bar: 10 μm. **(D)** SH-SY5Y cells were transfected with increasing concentrations of Flag-USP3 to validate its effect on endogenous REST protein levels. **(E)** SH-SY5Y cells were transfected with increasing concentrations of catalytic mutant Flag-USP3CS to validate its effect on endogenous REST protein levels. The protein band intensities for (D-E) were estimated using ImageJ software with reference to the GAPDH control band (REST/GAPDH) and presented below the blot. **(F)** Schematic representation of the sgRNAs targeting exon 1 and 3 of the *USP3* gene. Red arrowheads indicate the positions of sgRNAs that target the top strand. sgRNA sequences are in red font, and PAM sequences are in bold blue font. **(G)** The efficiency of sgRNAs targeting USP3 by transient co-transfection with Cas9 in SH-SY5Y cells and immunoblotting with USP3 and REST antibodies. The protein band intensities were estimated using ImageJ software with reference to the GAPDH control (USP3/GAPDH) and presented below the blot. **(H)** The efficiency of sgRNAs targeting USP3 by transient co-transfection with Cas9 in SH-SY5Y cells followed by a T7E1 assay to determine the cleavage efficiency. Samples were resolved on 2% agarose gel. The cleaved band intensity obtained from the T7E1 assay were measured (indel %) using ImageJ software. Scrambled sgRNA transfected SH-SY5Y cells were used as control cells. The black arrowhead indicate the cleaved PCR amplicons. **(I)** SH-SY5Y cells were transfected with shRNA targeting USP3, and the endogenous protein levels of USP3 and REST were checked by western blotting. The protein band intensities were estimated using ImageJ software with reference to the GAPDH control band (USP3/GAPDH) and presented below the blot. **(J)** The effect of reconstitution of Flag-USP3 on endogenous REST protein in USP3-depleted SH-SY5Y cells was validated. The protein band intensities were estimated using ImageJ software with reference to the GAPDH control band (REST/GAPDH) and presented below the blots

To examine whether the putative DUB candidates and REST protein physically associate in vivo, we performed a co-IP assay. An anti-Flag antibody co-immunoprecipitated ectopically expressed Myc-REST along with Flag-USP3 and vice-versa in HEK293 cells (Fig. 3A), indicating that USP3 interacts with REST. Interestingly, Flag-USP19 did not show any interaction with Myc-REST (Fig. 3A), suggesting that the effect of USP19 on the reduced REST protein level may be indirect. Moreover, co-IP analysis using antibodies against endogenous USP3 or REST demonstrated that endogenous USP3 interacts with REST protein and vice versa under the physiological conditions in neuroblastoma cells (Fig. 3B). Furthermore, Duolink PLA was used to visualize in situ interaction between USP3 and REST using a standard immunofluorescence staining assay. Duolink analysis confirmed the interaction between USP3 and REST by showing the fluorescence signals (PLA dots), but no PLA dots were observed when neuroblastoma cells were stained with either USP3 or REST antibody alone (Fig. 3C). These results suggest that USP3 interacts with REST protein both exogenously and endogenously.

### USP3 increases REST protein levels

To validate whether USP3 could increase the REST protein levels, we transfected the neuroblastoma cells with an increasing concentration of Flag-USP3 and catalytic mutant Flag-USP3CS. The increasing concentration of Flag-USP3 showed an increase in endogenous REST protein levels in a dose-dependent manner (Fig. 3D). In contrast, overexpression of Flag-USP3CS did not affect the REST protein levels (Fig. 3E), suggesting that the catalytic activity of USP3 increases REST protein levels. To elucidate the molecular functions of USP3 in neuronal tumorigenesis, we further applied both the sgRNA and shRNA systems to deplete USP3 expression. To this end, we designed four sgRNAs targeting exon1 and exon3 of USP3 (Fig. 3F) and shRNA targeting USP3 to silence USP3 gene expression. Transient transfection of sgRNA1 targeting USP3 showed a substantial decrease in USP3 and REST protein levels compared to all other sgRNAs (Fig. 3G), which was in line with the higher indel percentage mediated by sgRNA1 (Fig. 3H). Additionally, shRNA targeting USP3 also showed a reduction in REST protein level (Fig. 3I). Moreover, the reduced endogenous expression of the REST protein level in USP3-depleted cells was rescued by reconstitution with Flag-USP3 (Fig. 3J, lane 4 vs. 3). These results indicate that USP3 is a bona fide DUB stabilizing REST protein levels.

### USP3 prevents REST protein degradation and extends its half-life

Next, we checked whether REST protein undergoes degradation via the ubiquitin-mediated 26 S proteasomal degradation pathway or lysosomal pathway. To this end, we treated the cells either with proteasome inhibitor MG132 or lysosome inhibitor chloroquine (CQ) in a dose-dependent manner and analyzed the REST protein levels. Our results showed that a dose-dependent increase of MG132 increased the REST protein levels (Fig. 1A, upper panel). However, an increase in CQ concentration failed to affect REST protein levels (Fig. 1A, middle panel). Additionally, we treated the cells with TAK-243, an inhibitor of an ubiquitin activating enzyme that regulates the ubiquitin conjugation cascade in the proteasomal pathway. A dose-dependent treatment of cells with TAK-243 increased the REST protein level (Fig. 1A, lower panel), indicating that REST protein abundance is regulated via the proteasome degradation pathway.

Next, we analyzed the deubiquitinating activity of USP3 on both endogenous and exogenous REST protein degradation in neuroblastoma and HEK293 cells, respectively. To this end, we performed a ubiquitination of endogenous REST protein in neuroblastoma cells by a tandem ubiquitin binding entities (TUBEs) assay. Our results showed that REST undergoes polyubiquitination, showing a higher ubiquitin-conjugated smear (Fig. 1B, lane 1 and 3C, lane1). However, an increase in the concentration of USP3 significantly reduced the intensity of the ubiquitin-conjugated smear from the endogenous REST protein (Fig. 1B, lane 2–3 and 3C lane 2). However, catalytic mutant USP3CS did not show any deubiquitinating activity on REST protein (Fig. 1C, lane 3). In contrast, depletion of USP3 by sgRNA showed an increase in the ubiquitination smear from the endogenous REST protein (Fig. 1C, lane 4). Additionally, the deubiquitination assay on the exogenous REST protein in the HEK293 cells showed no deubiquitinating activity by catalytic mutant USP3CS (Fig. 1D, lane 6), while sgRNA targeting USP3 showed an increase in the REST ubiquitination (Fig. 1E, lane 6). We further treated the cells with a DUB inhibitor (PR-619) to assess the activity of USP3. The DUB inhibitor decreased the deubiquitinating activity of USP3 on the REST protein as is evident by the increased conjugated ubiquitin smear on REST (Fig. 1F, lane 6). Overall, our results demonstrate that USP3 regulates REST protein level by counteracting its ubiquitin-mediated proteasomal degradation.

To determine the functional consequence of the deubiquitinating activity of USP3 on REST protein turnover, we treated cells with the protein synthesis inhibitor cycloheximide (CHX) in the presence of Flag-USP3 or catalytic mutant Flag-USP3CS. Our results showed that the transient overexpression of USP3 significantly extended the half-life of endogenous REST protein when compared with the mock control (Supplementary Fig. 2, lanes 5–8). However, USP3CS failed to modulate the REST protein half-life (Supplementary Fig. 2, lanes 9–12). Moreover, knockdown of USP3 showed a significant reduction in the half-life of REST protein (Fig. 1G, lanes 5–8), which was then rescued by reconstitution with Flag-USP3 (Fig. 1G, lanes 9–12) but not with Flag-USP3CS (Fig. 1H, lanes 9–12). Taken together, these data suggest that USP3 prevents REST protein degradation and regulates protein turnover by its deubiquitinating activity.

### *In silico* identification of USP3 as a potential prognostic factor in neuroblastoma

**Fig. 4:**
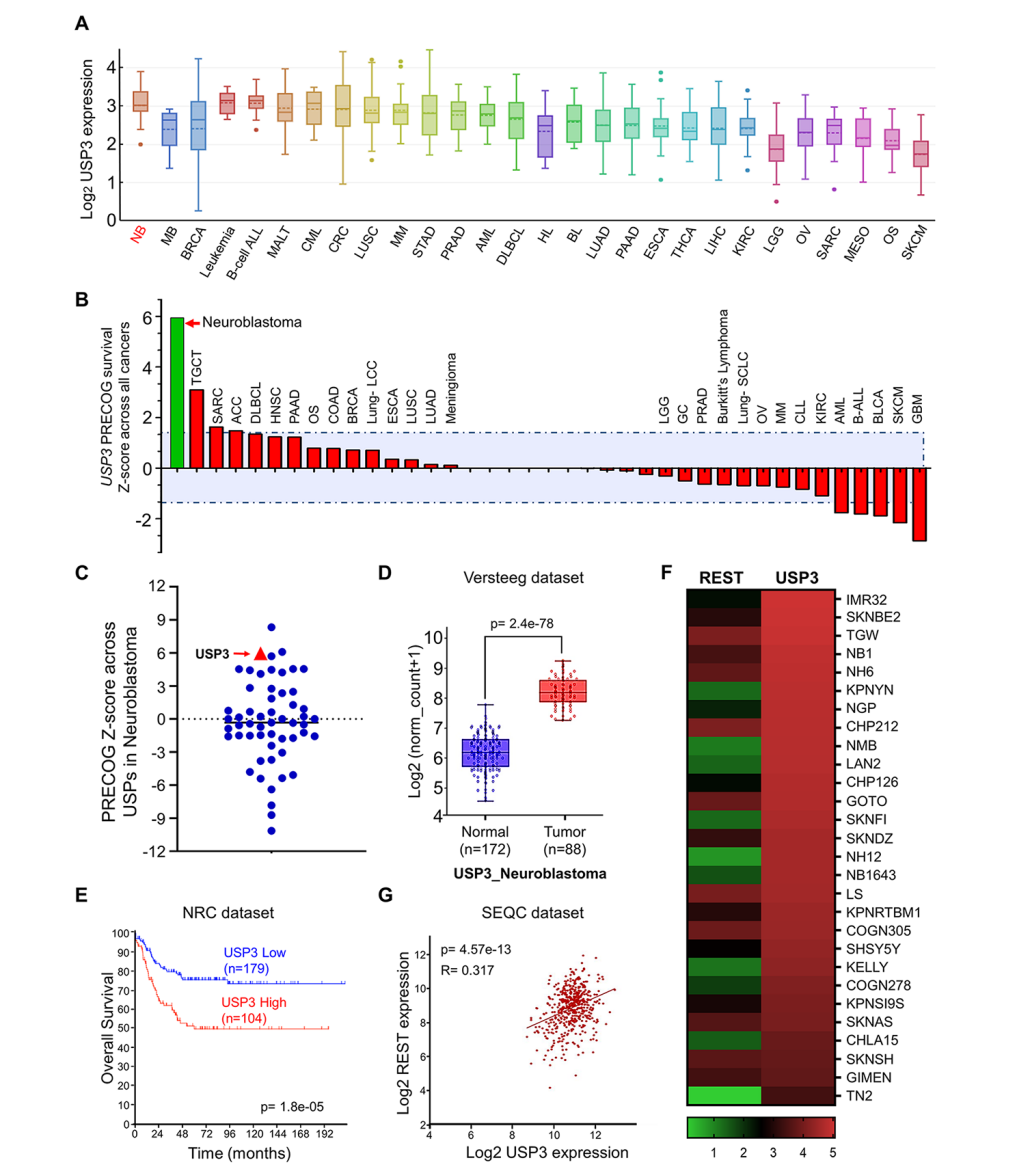
USP3 is associated with poor prognosis and survival in neuroblastoma patients. **(A)** Pan-cancer comparison analysis of USP3 mRNA expression in several cancer types using the Cancer Cell Line Encyclopedia (CCLE) database. NB: Neuroblastoma; MB: Medulloblastoma; BRCA: Breast cancer; ALL: Acute lymphoblastic leukemia; MALT: mucosa associated lymphoid tissue; CML: Chronic myelogenous leukemia; CRC: Colorectal cancer; LUSC: Lung squamous cell carcinoma; MM: Multiple myeloma; STAD: Stomach adenocarcinoma; PRAD: Prostate adenocarcinoma; AML: Acute myeloid leukemia; DLBCL: Diffuse large B-cell lymphoma; HL: Hodgkin’s lymphoma; BL: Burkitt lymphoma; LUAD: Lung adenocarcinoma; PAAD: Pancreatic adenocarcinoma; ESCA: Esophageal carcinoma; THCA: Thyroid carcinoma; LIHC: Liver hepatocellular carcinoma; KIRC: Kidney renal clear cell carcinoma; LGG: Brain low grade glioma; OV: Ovarian serous cystadenocarcinoma; SARC: Ewings sarcoma; MESA: Mesothelioma; OS: Osteosarcoma; SKCM: Skin cutaneous melanoma. The box plots were generated using Xenabrowser (https://xenabrowser.net/). **(B)** Prediction of Clinical Outcomes from Genomic Profile (PRECOG) meta Z-score of USP3 across different cancer subtypes using a cutoff of p < 0.05 and a median Z-score > 1.4 and <-1.4. TGCT: Tenosynovial giant cell tumor; SARC: Ewings sarcoma; ACC: Adenoid cystic carcinoma; DLBCL: Diffuse large B-cell lymphoma; HNSC: Head and neck squamous cell cancer; PAAD: Pancreatic adenocarcinoma; OS: Osteosarcoma; COAD: Colon adenocarcinoma; BRCA: Breast cancer; LCC: large cell carcinoma; ESCA: Esophageal carcinoma; LUSC: Lung squamous cell carcinoma; LUAD: Lung adenocarcinoma; LGG: Brain low grade glioma; GC: Gastric cancer; PRAD: Prostate adenocarcinoma; SCLC: Lung small cell carcinoma; OV: Ovarian serous cystadenocarcinoma; MM: Multiple myeloma; CLL: Chronic lymphocytic leukemia; KIRC: Kidney renal clear cell carcinoma; AML: Acute myeloid leukemia; ALL: Acute lymphoblastic leukemia; BLCA: Bladder urothelial carcinoma; SKCM: Skin cutaneous melanoma: GBM: Glioblastoma multiforme. **(C)** PRECOG meta Z-score for expression of USP family genes in neuroblastoma cancer, p < 0.001. **(D)** Box plot showing the difference between USP3 expression in normal (n = 172) and tumor (n = 88) tissues using the Versteeg neuroblastoma dataset: p-value = 2.4 e^− 78^. The box plot was generated using R2: genomic Analysis and Visualization Platform (https://r2.amc.nl) **(E)** Kaplan-Meier analysis showing overall survival probability of groups with low USP3 expression (n = 179) and high USP3 expression (n = 104) group in a neuroblastoma primary (NRC) dataset consisting of 283 primary neuroblastoma tumors: p-value = 1.8 e^− 05^. The overall survival graphs was generated using R2: genomic Analysis and Visualization Platform (https://r2.amc.nl) **(F)** A heat map showing mRNA expression levels of REST and USP3 in neuroblastoma cell lines derived from the CCLE database. Representative samples are arranged from high to low mRNA levels of USP3, and corresponding REST values are sorted. **(G)** A scatterplot showing the expression between USP3 and REST mRNA levels in SEQC dataset: p-value = 4.57e^− 13^. Pearson correlations (r) quantifying the relationship between USP3 and REST is given. The scatterplot between REST and USP3 mRNA expression was generated using R2: genomic Analysis and Visualization Platform (https://r2.amc.nl)

To further interrogate the role of USP3 in brain cancer, we used the Cancer Cell Line Encyclopedia (CCLE) (RNA-seq) dataset to evaluate the expression of USP3 in different types of cancer. Interestingly, the USP3 mRNA levels were observed to be significantly higher in the neuroblastoma cell lines when compared with other cancer cell lines (Fig. 4A). In addition, we evaluated the expression of *USP3* and *REST* in different cancer tissues using the Correlation AnalyzeR [28]. We found that *USP3* was highly expressed in cancers, including brain cancer, compared to normal tissues (Supplementary Fig. 3A). Similarly, *REST* expression was also higher in brain cancer compared to normal tissues (Supplementary Fig. 3B). Then, we used the Prediction of Clinical Outcomes from Genomic Profile (PRECOG) database [29] to analyze the impact of USP3 gene expression on patient survival in 39 classes of cancers, wherein positive z-scores referred to high USP3 gene expression, while a negative z-score referred to low USP3 gene expression that correlated with a poor outcome of patient survival. We profiled the meta z-scores for USP3 gene expression across 39 different types of cancers using a cutoff of p < 0.05 (median z-score of < -1.4 or > + 1.4). We observed that the impact of USP3 gene expression was highest with a z-score of + 5.95 (p < 0.001) in neuroblastoma, indicating that high expression of the USP3 gene may be associated with poor outcomes in neuroblastoma patients (Fig. 4B, Supplementary Table S5). Based on the high expression of USP3, especially in neuroblastoma cancers, we analyzed the expression level of all the USP family genes specifically in neuroblastoma cancer. USP3 was one of the most highly expressed DUB candidates in neuroblastoma (Fig. 4C, Supplementary Table S6).

We analyzed the expression of USP3 using two individual publicly available neuroblastoma datasets (Versteeg, and Hiyama). The *USP3* mRNA expression level was significantly higher in the neuroblastoma compared to the normal brain tissue samples (Fig. 4D and Supplementary Fig. 3C), suggesting that USP3 is aberrantly expressed in neuroblastoma tissues. Furthermore, we performed a Kaplan–Meier analysis using four different datasets, namely, (NRC, TARGET, SEQC and Kocak) in the neuroblastoma. The patient group with a low USP3 mRNA expression level was significantly associated with better overall survival (OS), whereas the high USP3 mRNA expression level was significantly associated with poor OS (Fig. 4E and Supplementary Fig. 4A). Together, these data suggest that USP3 is a potential prognostic marker in neuroblastoma.

### A correlation between USP3 and REST in neuroblastoma

Next, we analyzed the correlation between *USP3* and *REST* using the DepMap portal from an array of neuroblastoma cancer cell lines. We observed that high scores for *USP3* mRNA expression corresponded to high mRNA expression of *REST* in a majority of the neuroblastoma cell lines (Fig. 4F, Supplementary Table S7). Furthermore, a scatterplot of the *USP3* and *REST* mRNA expression patterns obtained from the SEQC and TARGET datasets produced r-values of 0.317 and 0.217, respectively (Fig. 4G and Supplementary Fig. 4B), suggesting a positive correlation between USP3 and REST in neuroblastoma tumors.

### Silencing of USP3 affects neuroblastoma self-renewal and cell proliferation by downregulating REST protein level

**Fig. 5:**
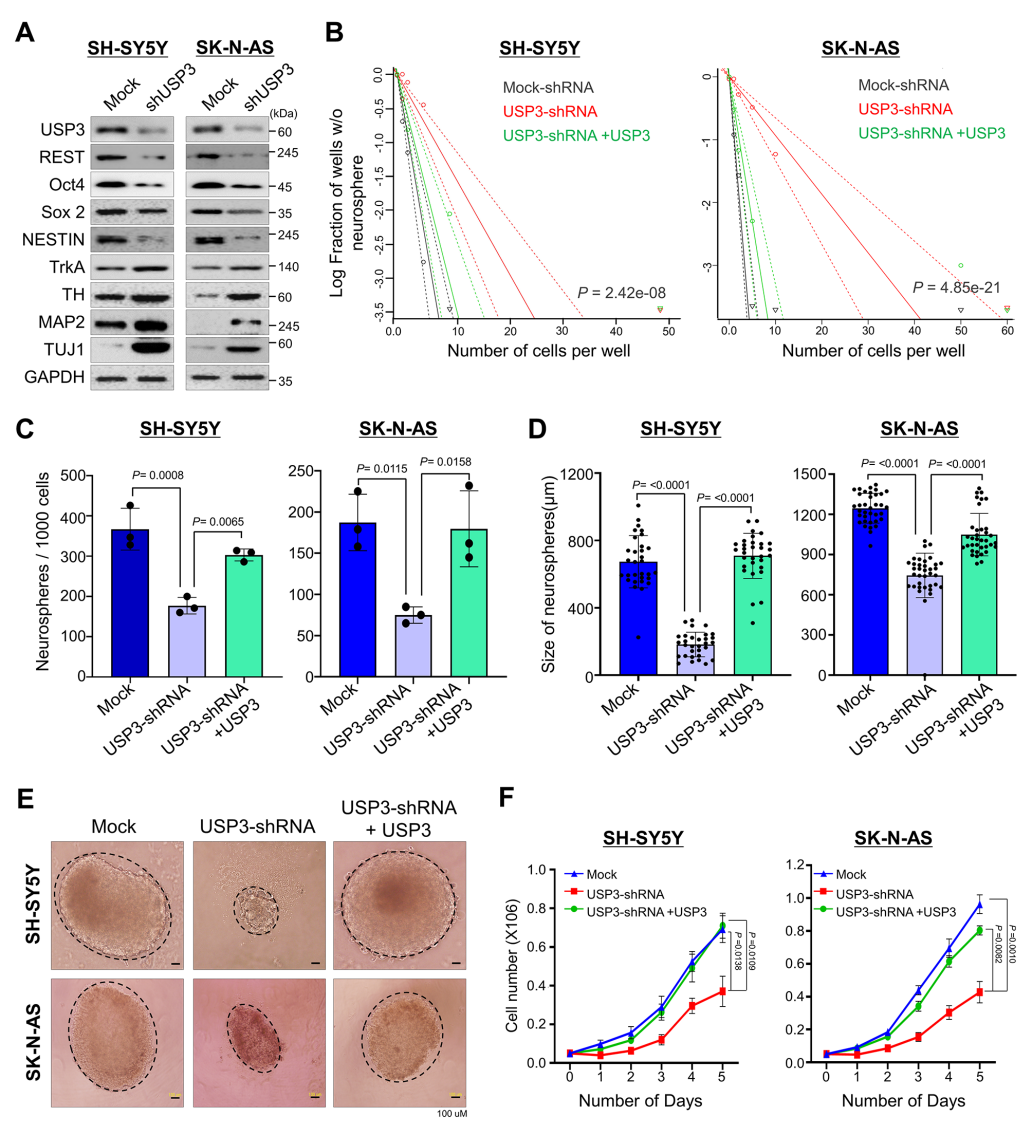
Depletion of USP3 affects self-renewal and cell proliferation in neuroblastoma. SH-SY5Y and SK-N-AS neuroblastoma cells transiently transfected with scrambled shRNA (Mock) or USP3 shRNA and USP3-depleted cells reconstituted with USP3 were used to perform the following experiments. **(A)** Western blot analysis to validate effect of USP3 knockdown on the expression of self-renewal markers in neuroblastoma using the indicated antibodies. GAPDH was used as the loading control. **(B)** The cells from (A) were used to perform an in vitro neurosphere-forming limiting dilution assay. The cells were dissociated and seeded in low-attachment 96-well plates at cell densities of 1, 2, 5, 10, and 50 cells/well and cultured for 2 weeks in neurosphere forming media. The frequency of neurosphere forming ability was evaluated using ELDA. Data are presented as the mean and standard deviation of at least three independent experiments. The significance of the difference between the indicated groups were determined by the χ^2^ test, and *P* values are indicated. **(C)** The cells from (A) were used to evaluate the effect of USP3 on the neurosphere forming ability in SH-SY5Y and SK-N-AS neuroblastoma cell lines. The cells were dissociated and seeded at density of 2 ⋅ 10^4^ and the number of neurospheres were quantified. Data are presented as the mean and standard deviation of three independent experiments (n = 3). One-way ANOVA followed by Tukey’s post hoc test was used, and *P* values are indicated. **(D)** The quantitative analysis of mean size of neurospheres after 2 weeks in neurosphere-forming media. Data are presented as the mean and standard deviation of three independent experiments (n = 3). One-way ANOVA followed by Tukey’s post hoc test was used, and *P* values are indicated. **(E)** Representative bright field microscopy images of the neurosphere formation of neuroblastoma cells expressing control shRNA, USP3 shRNA, and USP3 shRNA + USP3. Scale bar = 100 µM. **(F)** The SH-SY5Y and SK-N-AS cells from (A) were seeded in 6-well plates at density of 0.05 ⋅ 10^6^ cells/well. The cells were harvested and counted at indicated time points. Data are presented as the mean and standard deviation of three independent experiments (n = 3). Two-way ANOVA followed by Tukey’s post hoc test was used, and *P* values are indicated

We first investigated the USP3 knockdown effect on the ADR and MES protein markers in the SK-N-DZ and SK-N-AS cells. The mesenchymal protein markers such as Vimentin and PRRX1 were reduced upon USP3 knockdown (Supplementary Fig. 5A). Similarly, reduced expression of the adrenergic protein markers such as PHOX2A, PHOX2B, and DLK1 was displayed in USP3 knockdown cells (Supplementary Fig. 5A). Downregulation of REST protein level impairs self-renewal capacity and promotes neuronal differentiation in neuroblastoma [12, 13]. Thus, we investigated the impact of USP3-mediated downregulation of the REST protein level on self-renewal behavior in human neuroblastoma. For this purpose, we first checked the expression of USP3 and REST in the SK-N-AS, SK-N-DZ, SH-SY5Y, and SK-N-SH neuroblastoma cell lines. The expression of USP3 and REST was found in all of the neuroblastoma cell lines tested (Supplementary Fig. 5B). The shRNA-targeting USP3-transfected in the neuroblastoma cells showed a significant reduction in USP3 and REST protein level (Fig. 5A and Supplementary Fig. 6). A reduced expression level of Oct4, SOX2 and NESTIN was found, whereas TrkA, TH, MAP2 and TUJ1 was increased in USP3-silenced cells (Fig. 5A and Supplementary Fig. 6), suggesting that USP3 downregulation might impair self-renewal capacity in neuroblastoma.

Next, we investigated the effect of USP3 downregulation on clonogenic growth ability in neuroblastoma cells by performing an in vitro limiting, dilution neurosphere formation assay [30]. The neuroblastoma cell lines transfected with scrambled shRNA readily formed neurospheres, even at a seeding density of 1 or < 5 cells per well. In contrast, in USP3-silenced neuroblastoma cells, a seeding density of > 10 cells per well was required to form neurospheres in at least 50% of the wells. However, USP3-silenced neuroblastoma cell lines reconstituted with USP3 showed an improved neurosphere forming ability (Fig. 5B and Supplementary Fig. 7A). Moreover, USP3 downregulation in neuroblastoma cell lines significantly decreased the number (Fig. 5C and Supplementary Fig. 7B) and size of the neurosphere (Fig. 5D and Supplementary Fig. 7C). In addition, morphological variability was found (Fig. 5E and Supplementary Fig. 7D), suggesting that the downregulation of USP3 impedes neuroblastoma clonogenic growth. However, USP3-depleted neuroblastoma cells, when reconstituted with USP3, resulted in an increase in the number and size of the neurospheres (Fig. 5C and D and Supplementary Fig. 7B–7C) and were rescued from any morphological abnormalities in the neurospheres (Fig. 5E and Supplementary Fig. 7D). Additionally, USP3 downregulation caused a significant reduction in cell proliferation in the neurosphere culture. Conversely, USP3 over-expression in USP3-silenced cells increased the cell proliferation of neuroblastoma cells (Fig. 5F and Supplementary Fig. 7E). Overall, these results indicate that USP3 is critical in regulating REST protein level to maintain self-renewal and proliferation ability in neuroblastoma.

### Downregulation of USP3 induces neuroblastoma differentiation by promoting REST protein degradation

**Fig. 6:**
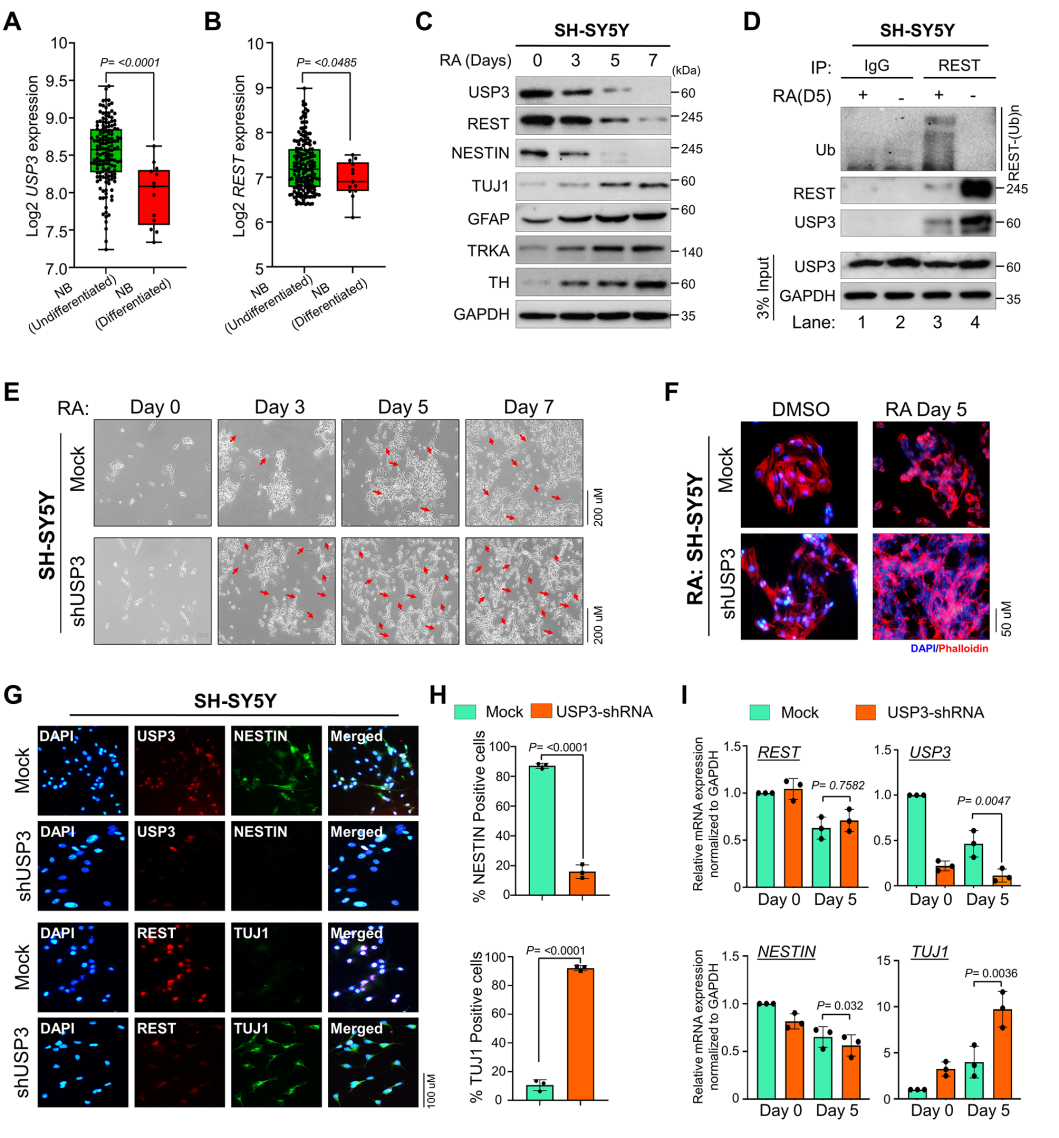
Depletion of USP3 promotes RA-induced neuronal differentiation in neuroblastoma cells. **(A-B)** Box plot showing the difference between (A) *USP3* and (B) REST mRNA expression levels in undifferentiated and differentiated neuroblastoma tissues in the TARGET neuroblastoma dataset. Data are presented as the mean and standard deviation. Student’s t-test was used, and *P* values are indicated. **(C)** The effect of RA-induced differentiation on USP3 and REST protein expression. The neuroblastoma cell lines were treated with 10 µM *all trans*-RA to undergo differentiation for the indicated time. The cells were harvested at a given time point and the effect of RA during neuroblastoma differentiation on USP3, REST, and differentiation marker proteins were assessed by western blot analysis. GAPDH was used as an internal loading control. **(D)** The effect of RA-induced differentiation on REST ubiquitination. The neuroblastoma cells were induced to differentiate by treating with 10 µM *all trans-* RA for 5 days, and then treated with MG132 for 6 h before harvest. Cell lysates were immunoprecipitated with anti-REST antibody and immunoblotting with an anti-ubiquitin antibody. **(E-F)** The morphology of the cells was examined under (E) a bright field microscope and (F) phalloidin staining showing depletion of USP3 promotes neurite outgrowth during RA-induced differentiation in neuroblastoma. The SH-SY5Y cells were either transfected with scrambled shRNA (Mock) or USP3 shRNA and treated with 10 µM *all trans*- RA for indicated days. The red arrow indicates neurite extension indicating differentiation. **(G)** The effect of RA-induced differentiation on neuroblastoma cells were evaluated by immunofluorescence analysis using indicated antibodies. **(H)** The immunofluorescence staining data from (G) was quantified. The number of TUJ1 and NESTIN positive cells were quantified in USP3-depleted cells through immunofluorescence analysis. Data are presented as the mean and standard deviation of three independent experiments. Student’s t-test was used, and *P* values are indicated. **(I)** The effect of USP3 depletion on mRNA expression levels of *REST*, *USP3, TUJ1*, and *NESTIN* during RA-induced differentiation of neuroblastoma cells were measured by quantitative real-time PCR. Data are presented as the mean and standard deviation of three independent experiments (n = 3). One-way ANOVA followed by Tukey’s post hoc test was used, and *P* values are indicated

We first analyzed the expression level of *USP3* and *REST* in undifferentiated and differentiated neuroblastoma using a TARGET dataset. The result showed that *USP3* and *REST* expression was high in undifferentiated neuroblastoma tumors when compared to well-differentiated neuroblastoma tumors (Fig. 6A and B). Several reports have suggested that downregulation of the REST protein level through ubiquitin-mediated proteasomal degradation induces neuronal differentiation [9, 12–14, 31]. Thus, we initially examined the expression level of USP3 along with REST during neuronal differentiation. To this end, we used RA, a clinically approved neuronal differentiation agent used in high-risk neuroblastoma patients, to induce differentiation in SH-SY5Y and SK-N-SH neuroblastoma cell lines. Both USP3 and REST proteins gradually decreased along with the NESTIN protein level, while showing an inverse correlation with the lineage commitment as measured by the expression level of neuronal differentiation markers such as TUJ1, GFAP, TrkA and TH (Fig. 6C and Supplementary Fig. 8A).

We next analyzed the ubiquitination status of REST to further gain insight into the role of USP3-mediated REST protein stabilization during differentiation of neuroblastoma cells. RA-induced differentiation of neuroblastoma cell lines showed low REST protein levels and increased REST polyubiquitinated smear when compared to RA-untreated SH-SY5Y and SK-N-SH neuroblastoma cell lines (Fig. 6D and Supplementary Fig. 8B). Interestingly, the USP3 protein level was also low in RA-induced differentiation of neuroblastoma cell lines when compared to the RA-untreated cells (Fig. 6D, lane 3 and Supplementary Fig. 8B, lane 4), suggesting that downregulation of USP3 may promote REST ubiquitination during neuroblastoma differentiation.

Furthermore, we examined the impact of shRNA-mediated USP3 knockdown on RA-induced neuronal differentiation by monitoring the morphological changes and estimating the expression of differentiation-related markers. The silencing of USP3 resulted in distinct morphological changes in SH-SY5Y, SK-N-SH and SK-N-DZ neuroblastoma cell lines, exhibiting a flatter and differentiated cell appearance with neurite-like processes compared to the scrambled-shRNA transfected neuroblastoma cells (Fig. 6E F and Supplementary Fig. 9 and 10 A-10B). Immunofluorescence staining demonstrated that the downregulation of USP3 resulted in a significant reduction in REST protein levels in SH-SY5Y (Fig. 6G), SK-N-SH (Supplementary Fig. 9B) and SK-N-DZ (and Supplementary Fig. 10C) neuroblastoma cell lines. In addition, downregulation of USP3 resulted in a reduction in the number of NESTIN-positive cells, while the number of TUJ1-positive cells increased significantly compared to scrambled shRNA transfected SH-SY5Y (Fig. 6G H), SK-N-SH (Supplementary Fig. 9B-9 C) and SK-N-DZ (and Supplementary Fig. 10C-10D) cell lines, indicating that silencing USP3 induces neuroblastoma differentiation. Additionally, RT-PCR analysis of TUJ1, a neuronal differentiation marker, showed a significant increase, whereas NESTIN, a neural stem cell marker showed a significantly decreased expression in USP3-silenced neuroblastomas (Fig. 6I). Altogether, our results suggest that USP3 is critical in maintaining an abundance of REST protein to determine neuroblastoma differentiation.

### Generation of single cell-derived USP3-knockout clones in human neuroblastoma cell lines

**Fig. 7:**
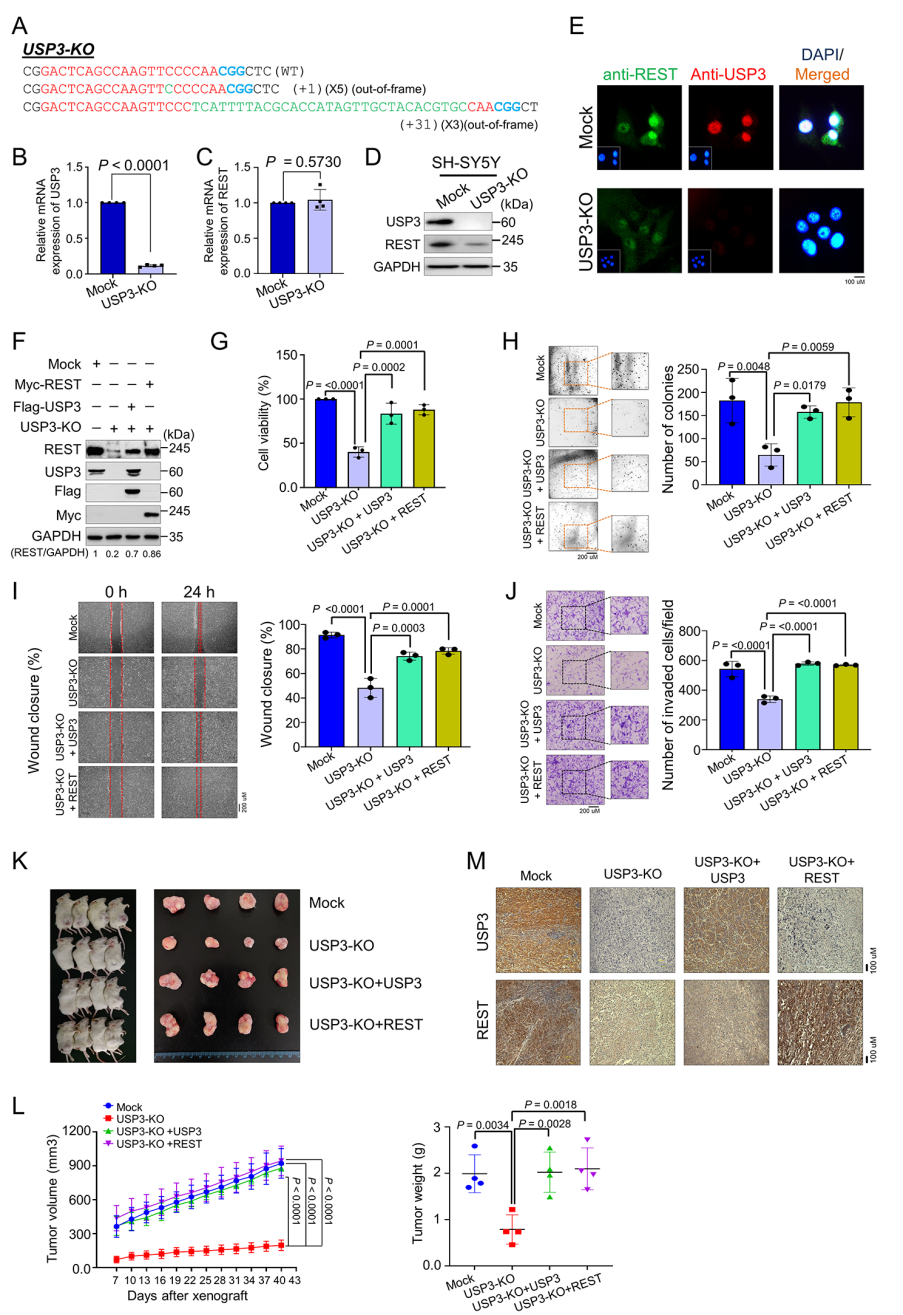
Loss of USP3 inhibits neuroblastoma tumorigenesis in vitro and in vivo. **(A)** Sanger sequencing data showing the disruption in *USP3* gene sequence in SH-SY5Y cells (USP3-KO). **(B)** The effect of USP3 KO on the mRNA expression of *USP3* and **(C)***REST* was evaluated by qRT-PCR with specific primers. The relative mRNA expression levels are shown after normalization to GAPDH mRNA expression. Data are presented as the mean and standard deviation of four independent experiments (n = 4). A two tailed t-test was used, and *P* values are indicated. **(D)** Western blot analysis of the endogenous expression of USP3 and REST protein in USP3-KO was evaluated. GAPDH was used as the internal loading control. **(E)** Immunofluorescence staining of the endogenous expression of USP3 and REST in USP3-KO cell line. **(F)** Mock control, USP3-KO, and USP3-KO cells reconstituted with either USP3 or REST. Western blot analysis to validate the expression of USP3 and REST using USP3- and REST-specific antibodies. The protein band intensities were estimated using ImageJ software with reference to the GAPDH control band (REST/GAPDH) and presented below the blot. The cells from (F) were subjected to following experiments **(G)** cell viability by CCK-8 assay, **(H)** colony formation assay, **(I)** wound-healing assay and **(J)** transwell cell-invasion assay. Data are presented as the mean and standard deviation of three independent experiments (n = 3). One-way ANOVA followed by Tukey’s post hoc test was used, and *P* values are indicated. **(K)** Xenografts were generated by subcutaneously injecting mock control, USP3 KO, and USP3-KO cells reconstituted with USP3 or REST SH-SY5Y cells into the right flanks of NOD SCID gamma mice (n = 4/group). Tumor volumes were recorded. The right panel shows the tumors excised from the mice after the experiment. **(L)** The tumor volume was measured every 3 days for 43 days and is presented graphically. The tumor weight was recorded post-euthanization. Data are presented as the mean and standard deviation of four independent experiments (n = 4). Two-way ANOVA followed by Tukey’s post hoc test was used, and *P* values are indicated. **(M)** Xenograft tumors were embedded in paraffin and sectioned. The immunohistochemical analyses were performed with the indicated antibodies. Scale bar = 100 μm

We generated single-cell-derived *USP3* KO clones using the CRISPR-Cas9 system in the SH-SY5Y neuroblastoma cell line, which is a well-established cell line for studying neuroblastoma tumor progression. The highly efficient sgRNA1-targeting *USP3* gene at exon1 along with Cas9 were transfected to generate *USP3* gene disruption, and the transfected cells were diluted into 96-well plates for single-cell clonal selection. The T7E1-positive single-cell derived SH-SY5Y USP3 KO clone #4 (hereafter USP3-KO) (Supplementary Fig. 11A) showed an out-of-frame mutation in the USP3 gene by Sanger sequencing (Fig. 7A). The RT-PCR analysis of USP3-KO showed a significant reduction in *USP3* mRNA levels compared with the mock control (Fig. 7B). However, loss of *USP3* did not show any significant changes in *REST* mRNA levels (Fig. 7C), indicating that USP3 does not regulate REST expression at the transcriptional level. Moreover, western blot analysis (Fig. 7D and Supplementary Fig. 11B) and immunofluorescence staining (Fig. 7E) demonstrated that the loss of USP3 in the neuroblastoma cell line showed a significant decrease in REST protein levels compared with the mock control.

### Loss of USP3 impairs the REST-mediated oncogenic potential of neuroblastoma in vitro and in vivo

To investigate the effect of USP3 on REST-mediated progression of SH-SY5Y neuroblastoma tumors, we used the USP3-KO clone showing a low REST protein level. This USP3-KO clone was reconstituted with either USP3 or REST (Fig. 7F) in a variety of assays related to carcinogenesis activity. USP3-KO showed a decrease in cell viability, and this decrease was reverted by reconstitution with either USP3 or REST (Fig. 7G). An anchorage-independent colony formation assay showed a significant reduction in colony numbers in the USP3-KO when compared with the mock control group (Fig. 7H). Similarly, the cellular migration and invasion assay showed that the depletion of USP3 significantly impeded migration and invasion ability compared to the mock control (Fig. 7I J). However, the cell migration, cell invasion, and colony formation were significantly restored when USP3-depleted neuroblastoma cells were reconstituted with either USP3 or REST (Fig. 7H J).

Additionally, we cross confirmed these carcinogenesis activities in USP3-silenced SK-N-SH neuroblastoma cells and reconstituted with either USP3 or REST (Supplementary Fig. 12). In line with our previous observation, USP3-silenced cells showed impeded cell proliferation (Supplementary Fig. 13A), anchorage-independent colony formation (Supplementary Fig. 13B), migration (Supplementary Fig. 13C), and invasion abilities compared to the mock control (Supplementary Fig. 13D). As expected, the USP3-silenced neuroblastoma cells reconstituted with either USP3 or REST regained its carcinogenic behavior (Supplementary Fig. 13A–13D). Similar impaired REST-mediated oncogenic behavior was observed in SK-N-AS (Supplementary Fig. 14A–14 C) and SK-N-DZ (Supplementary Fig. 15A–15 C) neuroblastoma cells.

Next, we performed a subcutaneous tumorigenesis assay to corroborate the role of USP3 on the REST-mediated oncogenic transformation in neuroblastoma in vivo. To this end, we subcutaneously injected the mock control cells, USP3-KO cells, and USP3-KO cells reconstituted with USP3 or REST from Fig. 7F into the right flank of NOD SCID γ (NSG) mice. The mice injected with USP3-KO cells displayed a significant reduction in tumor volume and weight compared to mice injected with mock cells (Fig. 7K L). However, transplantation of USP3-depleted cells reconstituted with either USP3 or REST resulted in a significant increase in tumor weight and volume (Fig. 7K L). Furthermore, the immunohistochemical (IHC) analysis of mouse xenograft tumor tissues showed a significant reduction in USP3 and REST expression in USP3-depleted tumors relative to mock control tumors. However, the expression of USP3 and REST was regained by reconstitution with USP3 or REST (Fig. 7M). Taken together, our results suggest that the loss of USP3 abolishes REST-mediated neuroblastoma tumor formation and growth in vitro and in vivo.

## Discussion

Neuroblastoma is the most common pediatric extracranial solid tumor that originates from embryonal neural crest cells and accounts for 8–10% of all childhood tumors [32]. RA-mediated neuronal differentiation is the most successful therapeutic method for high-risk neuroblastoma [33–35]. However, few neuroblastoma patients exhibit an effective response to RA treatment. Moreover, RA treatment can lead to several side-effects, such as antiproliferative, prodifferentiation, and proapoptotic phenomena [36–39].

Among the several transcriptional factors that are involved in neuroblastoma formation [40], REST plays an important role in the regulation of neural differentiation and tumorigenesis [41–43]. During neural stem cell differentiation, the expression level of REST gradually decreases and is maintained at low levels [9, 12]. However, REST behaves as an oncogene, showing elevated expression in neural tumors. High REST expression has been detected more frequently in neuroblastoma tissues than in normal brain tissues [6, 11, 12, 44, 45]. Moreover, neuroblastoma patients with higher clinical stages are significantly associated with high REST expression [46], suggesting that REST protein level is a critical factor for impaired neuronal differentiation leading to neuroblastoma. Thus, identifying a protein stabilizer of REST and elucidating its molecular mechanism in altering its protein level in neuroblastoma is a top priority. Because DUBs are primarily involved in the regulation of the ubiquitination status of their substrates by reversing ubiquitin-induced impacts on their target proteins, we have attempted to systematically screen genome-wide DUBs that selectively regulate REST protein levels.

In this study, we performed a genome-scale CRISPR/Cas9-based screening for DUBs that regulate REST protein levels. This screening system consisted of cell lines with 50 USP-subfamily genes that were individually knocked out. We have identified USP3 as a potential DUB that increases the REST protein level (Fig. 1). Our screening method based on CRISPR/Cas9-mediated loss-of-function is more reliable and robust than RNAi or shRNA-based screening because the gene disruption generated by CRISPR/Cas9 is permanent [20, 47–51]. Given that USP3 is involved in increasing MYCN and snail protein levels, which are key transcriptional factors that regulate neuronal tumors [52, 53], in this study we explore the post-translational role of USP3 on REST protein during neuroblastoma tumorigenesis.

USP3 plays a key role in regulating diverse biological functions in cancers. For example, the expression of USP3 is highly elevated in gastric cancer, promoting the invasion and migration of gastric cancer cells [54]. In colorectal cancer, loss of USP3 is significantly associated with a poor prognosis and distal metastasis [55]. Recently, we have identified USP3 as a deubiquitinase for CDC25A, regulating its protein turnover and subsequently promoting cell cycle progression and tumorigenesis [20]. USP3 has been upregulated in glioblastoma multiforme (GBM), a lethal malignant brain tumor in adults. Silencing of USP3 blocks the epithelial-mesenchymal transition resulting in drastic inhibition of migration, invasion, and tumor growth in GBM cells [52]. These finding indicate that USP3 may be a potential target for a variety of anti-cancer therapies. Despite considerable study of USP3 regulation in a variety of biological behaviors in several malignancies, the underlying molecular mechanism of USP3 regulating neuroblastoma differentiation and tumorigenesis remains elusive.

DUBs play a crucial role in protein stabilization of their substrate by rescuing it from ubiquitin-mediated protein degradation [17, 20, 47, 48, 50, 56]. A growing body of evidence suggests that DUBs play a significant role in neuroblastoma [1, 17, 18]. HAUSP (USP7) interacts with and stabilizes several target proteins such as N-Myc, c-Myc, and p53 by its deubiquitinating activity in neuroblastoma [17, 57, 58]. The low expression of USP24 has been associated with poor survival in neuroblastoma. USP24 stabilizes collapsing response mediator protein 2, a key regulator of axon growth and neuronal polarity. Deletion of USP24 shows significant defects in spindle formation and cell division. Moreover, USP24 was found to be frequently deleted in neuroblastoma, providing a clue that USP24 may be a critical factor for neuroblastoma pathogenesis [59]. ALYREF-mediated upregulation of USP3 expression prevented MYCN protein degradation and resulted in increased neuroblastoma cell viability in vitro and in vivo [53].

In this study, we demonstrated that USP3 is a bona fide DUB that interacts with REST and stabilizes its protein levels (Fig. 2). Moreover, USP3 regulates REST protein stabilization through the ubiquitin proteasomal pathway (Fig. 3). As a functional consequence of the interaction between USP3 and REST, the half-life of REST protein was extended by the deubiquitinating activity of USP3 (Fig. 3). Previously, Nagy et al. showed that USP3 is highly expressed in neuroblastoma patient cohorts and also associated with poor prognosis [53]. Additionally, it was shown that USP3 deubiquitinates MYCN, an oncoprotein observed in most neuroblastoma patients and considered as an indicator of tumor aggressiveness [60, 61]. Moreover, the expression of REST was elevated in a majority of neuroblastoma [12]. Thus, to elucidate the regulatory mechanism of REST degradation by USP3 in neuroblastoma, we performed an *in silico* analysis of the entire USP gene expression in neuroblastoma. USP3 is one of the top candidates for high expression in neuroblastoma. However, USP44, which emerged as a top candidate (Fig. 4C), is likely to be associated with neuroblastoma development. Based on the computational analysis, researchers from the Mayo clinic predicts that USP44 showing high expression may play a critical role in neuroblastoma patients [59]. Furthermore, a positive correlation between USP3 and REST expression was observed in neuroblastoma (Fig. 4).

The final goal of this study was to elucidate the role of USP3 on REST protein degradation and its influence on self-renewal and differentiation of neuroblastoma. USP3-mediated stabilization of REST contributed to the maintenance of the self-renewal ability in neuroblastoma (Fig. 5). The expression of USP3 is reduced upon RA-induced differentiation along with REST expression. Interestingly, the reduction in USP3 protein level is initiated much earlier than in REST protein, suggesting that down-regulation of USP3 might induce rapid differentiation of neuroblastoma by decreasing its substrate REST protein levels. Furthermore, silencing of USP3 triggered REST protein degradation that promoted neuroblastoma differentiation (Fig. 6). Generally, retinoids have been used in diverse ways as a therapeutic agent in lung cancer, breast cancer, and acute myeloid leukemia [62]. Based on our observation of the silencing of USP3 promoting RA-induced differentiation, we envision that targeting USP3 along with retinoids is likely to enhance retinoid therapy in several cancer types. Finally, USP3 gene knockout using a CRISPR/Cas9 system in neuroblastoma cell lines resulted in reduced cell viability, anchorage-independent growth, migration, and invasion (Fig. 7). Additionally, the loss of USP3 led to a decrease in tumor size in a mouse xenograft model, whereas the tumor size was increased in mice injected with USP3-depleted cells reconstituted with either USP3 or REST (Fig. 7).

## Conclusion

In summary, our results suggest that the high expression of USP3 and REST may impair neuroblastoma differentiation, where neural crest cells lose the ability to differentiate into mature neurons. However, a loss of USP3 eventually reduces the REST protein level and promotes efficient neuroblastoma differentiation. Thus, we envision that USP3 could be a potential alternative therapeutic target for neuroblastoma treatment.

## Electronic supplementary material

Below is the link to the electronic supplementary material.

Supplementary Material 1

Supplementary Material 2

## Data Availability

The datasets were analyzed using R2: genomics Analysis and Visualization Platform.

## Abbreviations

CCLE: Cancer Cell Line Encyclopedia
CRISPR: Clustered regularly interspaced short palindromic repeats
Cas9: CRISPR-associated protein 9
DUBs: Deubiquitinating enzymes
NSG: NOD scidγ
RA: Retinoic acid
REST: Restrictive element-1 silencing transcriptional factor
sgRNA: Single guide RNA
shRNA: Short hairpin RNA
TH: Tyrosine hydroxylase
TUJ1: Tubulin beta III
USPs: Ubiquitin-specific proteases.

## References

[1] Gu Y, Lv F, Xue M, Chen K (2018). The deubiquitinating enzyme UCHL1 is a favorable prognostic marker in neuroblastoma as it promotes neuronal differentiation. J experimental Clin cancer research: CR.

[2] Maris JM (2010). Recent advances in neuroblastoma. N Engl J Med.

[3] Mei Y, Wang Z, Zhang L, Zhang Y (2012). Regulation of neuroblastoma differentiation by forkhead transcription factors FOXO1/3/4 through the receptor tyrosine kinase PDGFRA. Proc Natl Acad Sci USA.

[4] Gao Z, Ure K, Ding P, Nashaat M (2011). The master negative regulator REST/NRSF controls adult neurogenesis by restraining the neurogenic program in quiescent stem cells. J neuroscience: official J Soc Neurosci.

[5] Lunyak VV, Rosenfeld MG (2005). No rest for REST: REST/NRSF regulation of neurogenesis. Cell.

[6] Singh SK, Kagalwala MN, Parker-Thornburg J, Adams H, Majumder S (2008). REST maintains self-renewal and pluripotency of embryonic stem cells. Nature.

[7] Lunyak VV, Burgess R, Prefontaine GG, Nelson C (2002). Corepressor-dependent silencing of chromosomal regions encoding neuronal genes. Sci (New York N Y).

[8] Nechiporuk T, McGann J, Mullendorff K, Hsieh J (2016). The REST remodeling complex protects genomic integrity during embryonic neurogenesis. eLife.

[9] Huang Z, Wu Q, Guryanova OA, Cheng L (2011). Deubiquitylase HAUSP stabilizes REST and promotes maintenance of neural progenitor cells. Nat Cell Biol.

[10] Hwang JY, Zukin RS (2018). REST, a master transcriptional regulator in neurodegenerative disease. Curr Opin Neurobiol.

[11] Su X, Gopalakrishnan V, Stearns D, Aldape K (2006). Abnormal expression of REST/NRSF and myc in neural stem/progenitor cells causes cerebellar tumors by blocking neuronal differentiation. Mol Cell Biol.

[12] Singh A, Rokes C, Gireud M, Fletcher S (2011). Retinoic acid induces REST degradation and neuronal differentiation by modulating the expression of SCF(β-TRCP) in neuroblastoma cells. Cancer.

[13] Conti L, Crisafulli L, Caldera V, Tortoreto M (2012). REST controls self-renewal and tumorigenic competence of human glioblastoma cells. PLoS ONE.

[14] Westbrook TF, Hu G, Ang XL, Mulligan P (2008). SCFbeta-TRCP controls oncogenic transformation and neural differentiation through REST degradation. Nature.

[15] Suresh B, Lee J, Kim H, Ramakrishna S (2016). Regulation of pluripotency and differentiation by deubiquitinating enzymes. Cell Death Differ.

[16] Sarodaya N, Karapurkar J, Kim KS, Hong SH, Ramakrishna S. The Role of Deubiquitinating Enzymes in Hematopoiesis and Hematological Malignancies. *Cancers* 2020, *12*. 10.3390/cancers12051103 PMC7281754 32354135

[17] Tavana O, Li D, Dai C, Lopez G (2016). HAUSP deubiquitinates and stabilizes N-Myc in neuroblastoma. Nat Med.

[18] Kobayashi T, Masoumi KC, Massoumi R (2015). Deubiquitinating activity of CYLD is impaired by SUMOylation in neuroblastoma cells. Oncogene.

[19] Yu Y, Zhao Y, Fan Y, Chen Z (2019). Inhibition of ubiquitin-specific protease 14 suppresses cell proliferation and synergizes with Chemotherapeutic Agents in Neuroblastoma. Mol Cancer Ther.

[20] Das S, Chandrasekaran AP, Suresh B, Haq S (2020). Genome-scale screening of deubiquitinase subfamily identifies USP3 as a stabilizer of Cdc25A regulating cell cycle in cancer. Cell Death Differ.

[21] Ramakrishna S, Cho SW, Kim S, Song M (2014). Surrogate reporter-based enrichment of cells containing RNA-guided Cas9 nuclease-induced mutations. Nat Commun.

[22] Gopalappa R, Suresh B, Ramakrishna S, Kim HH (2018). Paired D10A Cas9 nickases are sometimes more efficient than individual nucleases for gene disruption. Nucleic Acids Res.

[23] Livak KJ, Schmittgen TD (2001). Analysis of relative gene expression data using real-time quantitative PCR and the 2(-Delta Delta C(T)) method. Methods (San Diego Calif).

[24] Nguyen TV, Li J, Lu CJ, Mamrosh JL (2017). p97/VCP promotes degradation of CRBN substrate glutamine synthetase and neosubstrates. Proc Natl Acad Sci USA.

[25] Nguyen TV. USP15 antagonizes CRL4(CRBN)-mediated ubiquitylation of glutamine synthetase and neosubstrates. Proc Natl Acad Sci USA 2021, *118*. 10.1073/pnas.2111391118 PMC8501880 34583995

[26] Zhou A, Lin K, Zhang S, Chen Y (2016). Nuclear GSK3β promotes tumorigenesis by phosphorylating KDM1A and inducing its deubiquitylation by USP22. Nat Cell Biol.

[27] Hu Y, Smyth GK (2009). ELDA: extreme limiting dilution analysis for comparing depleted and enriched populations in stem cell and other assays. J Immunol Methods.

[28] Miller HE, Bishop AJR (2021). Correlation AnalyzeR: functional predictions from gene co-expression correlations. BMC Bioinformatics.

[29] Gentles AJ, Newman AM, Liu CL, Bratman SV (2015). The prognostic landscape of genes and infiltrating immune cells across human cancers. Nat Med.

[30] Lee JK, Chang N, Yoon Y, Yang H (2016). USP1 targeting impedes GBM growth by inhibiting stem cell maintenance and radioresistance. Neurooncology.

[31] Faronato M, Patel V, Darling S, Dearden L (2013). The deubiquitylase USP15 stabilizes newly synthesized REST and rescues its expression at mitotic exit. Cell cycle (Georgetown Tex).

[32] Castleberry RP (1997). Neuroblastoma. Eur J cancer (Oxford England: 1990).

[33] Reynolds CP, Lemons RS (2001). Retinoid therapy of childhood cancer. Hematol Oncol Clin N Am.

[34] Matthay KK, Villablanca JG, Seeger RC, Stram DO (1999). Treatment of high-risk neuroblastoma with intensive chemotherapy, radiotherapy, autologous bone marrow transplantation, and 13-cis-retinoic acid. Children’s Cancer Group. N Engl J Med.

[35] Reynolds CP, Kane DJ, Einhorn PA, Matthay KK (1991). Response of neuroblastoma to retinoic acid in vitro and in vivo. Prog Clin Biol Res.

[36] Zimmerman MW, Durbin AD, He S, Oppel F (2021). Retinoic acid rewires the adrenergic core regulatory circuitry of childhood neuroblastoma. Sci Adv.

[37] Matsuo T, Thiele CJ (1998). p27Kip1: a key mediator of retinoic acid induced growth arrest in the SMS-KCNR human neuroblastoma cell line. Oncogene.

[38] Niles RM (2004). Signaling pathways in retinoid chemoprevention and treatment of cancer. Mutat Res.

[39] Teppola H, Sarkanen JR, Jalonen TO, Linne ML (2016). Morphological differentiation towards neuronal phenotype of SH-SY5Y Neuroblastoma cells by Estradiol, retinoic acid and cholesterol. Neurochem Res.

[40] Boeva V, Louis-Brennetot C, Peltier A, Durand S (2017). Heterogeneity of neuroblastoma cell identity defined by transcriptional circuitries. Nat Genet.

[41] Hirabayashi Y, Gotoh Y (2010). Epigenetic control of neural precursor cell fate during development. Nat Rev Neurosci.

[42] Soldati C, Bithell A, Johnston C, Wong KY (2012). Repressor element 1 silencing transcription factor couples loss of pluripotency with neural induction and neural differentiation. Stem Cells.

[43] Negrini S, Prada I, D’Alessandro R, Meldolesi J (2013). REST: an oncogene or a tumor suppressor?. Trends Cell Biol.

[44] Kamal MM, Sathyan P, Singh SK, Zinn PO (2012). REST regulates oncogenic properties of glioblastoma stem cells. Stem Cells.

[45] Westbrook TF, Martin ES, Schlabach MR, Leng Y (2005). A genetic screen for candidate tumor suppressors identifies REST. Cell.

[46] Liang J, Tong P, Zhao W, Li Y (2014). The REST gene signature predicts drug sensitivity in neuroblastoma cell lines and is significantly associated with neuroblastoma tumor stage. Int J Mol Sci.

[47] Kaushal K, Kim EJ, Tyagi A, Karapurkar JK (2022). Genome-wide screening for deubiquitinase subfamily identifies ubiquitin-specific protease 49 as a novel regulator of odontogenesis. Cell Death Differ.

[48] Tyagi A, Kaushal K, Chandrasekaran AP, Sarodaya N (2022). CRISPR/Cas9-based genome-wide screening for deubiquitinase subfamily identifies USP1 regulating MAST1-driven cisplatin-resistance in cancer cells. Theranostics.

[49] Chandrasekaran AP, Tyagi A, Poondla N, Sarodaya N (2022). Dual role of deubiquitinating enzyme USP19 regulates mitotic progression and tumorigenesis by stabilizing survivin. Mol therapy: J Am Soc Gene Therapy.

[50] Haq S, Sarodaya N, Karapurkar JK, Suresh B (2022). CYLD destabilizes NoxO1 protein by promoting ubiquitination and regulates prostate cancer progression. Cancer Lett.

[51] Sarodaya N, Tyagi A, Kim HJ, Colaco JC et al. Deubiquitinase USP19 enhances phenylalanine hydroxylase protein stability and its enzymatic activity. Cell Biol Toxicol 2022. 10.1007/s10565-022-09719-z 35449354

[52] Fan L, Chen Z, Wu X, Cai X (2019). Ubiquitin-specific protease 3 promotes Glioblastoma Cell Invasion and epithelial-mesenchymal transition via stabilizing snail. Mol cancer research: MCR.

[53] Nagy Z, Seneviratne JA, Kanikevich M, Chang W (2021). An ALYREF-MYCN coactivator complex drives neuroblastoma tumorigenesis through effects on USP3 and MYCN stability. Nat Commun.

[54] Wu X, Liu M, Zhu H, Wang J (2019). Ubiquitin-specific protease 3 promotes cell migration and invasion by interacting with and deubiquitinating SUZ12 in gastric cancer. J experimental Clin cancer research: CR.

[55] Wang Z, Yang J, Di J, Cui M (2017). Downregulated USP3 mRNA functions as a competitive endogenous RNA of SMAD4 by sponging miR-224 and promotes metastasis in colorectal cancer. Sci Rep.

[56] Chandrasekaran AP, Tyagi A, Poondla N, Sarodaya N et al. Dual role of deubiquitinating enzyme USP19 regulates mitotic progression and tumorigenesis by stabilizing survivin. Mol therapy: J Am Soc Gene Therapy 2022. 10.1016/j.ymthe.2022.07.019 PMC9637645 35918893

[57] Fan YH, Cheng J, Vasudevan SA, Dou J (2013). USP7 inhibitor P22077 inhibits neuroblastoma growth via inducing p53-mediated apoptosis. Cell Death Dis.

[58] Nicklas S, Hillje AL, Okawa S, Rudolph IM (2019). A complex of the ubiquitin ligase TRIM32 and the deubiquitinase USP7 balances the level of c-Myc ubiquitination and thereby determines neural stem cell fate specification. Cell Death Differ.

[59] Bedekovics T, Hussain S, Zhang Y, Ali A (2021). USP24 is a Cancer-Associated Ubiquitin Hydrolase, Novel Tumor suppressor, and chromosome instability gene deleted in Neuroblastoma. Cancer Res.

[60] Brodeur GM, Maris JM, Yamashiro DJ, Hogarty MD, White PS (1997). Biology and genetics of human neuroblastomas. J Pediatr Hematol Oncol.

[61] Schwab M, Alitalo K, Klempnauer KH, Varmus HE (1983). Amplified DNA with limited homology to myc cellular oncogene is shared by human neuroblastoma cell lines and a neuroblastoma tumour. Nature.

[62] Martino OD, Welch JS. Retinoic Acid Receptors in Acute Myeloid Leukemia Therapy. *Cancers* 2019, *11*. 10.3390/cancers11121915 PMC6966485 31805753

