# Supplementary material for: CRISPR/Cas9-based genome-wide screening of the deubiquitinase subfamily identifies USP3 as a protein stabilizer of REST blocking neuronal differentiation and promotes neuroblastoma tumorigenesis
Source: J Exp Clin Cancer Res. 2023 May 12;42:121. doi: 10.1186/s13046-023-02694-1 (PMC10176696; doi:10.1186/s13046-023-02694-1)
Supplement: Supplementary file 1 — Supplementary Material 1 [file 13046_2023_2694_MOESM1_ESM.docx]

**Supplementary Information**

**CRISPR/Cas9-based genome-wide screening of the deubiquitinase subfamily identifies USP3 as a protein stabilizer of REST blocking neuronal differentiation and promotes neuroblastoma tumorigenesis**

Janardhan Keshav Karapurkar^1,^ **^†^**, Min-Seong Kim^1,^ **^†^**, Jencia Carminha Colaco^1^, Bharathi Suresh^1^, Neha Sarodaya^1^, Dong-Ho Kim^1^, Chang-Hwan Park^1,2^, Seok-Ho Hong^3^, Kye-Seong Kim^1,2^***** and Suresh Ramakrishna^1,2^*****

^1^Graduate School of Biomedical Science and Engineering, Hanyang University, Seoul, 04763, South Korea

^2^College of Medicine, Hanyang University, Seoul, 04763, South Korea

^3^Department of Internal Medicine, School of Medicine, Kangwon National University, Chuncheon, South Korea

**Running title:** Loss of USP3 blocks neuroblastoma tumorigenesis

**^†^** These authors contributed equally.

***Corresponding Authors Information**

SR (E-mail: [suri28@hanyang.ac.kr](mailto:suri28@hanyang.ac.kr), [suresh.ramakris@gmail.com](mailto:suresh.ramakris@gmail.com))

KS (E-mail: ks66kim@hanyang.ac.kr)

**Supplementary Figures:**

**Supplementary Fig. 1** The cell viability analysis on putative DUBs regulating REST protein level.

**Supplementary Fig. 2** The effect of USP3 and USP3CS on the half-life of endogenous REST in SH-SY5Y cells.

**Supplementary Fig. 3** Correlation between USP3 and REST expression in various cancer

**Supplementary Fig. 4** Correlation between USP3 expression and survival in neuroblastoma patients.

**Supplementary Fig. 5** USP3 knockdown effect on the ADR and MES protein markers in the SK-N-DZ and SK-N-AS cells

**Supplementary Fig. 6** USP3 knockdown effect on the self-renewal protein markers in SK-N-DZ and SK-N-SH cells.

**Supplementary Fig. 7** Depletion of USP3 affects self-renewal and cell proliferation in neuroblastoma.

**Supplementary Fig. 8** Effect of RA induced differentiation on USP3 and REST protein levels in SK-N-SH cell line.

**Supplementary Fig. 9** Depletion of USP3 promotes retinoic acid (RA)-induced neuronal differentiation in SK-N-SH cells.

**Supplementary Fig. 10** Depletion of USP3 promotes retinoic acid (RA)-induced neuronal differentiation in SK-N-DZ cells.

**Supplementary Fig. 11** Screening for USP3 knockout cell line.

**Supplementary Fig. 12** Validation of USP3 and REST expression in USP3-silenced SK-N-SH cells.

**Supplementary Fig. 13 Loss** of USP3 inhibits cell viability, colony formation, cell invasion and cell migration in SK-N-SH neuroblastoma cells.

**Supplementary Fig. 14** Loss of USP3 inhibits cell viability, colony formation, and cell invasion in SK-N-AS neuroblastoma cells.

**Supplementary Fig. 15** Loss of USP3 inhibits cell viability, colony formation, and cell invasion in SK-N-DZ neuroblastoma cells.

.

**Supplementary Tables**

**Supplementary Table S1**. Target sequences used for sgRNA plasmid construction.

**Supplementary Table S2.** Oligonucleotide sequences used to get PCR amplicon for T7E1 assay.

**Supplementary Table S3.** PCR amplicon and cleavage sizes after T7E1 assay.

**Supplementary Table S4.** Oligonucleotide sequence used for qRT-PCR.

**Supplementary Table S5.** PRECOG meta Z-score for USP3 expression across different cancers.

**Supplementary Table S6.** PRECOG meta Z-score for all the DUBs related to USP subfamily in neuroblastoma.

**Supplementary Table S7.** The mRNA scores for USP3 and REST expression derived from the Cancer Cell Line Encyclopedia database in neuroblastoma cell lines.

**Figure Legends**


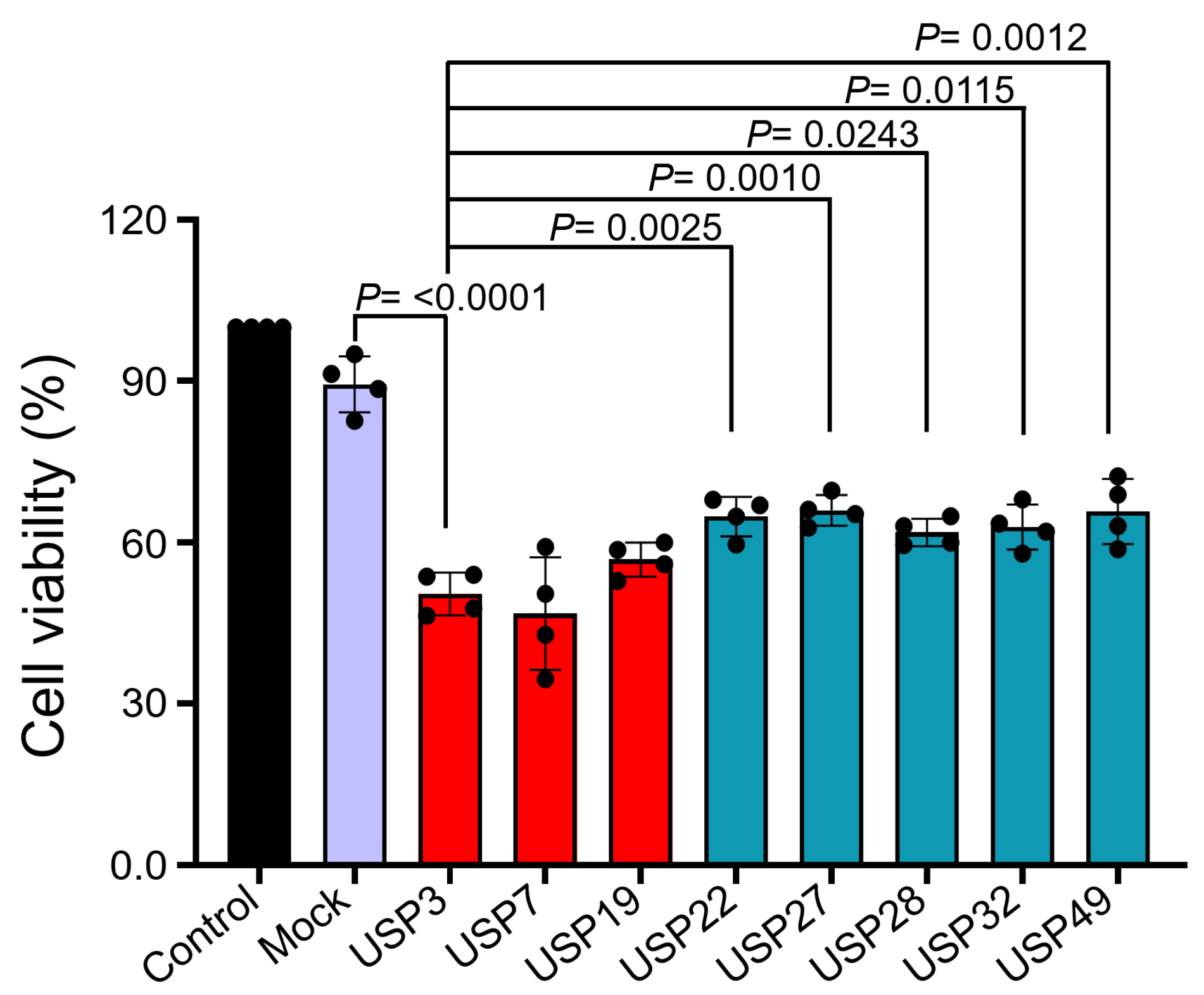


**Supplementary Fig. 1** The cell viability analysis on putative DUBs regulating REST protein level. The cell viability between putative DUBs regulating REST protein and DUBs which are not having any effect on REST protein level was compared using CCK-8 kit. Untransfected cells served as a control and SH-SY5Y cells transfected with scrambled sg-RNA and Cas9 served as a mock control. Data are presented as a mean and standard deviation of three independent experiments (n=3). One-way ANOVA followed by Tukey’s post hoc test was used, and *P* values are indicated.


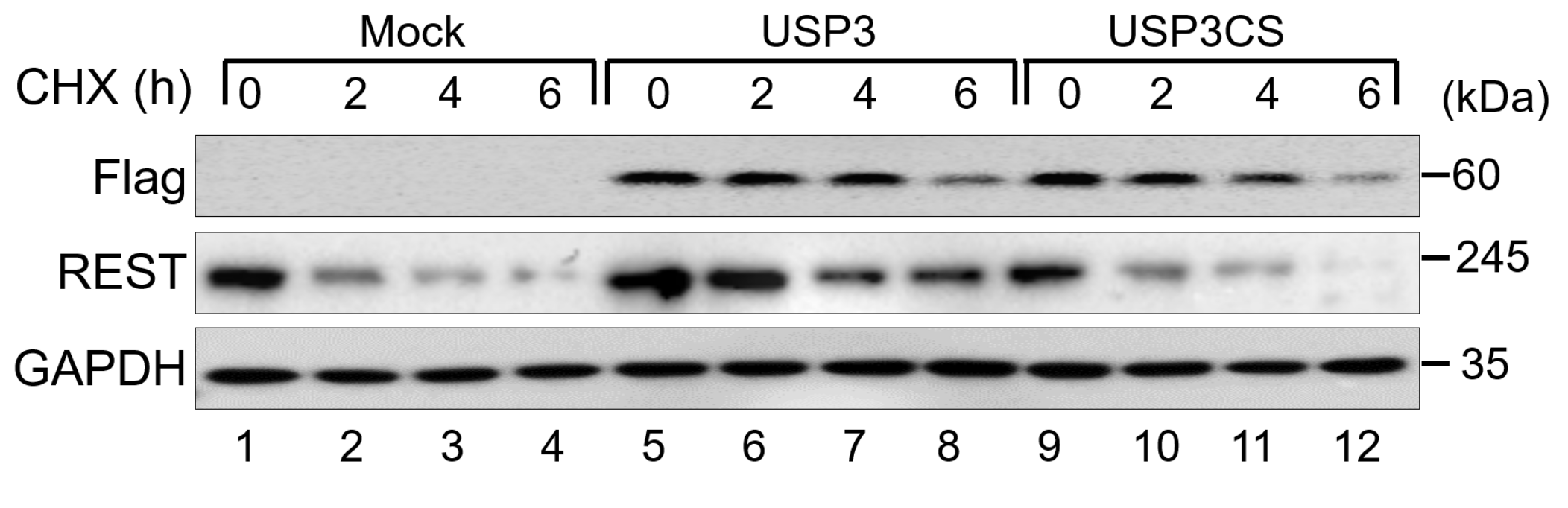


**Supplementary Fig. 2** The effect of USP3 and USP3CS on the half-life of endogenous REST in SH-SY5Y cells. CHX (150 μg/mL) was administered for the indicated time, and the cells were then harvested for western blotting with the indicated antibodies.


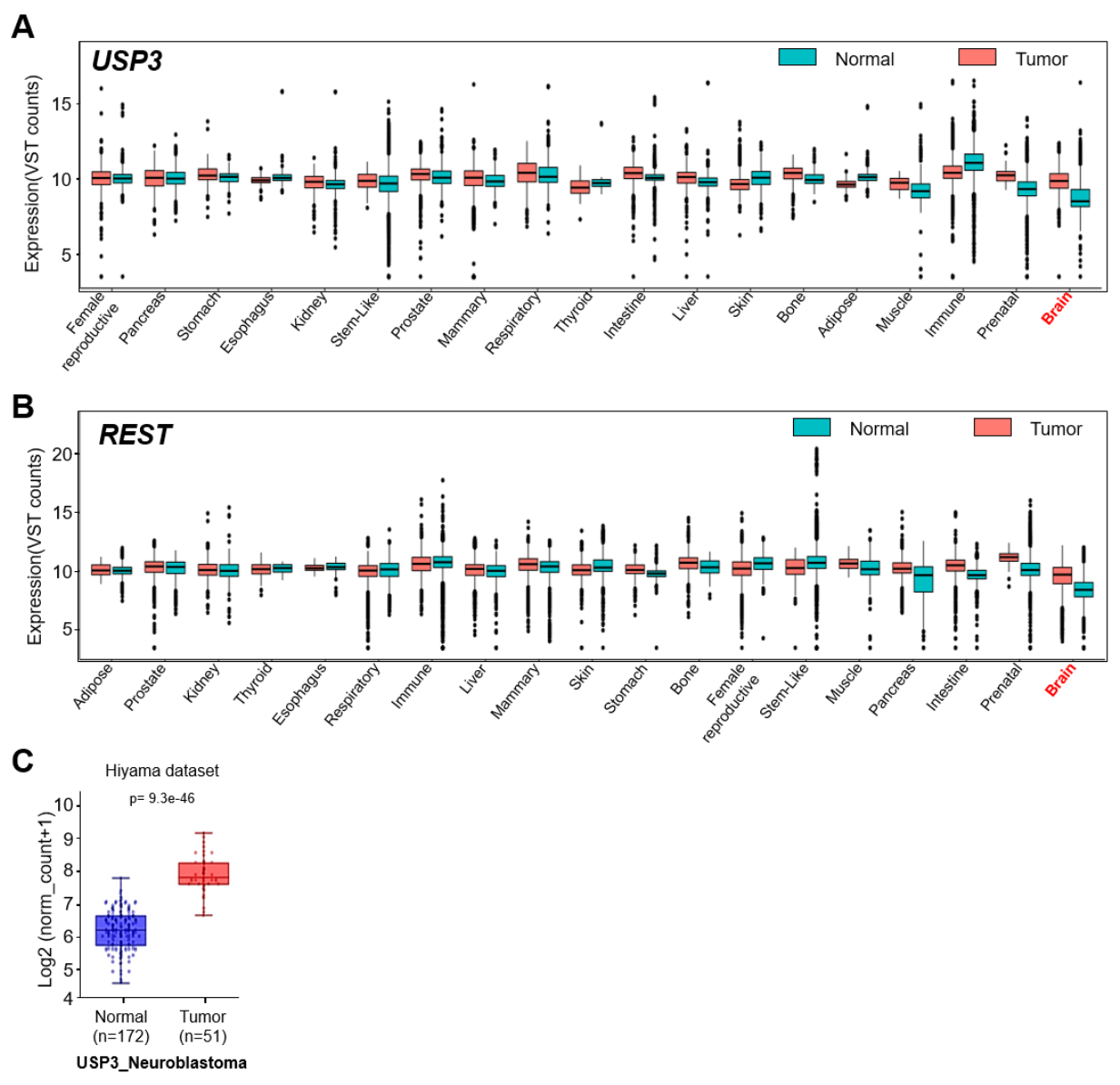


**Supplementary Fig. 3** Correlation between USP3 and REST expression in various cancer. **(A)** Box plot showing difference between USP3 expression in normal and cancer tissues evaluated using Correlation AnalyzeR. Significance was determined by the Wilcoxon rank sum test **** *P<0.0001*. VST stands for variance-stabilizing transform. **(B)** Box plot showing difference between REST expression in normal and cancer tissues evaluated using Correlation AnalyzeR. Significance was determined by the Wilcoxon rank sum test **** *P<0.0001*. VST stands for variance-stabilizing transform **(C)** Box plot showing the difference between USP3 expression in normal (n = 172) and tumor (n = 51) tissues using the Hiyama neuroblastoma dataset: p-value = 9.3 e^-46^. The box plot was generated using R2: genomic Analysis and Visualization Platform (<https://r2.amc.nl>)


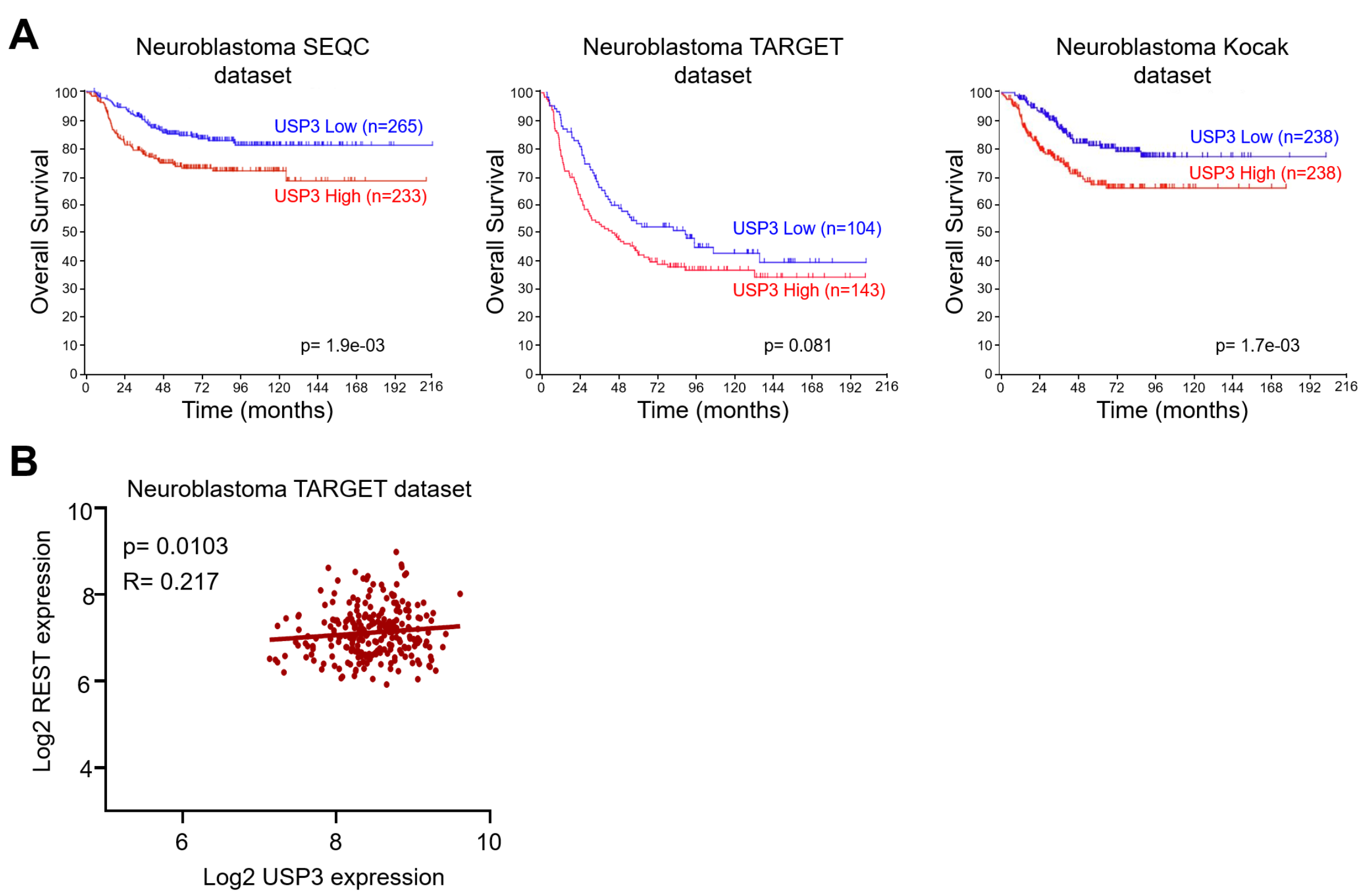


**Supplementary Fig. 4** Correlation between USP3 expression and survival in neuroblastoma patients. **(A)** Kaplan-Meier analysis showing overall survival probability of groups with low USP3 expression and high USP3 expression group in a SEQC (n= 498), TARGET (n=247) and Kocak (n=476) neuroblastoma datasets. High USP3 expression was associated with lower survival rate. The overall survival graphs were generated using R2: genomic Analysis and Visualization Platform (https://r2.amc.nl) and *P*-values are indicated. **(B)** A scatterplot showing the expression between *USP3* and *REST* mRNA levels was calculated from TARGET neuroblastoma dataset. Pearson correlations (r) quantifying the relationship between *USP3* and *REST* is given. The scatterplot between REST and USP3 mRNA expression was generated using R2: genomic Analysis and Visualization Platform (https://r2.amc.nl)


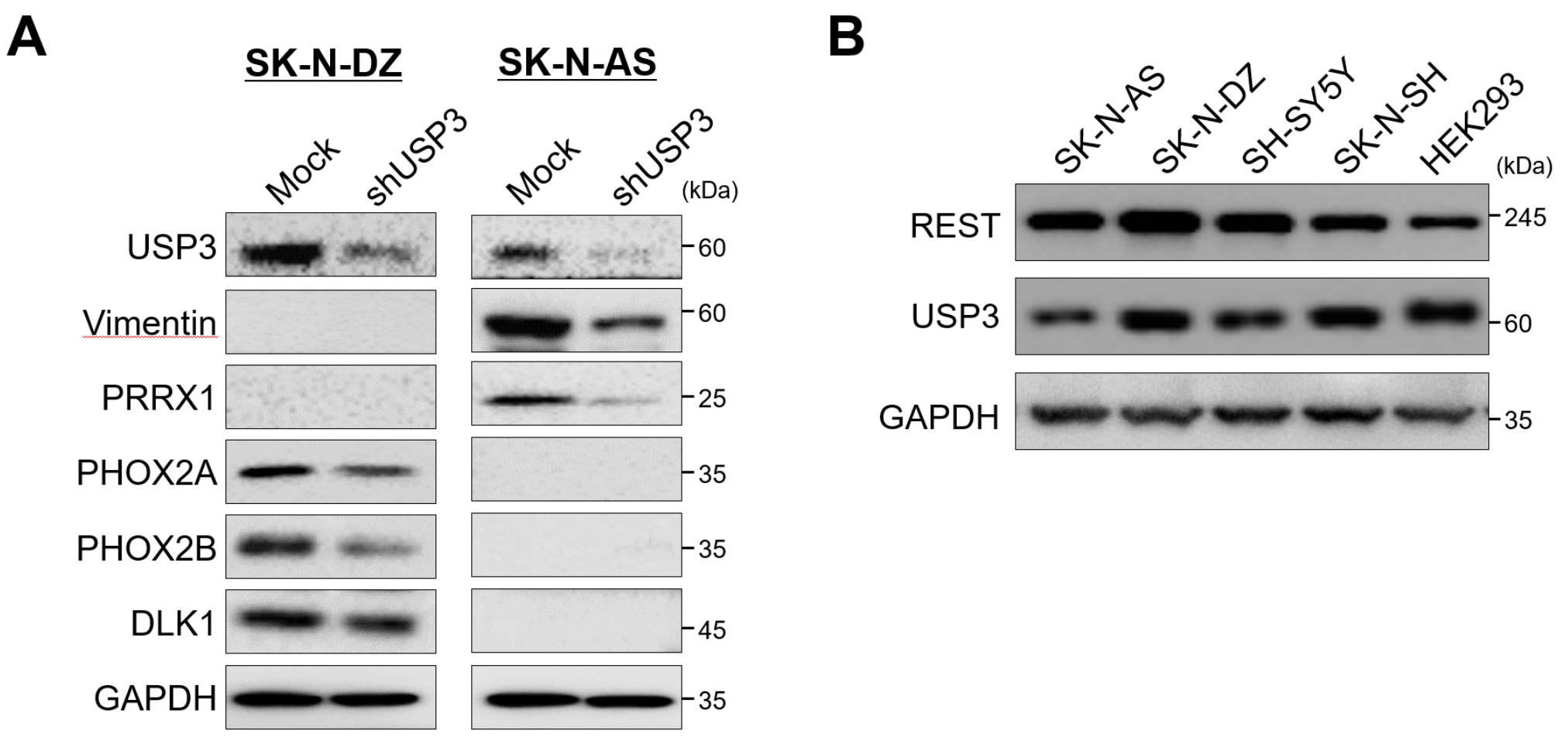


**Supplementary Fig. 5** USP3 knockdown effect on the ADR and MES protein markers in the SK-N-DZ and SK-N-AS cells.**(A)** The effect of shRNA-mediated USP3 knockdown on mesenchymal (Vimentin and PRRX1) and adrenergic-markers (PHOX2A, PHOX2B and DLK1) in SK-N-DZ (ADRN) and SK-N-AS (MES) cell lines were analyzed by western blot analysis. GAPDH was used as a loading control. **(B)** The expression of endogenous USP3 and REST proteins in different neuroblastoma cell lines were assessed by western blot analysis. GAPDH was used as a loading control.


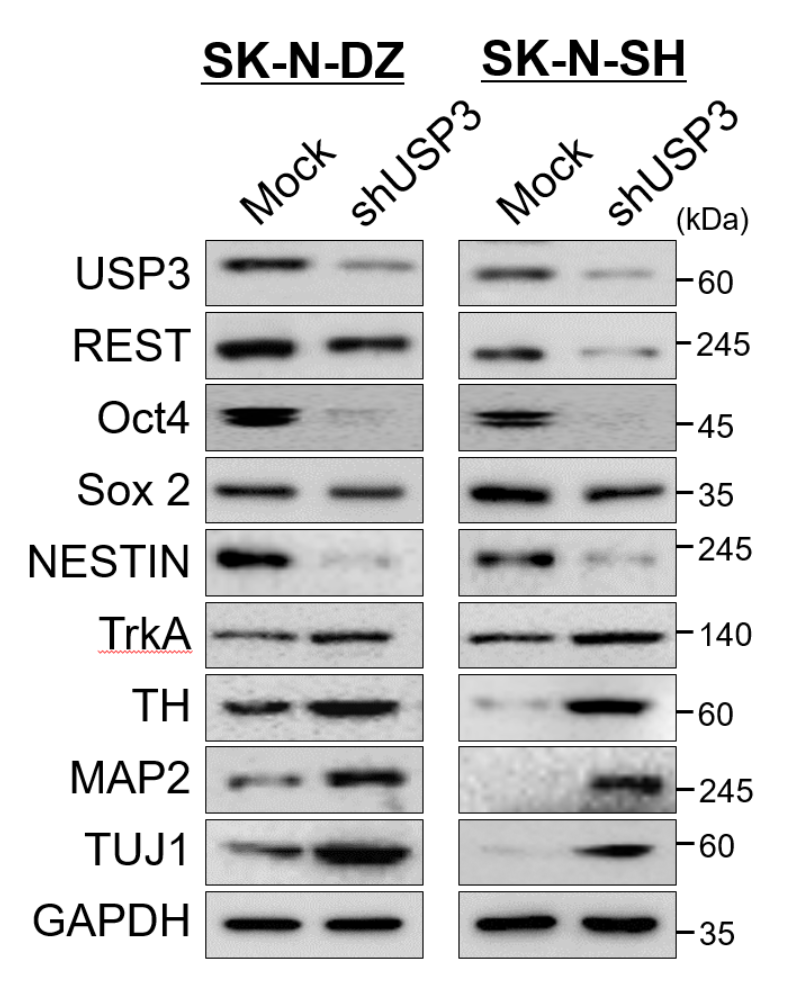


**Supplementary Fig. 6** USP3 knockdown effect on the self-renewal protein markers in SK-N-DZ and SK-N-SH cells was analyzed by western blotting using indicated antibodies. GAPDH was used as the loading control.


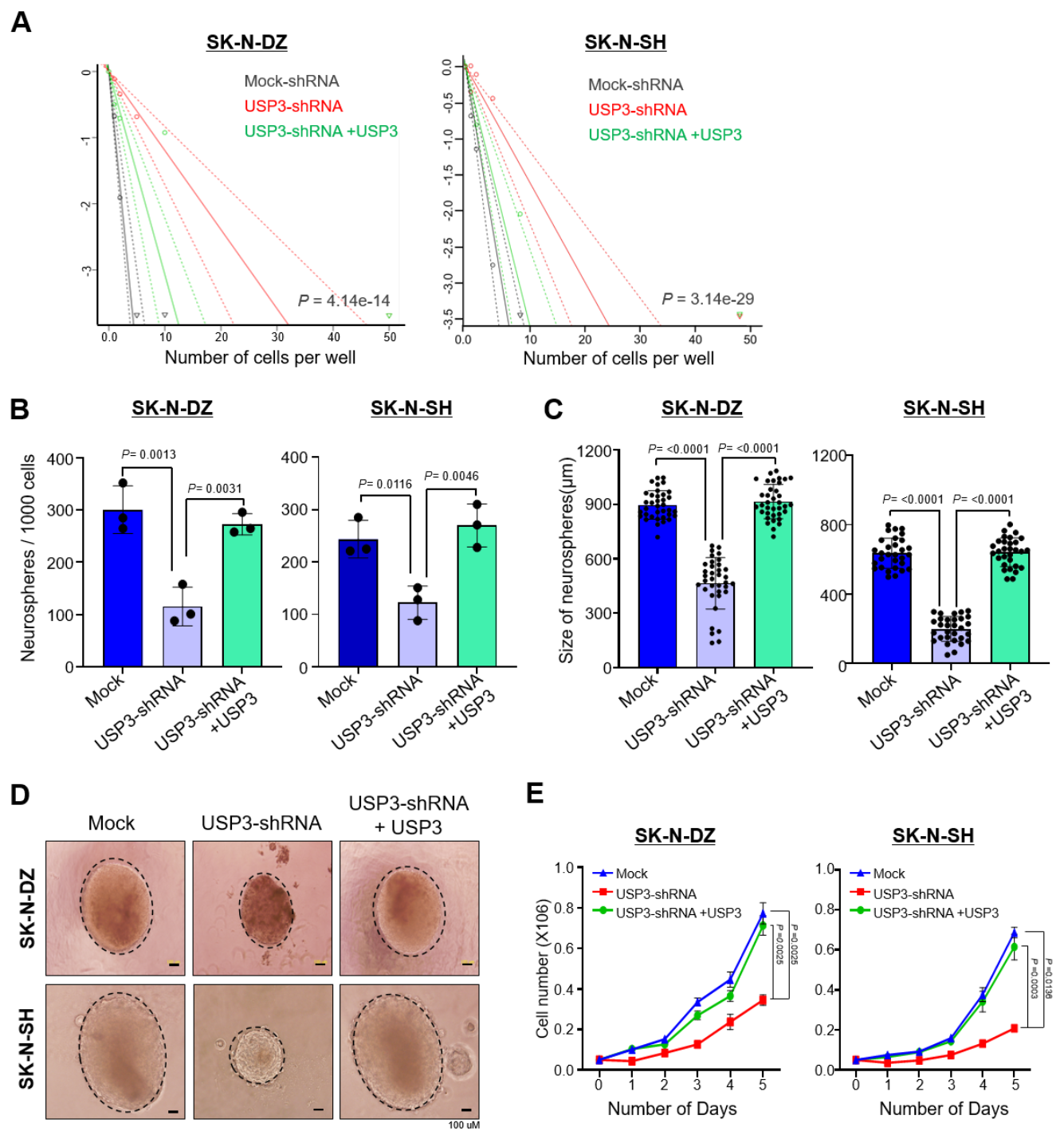


**Supplementary Fig. 7** Depletion of USP3 affects self-renewal and cell proliferation in neuroblastoma. SK-N-DZ and SK-N-SH neuroblastoma cells transiently transfected with scrambled shRNA (Mock) or USP3 shRNA and USP3-depleted cells reconstituted with USP3 were used to perform the following experiments. **(A)** Cells were subjected to *in vitro* limiting dilution assay. The cells were dissociated and seeded in low-attachment 96-well plates at cell densities of 1, 2, 5, 10, and 50 cells/well and cultured for 2 weeks in neurosphere forming media. The frequency of neurosphere forming ability was evaluated using ELDA. Data are presented as the mean and standard deviation of at least three independent experiments. The significance of the difference between the indicated groups were determined by the χ^2^ test, and *P* values are indicated. **(B)** The effect of USP3 on the neurosphere forming ability was evaluated in SK-N-DZ and SK-N-SH cell lines. The cells were dissociated and seeded at density of 2 × 10^4^ and the number of neurospheres were quantified. Data are presented as the mean and standard deviation of three independent experiments (n = 3). One-way ANOVA followed by Tukey’s post hoc test was used, and *P* values are indicated. **(C)** The quantitative analysis of mean size of neurospheres after 2 weeks in neurosphere-forming media. Data are presented as the mean and standard deviation of three independent experiments (n = 3). One-way ANOVA followed by Tukey’s post hoc test was used, and *P* values are indicated. **(D)** Representative bright field microscopy images of the neurosphere formation of neuroblastoma cells expressing control shRNA, USP3 shRNA, and USP3 shRNA+USP3. Scale bar = 100 µM. **(E)** The SK-N-DZ and SK-N-SH cells from were seeded in 6-well plates at density of 0.05 × 10^6^ cells/well. The cells were harvested and counted at indicated time points. Data are presented as the mean and standard deviation of three independent experiments (n = 3). Two-way ANOVA followed by Tukey’s post hoc test was used, and *P* values are indicated.


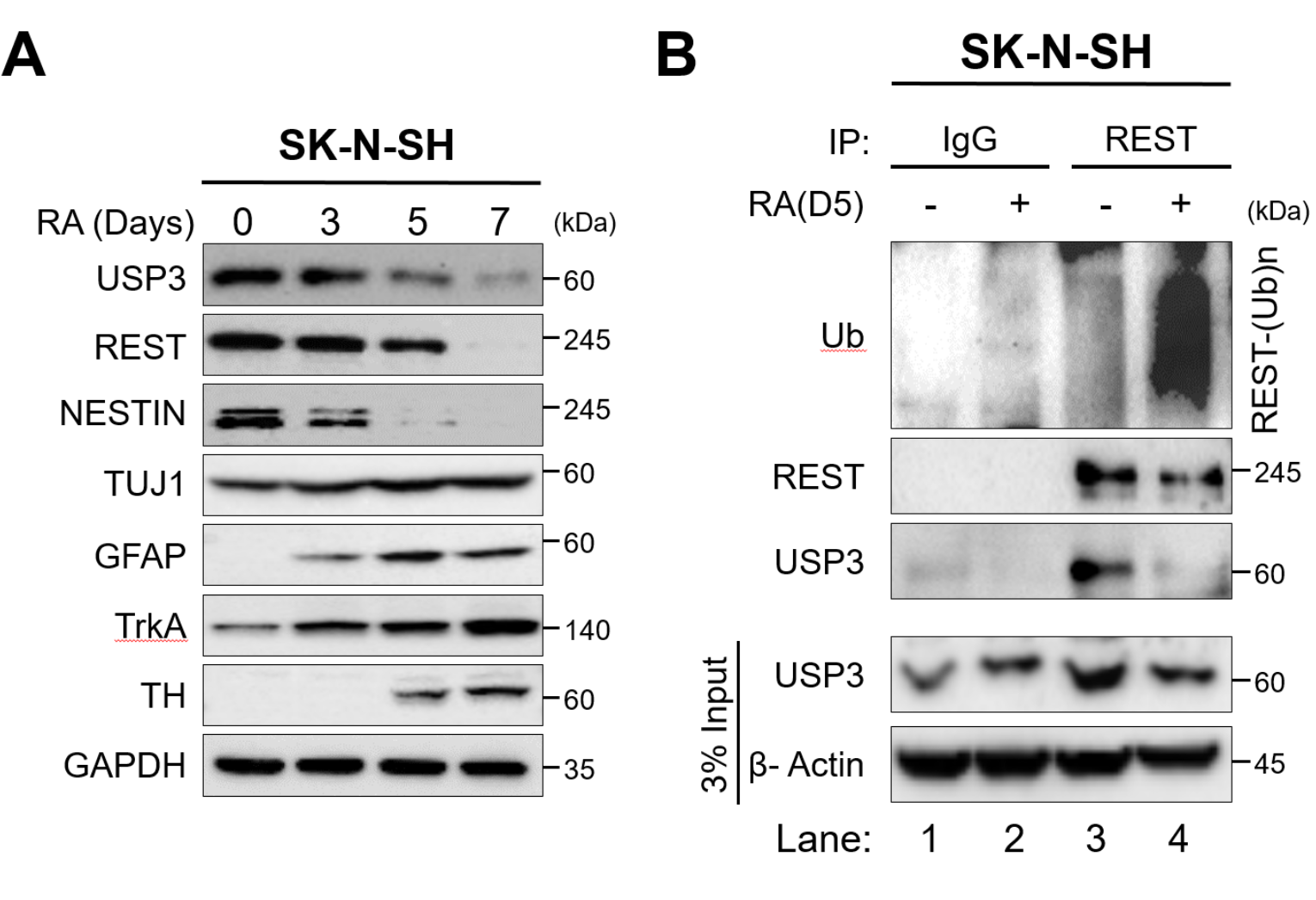


**Supplementary Fig. 8** Effect of RA induced differentiation on USP3 and REST protein levels in SK-N-SH cell line. **(A)** The SK-N-SH cell lines were treated with 10 µM *all trans*-RA to undergo differentiation for the indicated time. The cells were harvested at a given time point and the effect of RA during neuroblastoma differentiation on USP3 and REST protein levels was assessed by western blot analysis. Nestin (neural stem cell marker) and TUJ1, GFAP, TrkA and TH (neuronal marker) were used as controls for differentiation while GAPDH was used as an internal loading control. **(B)** The effect of RA-induced differentiation on REST ubiquitination in SK-N-SH cell line. The SK-N-SH cells were induced to differentiate by treating with 10 µM *all trans-* RA for 5 days, and then treated with MG132 for 6 h before harvest. Cell lysates were immunoprecipitated with anti-REST antibody and immunoblotting with an anti-ubiquitin.


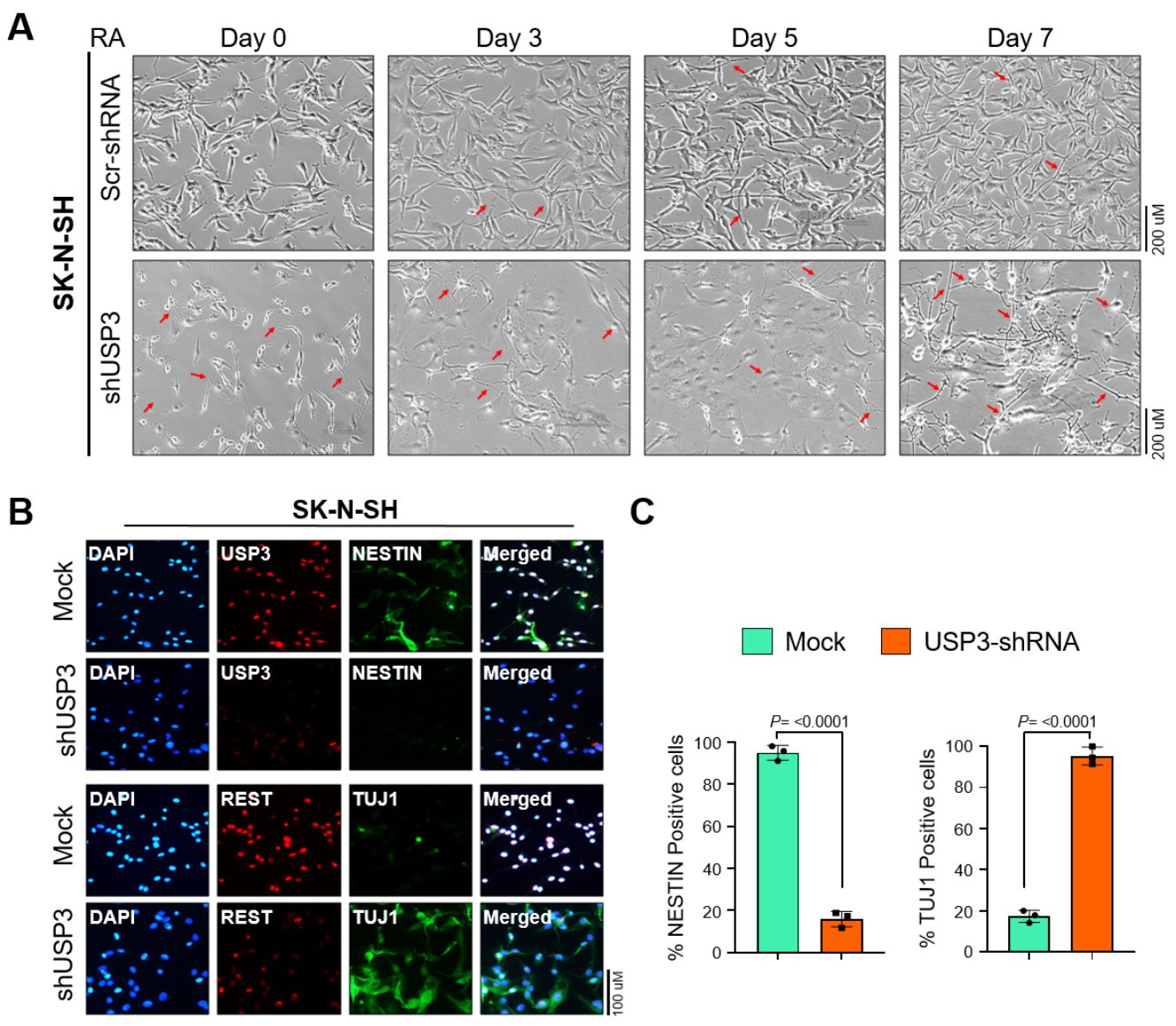


**Supplementary Fig. 9** Depletion of USP3 promotes retinoic acid (RA)-induced neuronal differentiation in SK-N-SH cells **(A)** Representative microscopy images showing depletion of USP3 promotes neurite outgrowth during RA-induced differentiation in neuroblastoma. The SK-N-SH cells were either transfected with scrambled-shRNA (scr-shRNA) or USP3-shRNA and treated with 10 µM *all trans*- RA for indicated days. The morphology of the cells was examined under a bright field microscope. The red arrow indicates neurite extension indicating differentiation. Scale bar = 200 μM **(B)** Immunofluorescence staining on differentiated neuroblastoma cells using USP3, REST, Nestin, and TUJ1 antibodies. Scale bar = 100 μM **(C)** The immunofluorescence staining data from (B) was quantified. The number of TUJ1 and Nestin positive cells were quantified in USP3-depleted cells through immunofluorescence analysis. Data are presented as the mean and standard deviation of three independent experiments. Student’s t-test was used, and *P* values are indicated.


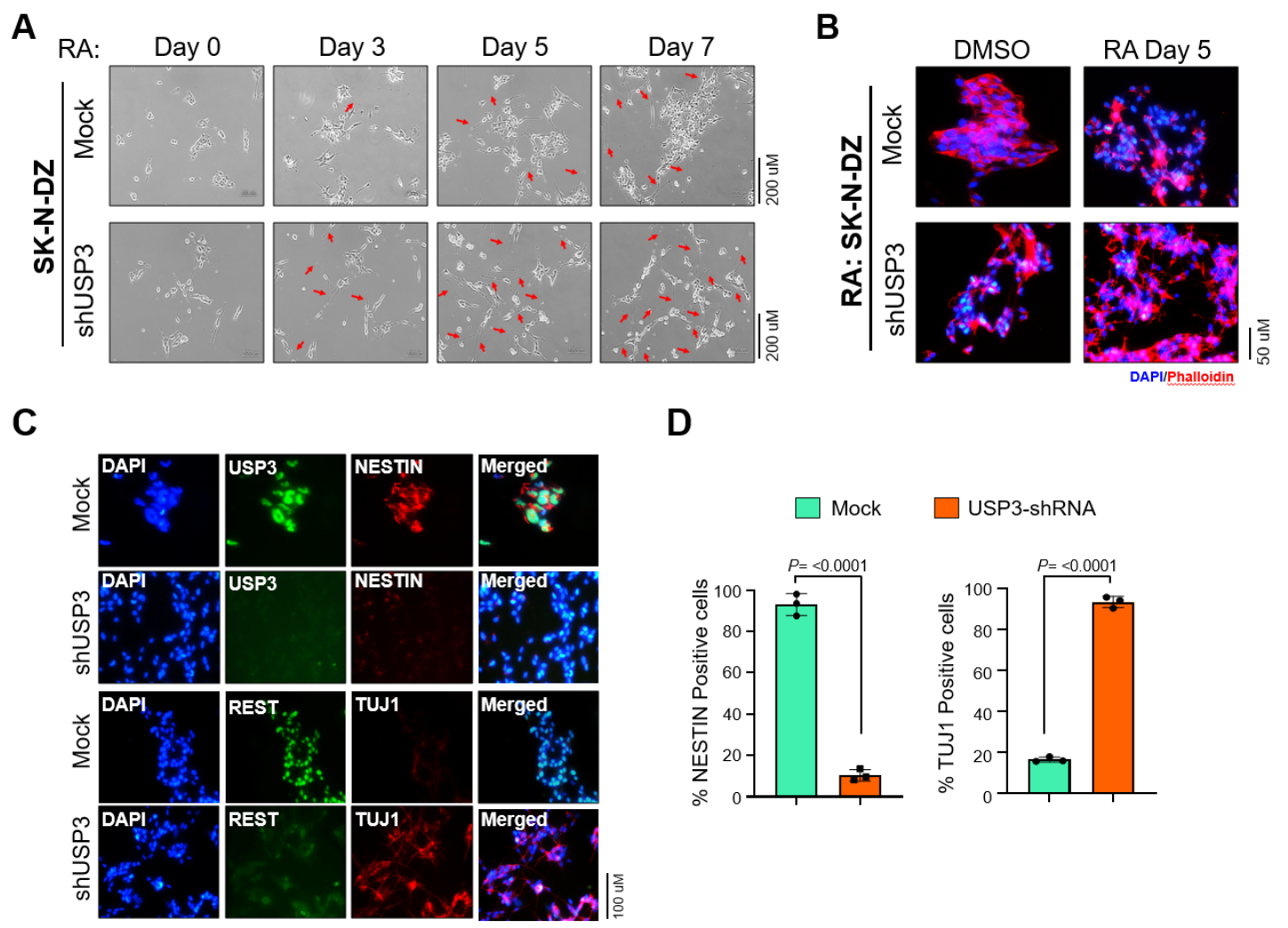


**Supplementary Fig. 10** Depletion of USP3 promotes retinoic acid (RA)-induced neuronal differentiation in SK-N-DZ cells. **(A-B)** The morphology of the cells was examined under (A) a bright field microscope and (B) phalloidin staining showing depletion of USP3 promotes neurite outgrowth during RA-induced differentiation in neuroblastoma cells. Representative microscopy images showing depletion of USP3 promotes neurite outgrowth during RA-induced differentiation in neuroblastoma. The SK-N-DZ cells were either transfected with scrambled-shRNA (scr-shRNA) or USP3-shRNA and treated with 10 µM *all trans*- RA for indicated days. The red arrow indicates neurite extension indicating differentiation. Scale bar = 200 μM **(C)** The effect of USP3 depletion on RA-induced differentiation on SK-N-DZ cells were evaluated by immunofluorescence analysis using indicated antibodies. Immunofluorescence staining on differentiated neuroblastoma cells using USP3, REST, Nestin, and TUJ1 antibodies. Scale bar = 100 μM. **(D)** The immunofluorescence staining data from (C) was quantified. The number of TUJ1 and Nestin positive cells were quantified in USP3-depleted cells through immunofluorescence analysis. Data are presented as the mean and standard deviation of three independent experiments. Student’s t-test was used, and *P* values are indicated.


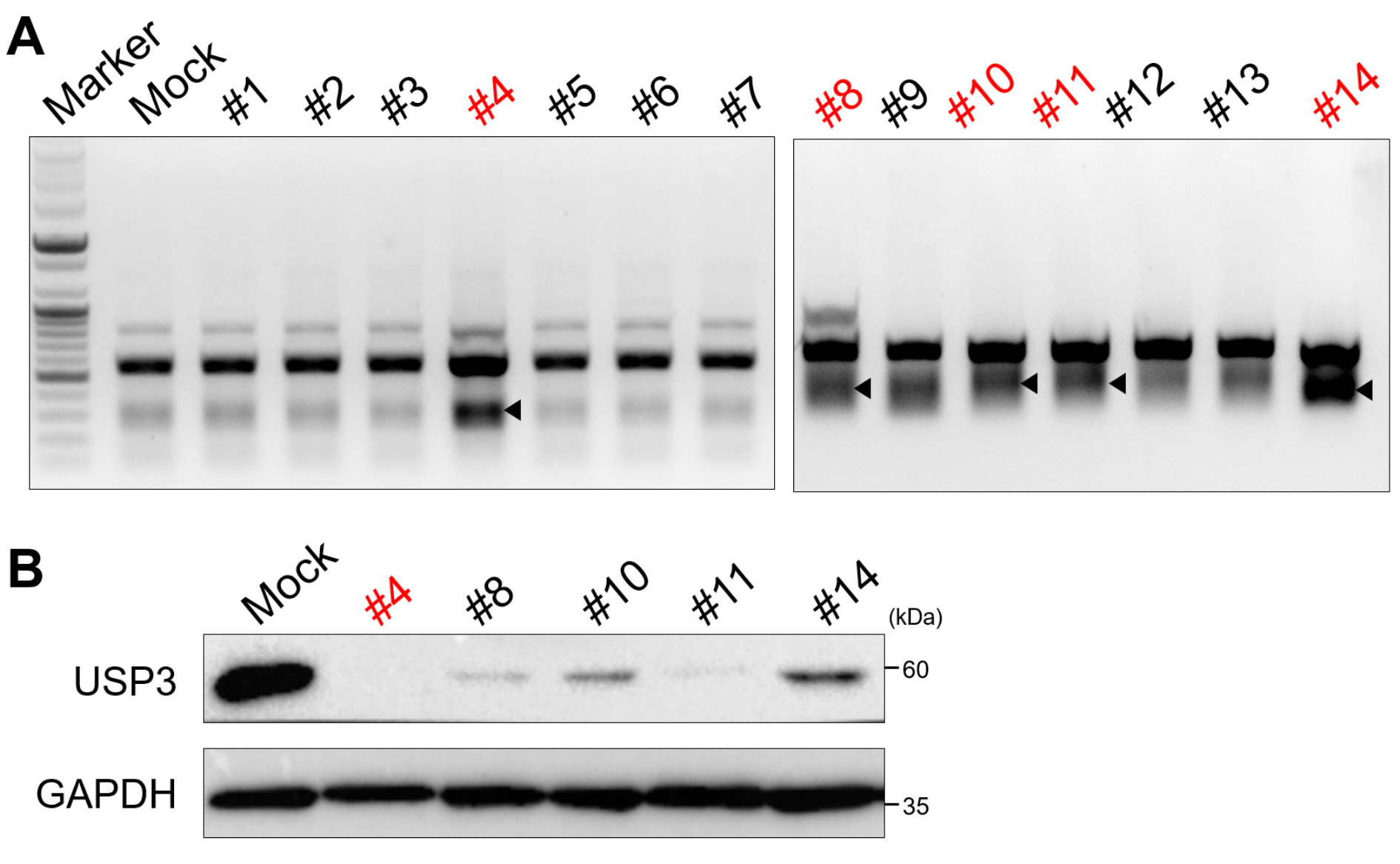


**Supplementary Fig. 11** Screening for USP3 knockout cell line. The SH-SY5Y cells were transfected with Cas9 and sgRNAs targeting USP3 to generated USP3-KO cell line. **(A)** The cells were seeded in 96 well plates and grown to form colony. The SH-SY5Y USP3 knockout cell line was screened by T7E1 assay. The T7E1 positive clones showing cleavage are represented in red color. The arrow head indicates the cleaved bands of the PCR amplicons **(B)** Western blot analysis showing USP3 knockout efficiency in SH-SY5Y cells. The T7E1 negative clone was used as a mock control. GAPDH was used as the internal loading control.


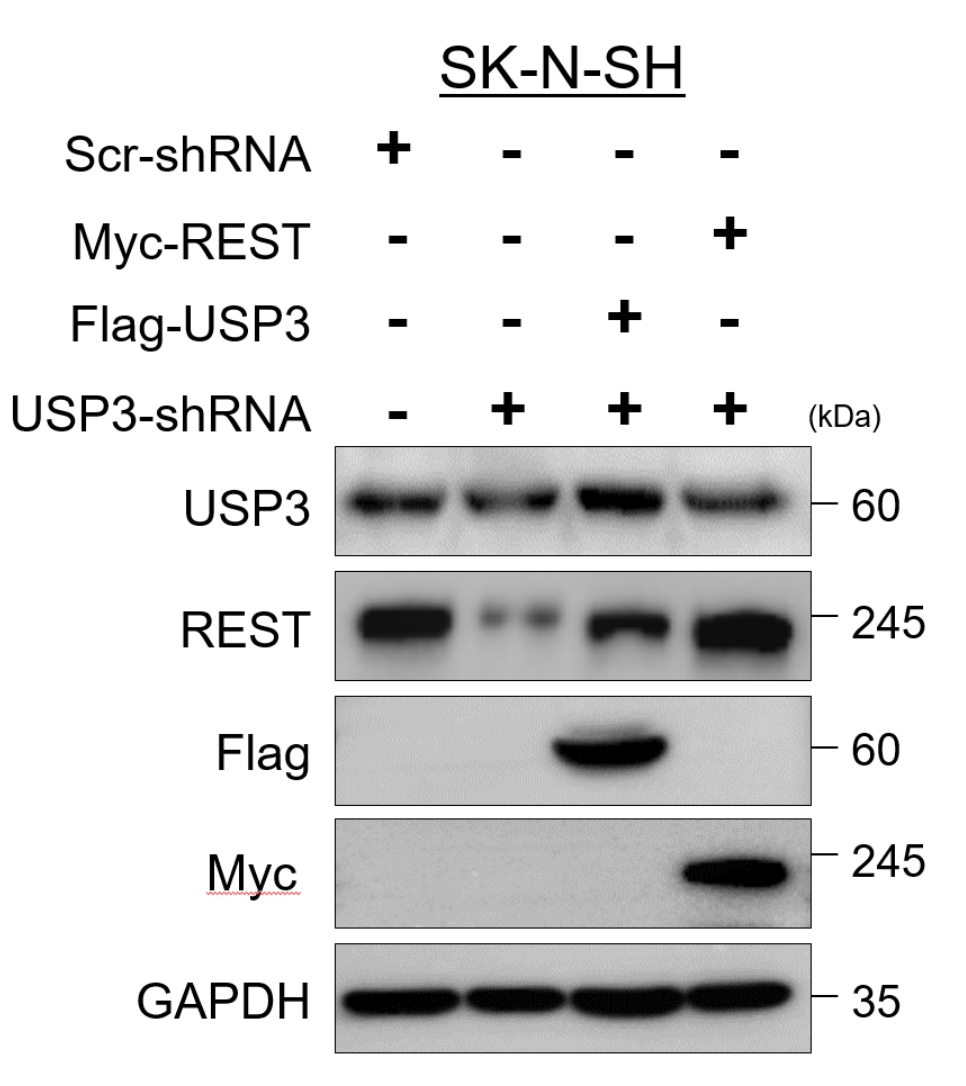


**Supplementary Fig. 12** Validation of USP3 and REST expression in USP3-silenced SK-N-SH cells reconstituted with either Flag-USP3 or Myc-REST**.** SK-N-SH cells transfected with scr-shRNA, USP3-shRNA and USP3-shRNA reconstituted with either USP3 or REST were subjected to western blot analysis. The endogenous and exogenous expression of USP3 and REST were evaluated using indicated antibodies. GAPDH was used as an internal loading control.


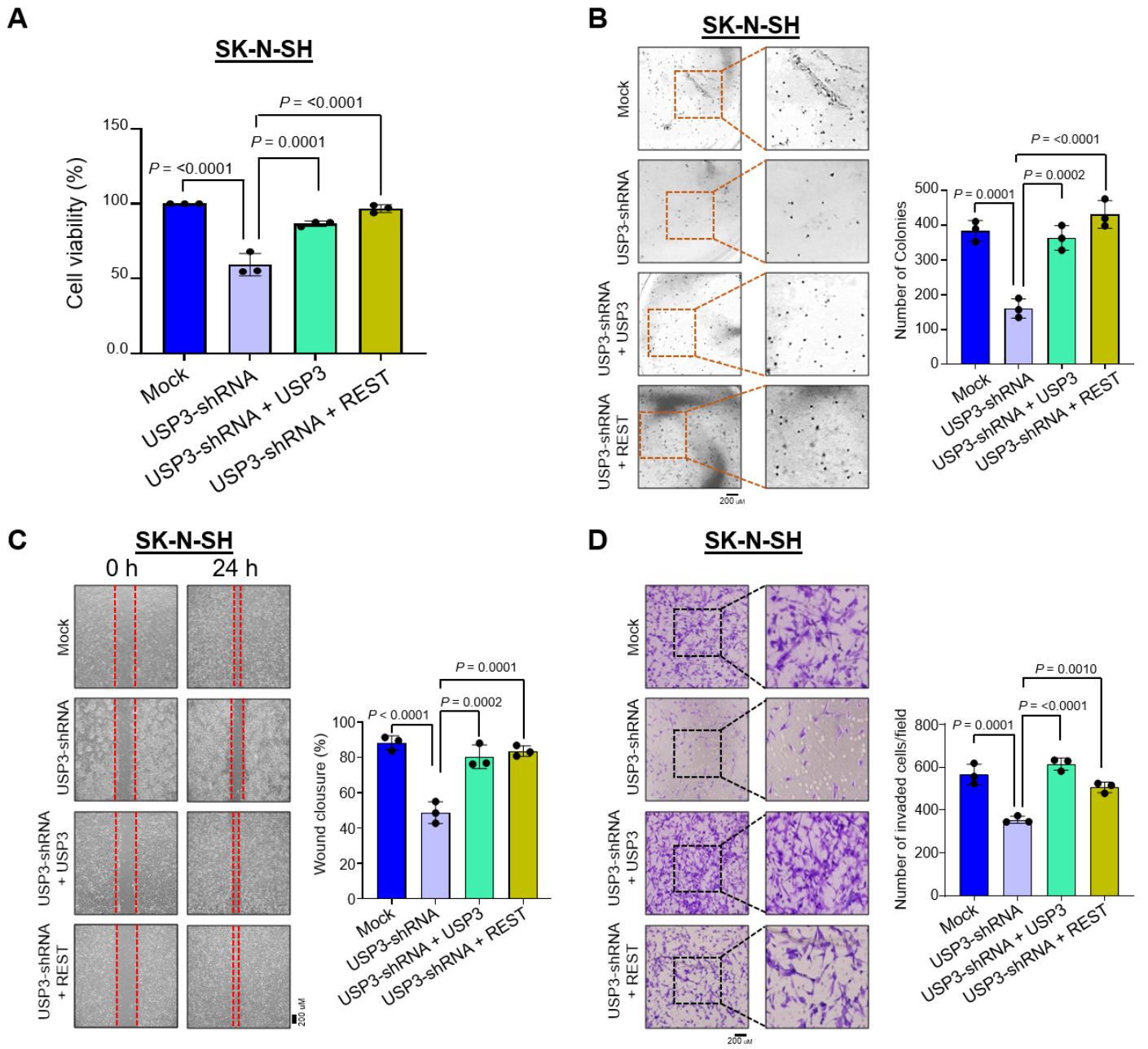


**Supplementary Fig. 13 Loss** of USP3 inhibits cell viability, colony formation, cell invasion and cell migration in SK-N-SH neuroblastoma cells. SK-N-SH cells transfected with scr-shRNA, USP3-shRNA, and USP3-shRNA transfected cells reconstituted with either USP3 or REST were used to perform the following experiments: **(A)** cell viability, **(B)** colony formation, **(C)** cell migration and **(D)** cell invasion. Data are presented as the mean and standard deviation of three independent experiments (n= 3). One-way ANOVA followed by Tukey’s post hoc test was used, and *P* values are indicated.


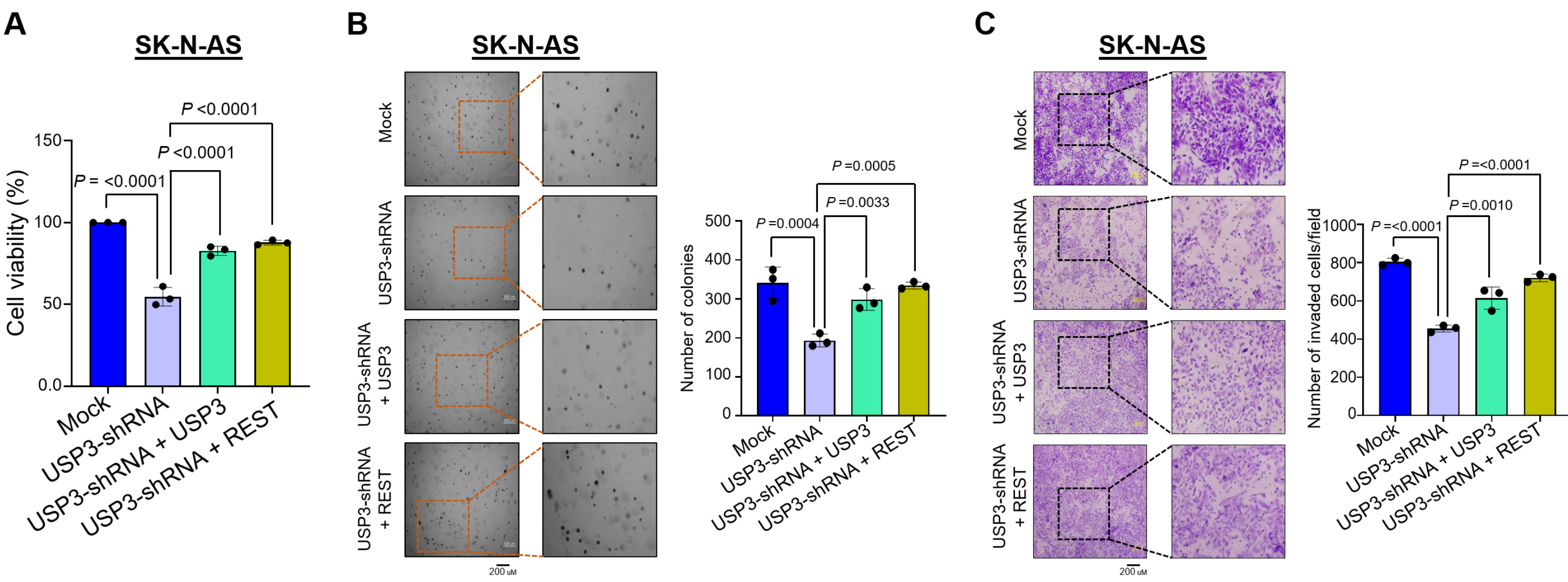


**Supplementary Fig. 14** Loss of USP3 inhibits cell viability, colony formation, and cell invasion in SK-N-AS neuroblastoma cells. SK-N-AS cells transfected with scr-shRNA, USP3-shRNA, and USP3-shRNA transfected cells reconstituted with either USP3 or REST were used to perform the following experiments: **(A)** cell viability, **(B)** colony formation, and **(C)** cell invasion. Data are presented as the mean and standard deviation of three independent experiments (n= 3). One-way ANOVA followed by Tukey’s post hoc test was used, and *P* values are indicated.


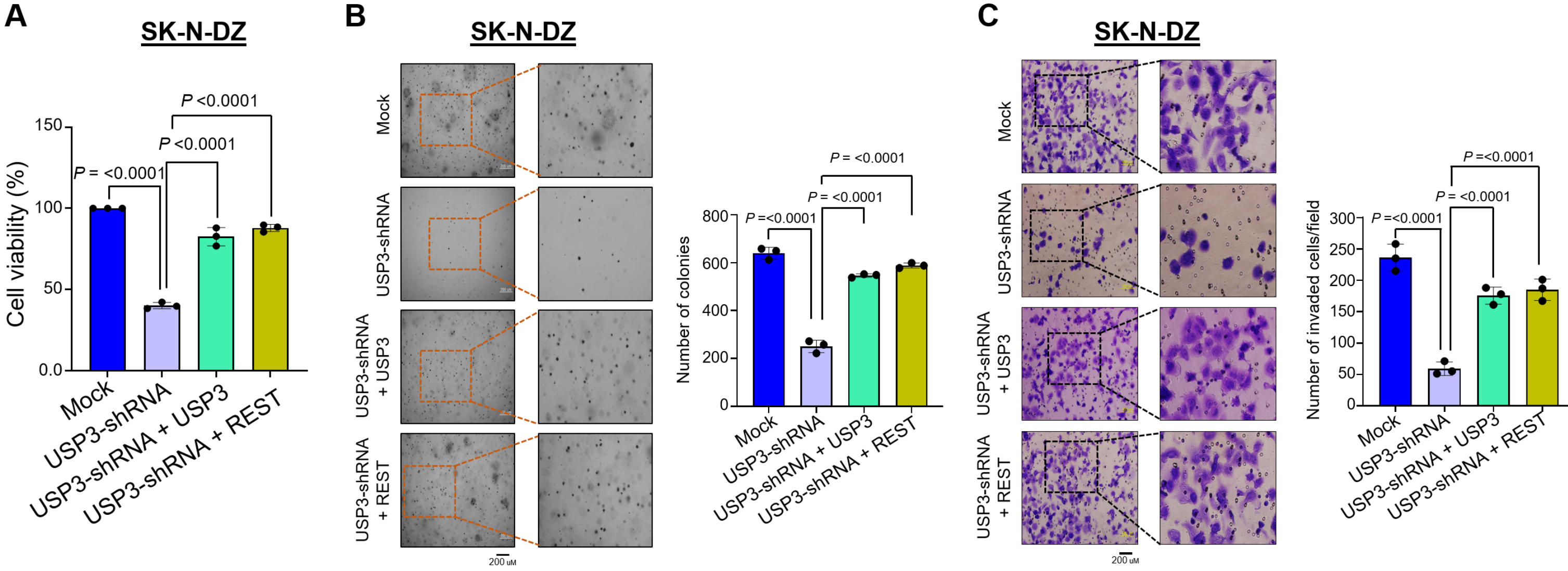


**Supplementary Fig. 15** Loss of USP3 inhibits cell viability, colony formation, and cell invasion in SK-N-DZ neuroblastoma cells. SK-N-DZ cells transfected with scr-shRNA, USP3-shRNA, and USP3-shRNA transfected cells reconstituted with either USP3 or REST were used to perform the following experiments: **(A)** cell viability, **(B)** colony formation, and **(C)** cell invasion. Data are presented as the mean and standard deviation of three independent experiments (n= 3). One-way ANOVA followed by Tukey’s post hoc test was used, and *P* values are indicated.

**Supplementary Table S1. Target sequences used for sgRNA plasmid construction.**

| **Gene** | **sgRNA** | **Direction** | **Sequence (5’ to 3’)** | **Orientation** |
| --- | --- | --- | --- | --- |
| ***USP3*** | sgRNA1 | FP | GACTCAGCCAAGTTCCCCAA | Sense |
|  |  | RP | TTGGGGAACTTGGCTGAGTC |  |
|  | sgRNA2 | FP | CTTTTCTGATTTCTTATGGT | Anti-Sense |
|  |  | RP | ACCATAAGAAATCAGAAAAG |  |
|  | sgRNA3 | FP | CGTCGTCCTGGTGCTGCAGC | Anti-Sense |
|  |  | RP | GCTGCAGCACCAGGACGACG |  |
|  | sgRNA4 | FP | AGTTCAGCACACAGTATGTA | Sense |
|  |  | RP | TACATACTGTGTGCTGAACT |  |

**Supplementary Table S2. Oligonucleotide sequences used to get PCR amplicon for T7E1 assay.**

| **Gene** | **sgRNA** |  | **Direction** | **Sequence (5’ to 3’)** |
| --- | --- | --- | --- | --- |
| ***USP3*** | sgRNA1 and sgRNA 3 | I PCR | FP | TCGGAGTTACACGTTCTACGG |
|  |  |  | RP | CTGCGGAGAAGCGCGG |
|  |  | II PCR | FP1 | TCGGAGTTACACGTTCTACGG |
|  |  |  | RP1 | GCCTCGGGAAACAAAGGA |
| ***USP3*** | sgRNA2 and sgRNA 4 | I PCR | FP | CTTGCCTGAGCCTACTCTTGT |
|  |  |  | RP | AGGATGGATGAGGAACGGGA |
|  |  | II PCR | FP1 | TCACTACATGATTGACTGCTGTT |
|  |  |  | RP1 | AGGATGGATGAGGAACGGGA |

**Supplementary Table S3. PCR amplicon and cleavage sizes after T7E1 assay.**

| **Gene** | **sgRNA** | **PCR size** | **Cleavage size** |
| --- | --- | --- | --- |
| ***USP3*** | sgRNA 1 | 507 | 280+227 |
|  | sgRNA 2 | 504 | 274+230 |
|  | sgRNA 3 | 507 | 311+196 |
|  | sgRNA 4 | 504 | 299+205 |

**Supplementary Table S4. Oligonucleotides sequence used for qRT-PCR.**

| **Gene** | **Direction** | **Sequence (5’ to 3’)** |
| --- | --- | --- |
| *USP3* | FP | CCCAAGGTGCTATGCTTACAT |
|  | RP | AATGTCCAGAACCAACCCCG |
| *REST* | FP | GAGCCTACCTGGTCTTGCTG |
|  | RP | GAAGGGGGAAGCATTGGTGA |
| *TUJ1* | FP | CCTGGAACCCGGAACCAT |
|  | RP | AGGCCTGAAGAGATGTCCAAG |
| *NESTIN* | FP | GTCCGATGGGTTTGCAGATGA |
|  | RP | GGGACACTGACACTCACAGAAT |
| *GAPDH* | FP | GTCATCCCTGAGCTGAACGG |
|  | RP | CCACCTGGTGCTCAGTGTAG |

**Supplementary Table S5. PRECOG meta Z-score for USP3 expression across different cancers.**

| **Cancer subtype** | **Meta Z-score** |
| --- | --- |
| **Brain cancer Neuroblastoma** | **5.95** |
| Germ cell tumors | 3.1 |
| Sarcoma Ewing sarcoma | 1.63 |
| Adrenocortical cancer | 1.48 |
| Hematopoietic cancer DLBCL | 1.35 |
| Head and neck cancer Oral SCC | 1.24 |
| Pancreatic cancer | 1.23 |
| Osteosarcoma | 0.79 |
| Colon cancer | 0.78 |
| Breast cancer | 0.72 |
| Lung cancer LCC | 0.71 |
| Head and neck cancer Oesophageal cancer | 0.36 |
| Lung cancer SCC | 0.33 |
| Lung cancer ADENO | 0.15 |
| Brain cancer Meningioma | 0.12 |
| Brain cancer Medulloblastoma | 0 |
| Head and neck cancer | 0 |
| Head and neck cancer Hypopharyngeal cancer | 0 |
| Mesothelioma | 0 |
| Liver cancer | -0.01 |
| Brain cancer Astrocytoma | -0.08 |
| Melanoma | -0.11 |
| Hematopoietic cancer FL | -0.25 |
| Brain cancer Glioma | -0.31 |
| Gastric cancer | -0.5 |
| Prostate cancer | -0.63 |
| Hematopoietic cancer Burkitt lymphoma | -0.65 |
| Lung cancer SCLC | -0.69 |
| Ovarian cancer | -0.69 |
| Hematopoietic cancer Multiple myeloma | -0.75 |
| Hematopoietic cancer CLL | -0.83 |
| Kidney cancer | -1.09 |
| Hematopoietic cancer AML | -1.76 |
| Hematopoietic cancer B ALL | -1.81 |
| Bladder cancer | -1.88 |
| Melanoma Metastasis | -2.16 |
| Brain cancer Glioblastoma | -2.87 |

**Supplementary Table S6. PRECOG meta Z-score for all the DUBs related to USP subfamily in neuroblastoma.**

| **Gene symbol** | **meta Z-score** |  | **Gene symbol** | **meta Z-score** |
| --- | --- | --- | --- | --- |
| USP1 | 4.52 |  | USP29 | -0.33 |
| USP2 | 0.15 |  | USP30 | -0.71 |
| **USP3** | **5.95** |  | USP31 | 4.47 |
| USP4 | -5.07 |  | USP32 | 1.28 |
| USP5 | 0.62 |  | USP33 | -8.72 |
| USP6 | -0.35 |  | USP34 | -0.43 |
| USP7 | 4.23 |  | USP35 | -7.83 |
| USP8 | -5.41 |  | USP36 | -0.5 |
| USP9X | 4.54 |  | USP37 | 0.22 |
| USP9Y | -6.39 |  | USP38 | 2.34 |
| USP10 | 6.09 |  | USP39 | 5.69 |
| USP11 | 0.92 |  | USP40 | 0.66 |
| USP12 | -1.79 |  | USP41 | 0.22 |
| USP13 | 4.1 |  | USP42 | -1.47 |
| USP14 | 2.75 |  | USP43 | 0 |
| USP15 | -1.72 |  | USP44 | 8.33 |
| USP16 | 2.82 |  | USP45 | -2.42 |
| USP17 | 0 |  | USP46 | 4.54 |
| USP18 | 0.76 |  | USP47 | -1.51 |
| USP19 | 1.86 |  | USP48 | -5.37 |
| USP20 | -3.7 |  | USP49 | -0.88 |
| USP21 | 4.42 |  | USP50 | 0 |
| USP22 | -4.8 |  | USP51 | -1.37 |
| USP24 | -10.14 |  | USP53 | -1.49 |
| USP25 | -1.58 |  | USP54 | 2.48 |
| USP26 | -3.05 |  | USPL1 | -0.57 |
| USP27X | -1.24 |  | CYLD | -3.81 |
| USP28 | -1.57 |  |  |  |

**Supplementary Table S7. The mRNA scores for *USP3* and *REST* expression derived from the Cancer Cell Line Encyclopedia database in neuroblastoma cell lines.**

| **Cell Line Name** | ***USP3* log2(TPM+1)** | ***REST* log2(TPM+1)** |
| --- | --- | --- |
| SKNAS | 4.030336 | 3.581351 |
| COGN305 | 4.123501 | 1.847997 |
| IMR32 | 5.06264 | 2.427606 |
| NH6 | 4.861459 | 3.723559 |
| CHP126 | 4.715344 | 2.454176 |
| TN2 | 3.375735 | 0.111031 |
| COGN278 | 4.432291 | 3.872829 |
| KPNSI9S | 4.071248 | 2.85599 |
| SKNSH | 3.788686 | 3.659925 |
| KPNYN | 4.831877 | 1.327687 |
| NB1643 | 4.558268 | 1.608809 |
| CHLA15 | 3.835924 | 1.469886 |
| SKNFI | 4.646163 | 1.321928 |
| CHP212 | 4.792855 | 4.080658 |
| NGP | 4.801159 | 2.179511 |
| LAN2 | 4.769772 | 1.367371 |
| SHSY5Y | 4.344828 | 2.634593 |
| NB1 | 4.882154 | 3.415488 |
| NH12 | 4.560715 | 0.799087 |
| LS | 4.537917 | 4.020591 |
| SKNDZ | 4.565597 | 3.187451 |
| KPNRTBM1 | 4.457463 | 3.0268 |
| GIMEN | 3.734439 | 3.377124 |
| KELLY | 4.280214 | 1.195348 |
| GOTO | 4.706531 | 3.82171 |
| SKNBE2 | 5.023699 | 3.035624 |
| TGW | 4.9855 | 4.070389 |
| NMB | 4.776104 | 1.111031 |
