# Supplementary material for: CRISPR/Cas9-based genome-wide screening of the deubiquitinase subfamily identifies USP3 as a protein stabilizer of REST blocking neuronal differentiation and promotes neuroblastoma tumorigenesis
Source: J Exp Clin Cancer Res. 2023 May 12;42:121. doi: 10.1186/s13046-023-02694-1 (PMC10176696; doi:10.1186/s13046-023-02694-1)
Supplement: Supplementary file 2 — Supplementary Material 2 [file 13046_2023_2694_MOESM2_ESM.docx]

April 27^th^, 2023

Editor-in-Chief

Journal of Experimental & Clinical Cancer Research

Dear Dr. Mauro Castelli,

Thank you very much for your comments and suggestions regarding publication of our manuscript, “**CRISPR/Cas9-based genome-wide screening of the deubiquitinase subfamily identifies USP3 as a protein stabilizer of REST blocking neuronal differentiation and promotes neuroblastoma tumorigenesis*”*** (Manuscript ID: JECC-D-23-00003) in the Journal of Experimental & Clinical Cancer Research as an original research article.

We are grateful to the editorial board members and the reviewers for their valuable time and useful comments. We are glad that the reviewers found our research to be of interest and importance. Their comments and suggestions have significantly improved the manuscript.

We addressed the concerns they raised in our revision. The two reviewers were positive and gave some helpful and valuable suggestions. Please find our detailed point-by-point response for each comment below. The revised manuscript, in which the changes have been highlighted in blue, is attached. The manuscript has been edited by a professional English editing service.

We trust that our revised version of this manuscript will be of general interest to the readers of the Journal of Experimental & Clinical Cancer Research*.* We appreciate your consideration of our revised manuscript for publication and hope that you find our revised manuscript acceptable. Thank you for your time and attention.

Sincerely,

Suresh Ramakrishna, Ph.D.

**Comments:**
**Reviewer #1:**

In their study, Karapurkar and colleagues explore factors for the regulation of REST, an important transcriptional repressor for self-renewal and differentiation in neuroblastoma. They used a CRISPR knockout screen and identified USP3 as a regulator of REST that interacts with it directly. Mechanistically, they showed that USP3 stabilized REST in a dose dependent manner and dependent on the proteasome. The mechanism shown is convincing, and this study is interesting to neuroblastoma researchers and people interested in protein stability regulation. My comments aim to improve the study to make it suitable for publication.

**Author’s response:** Thank you very much for your positive response to our research study. We have done our best to answer all the comments and performed several additional experiments to improve our revised manuscript.

**Major concerns:**

***Comment 1:*** *Fig 1) The KO screen is an elegant way to screen for a regulation of REST in neuroblastoma cells. However, why was the screen performed in HEK293 cells in the first place? It should have been rather performed in one adrenergic (ADR) and one mesenchymal (MES) neuroblastoma cell line (see Boevia, 2017, van Groningen, 2017) since REST is important for differentiation. Like this, the authors could not only quantify REST levels as an effect of the individual DUB KO, but e.g. also directly observe phenotypic changes.*

**Author’s response:** Thank you very much for your comments and suggestions.

**Explanation for using HEK293 cells for screening DUBs for REST:**

Transient transfection of sgRNA targeting DUBs often causes partial repression or restoration of gene function and is temporary, whereas DUB genes knockout introduces a complete loss-of-function mutation into genomic DNA, and its effect is permanent. Therefore, we generated 50 individual HEK293 knockout cell lines targeting the USP subfamily member genes. This process took around two years to accomplish as a screening kit and was published in *Cell Death and Differentiation, 2020* (PMID: 32415280). The cell lysates from these single cell-derived USP knockout cell lines were used to screen the DUB candidates that regulate the target protein levels using western blot analysis.

For the initial screening process, we relied on the DUB library screening system in normal HEK293 cells to identify putative DUB candidates for the REST protein. Once we obtained the specific DUBs candidates, we then cross-confirmed the interaction, protein stabilization, ubiquitination, deubiquitination, and differentiation in neuroblastoma cells and its phenotypical changes in neuroblastoma. However, based on the aim of the different projects, generating 50 single cell-derived USP knockout cell lines in different cell lines each time would be a laborious task. Thus, our HEK293 DUB KO screening kit can be applied to the initial screening of DUBs for various targets and to later elucidate its molecular functions in the relevant cell lines specific to cancer biology, neurobiology, and stem cell biology.

We have performed a similar initial screening for several target proteins using HEK293 DUB knockout cell lines and further investigated its functional analysis on the relevant cell lines, which are listed below:

- USP3 regulating CDC25A: Screened in HEK293 cells and performed functional assays in HeLa cells (*Cell Death and Differentiation, 2020* (PMID: 32415280) and was cited in several articles (Citation 24).
- USP49 regulating MSX1 and PAX9: Screened in HEK293 cells and performed functional assays in human dental pulp stem cells (*Cell Death and Differentiation, 2022* (PMID: 35273362)).
- USP32 regulating SLC35F2: Screened in HEK293 cells and performed functional assays in breast cancer cells (*Theranostics*, 2021 (PMID: 34815782)).
- USP19 regulating Survivin: Screened in HEK293 cells and performed functional assays in HCT116 colon cancer cells (Molecular Therapy, 2022 (PMID: 35918893)).
- USP1 regulating MAST1: Screened in HEK293 cells and performed functional assays in A549 lung cancer cells (Theranostics, 2022 (PMID: 35966591)).

***Comment 2:*** *In line with this, it seems that the KO efficiency is not as high in SH-SY5Y cells as in HEK293 cells. Here, the KO led only to a 50% reduction in USP3 and a smaller effect on REST.*

**Author’s response:** Thank you very much for your comments. In Figure 1C, the knockout efficiency of USP3 in HEK293 is high because the results were obtained from the **single cell-derived** USP3 gene knockout in the HEK293 cell lines obtained from the DUB KO library (previously published PMID: 32415280). Whereas in Figure 1D, the knockout efficiency of USP3 in SH-SY5Y is low because the results were obtained from the transient transfection of sgRNA targeting USP3 in SH-SY5Y cells.

Since Figure 1 represents the initial screening results of DUBs for REST protein, we cross confirmed the effect of USP3 knockout on REST protein stability in SH-SY5Y by transient transfection (Fig 1D). Later for the functional analysis, we generated **single cell-derived** USP3 gene knockout cell lines and performed the experiments. For efficiency of USP3 KO in SH-SY5Y please refer to Figure 7D. Generation of single cell-derived USP3 gene knockout cell lines will take at least one month. The details of the generating USP3 KO SH-SY5Y were mentioned in the materials and methods section under the sub heading “Generation of a USP3-knockout SH-SY5Y cell line using CRISPR/Cas9, page no 11” and in the results section under the sub heading “Generation of single cell-derived USP3-knockout clones in human neuroblastoma cell lines, page no. 29.”

***Comment 3:*** *Fig 2) The used CRISPR Cas9 approach to deplete USP3 is not very efficient. The authors should try this not only on a transient level and maybe employ further sgRNAs.-*

**Author response:** Thank you very much for this analysis. The reason and necessary changes are listed below.

- In this study, we implemented both single guide RNA (sgRNAs) (Fig. 2G) and short hairpin RNA (shRNA) (Fig. 2I) to deplete USP3 and performed all the biochemical and functional assays.
- We have **transiently** transfected sgRNAs targeting USP3 in HEK293 cells. We noticed that the efficiency of sgRNA1 targeting USP3 is more than 80% and sgRNA2 targeting USP3 is around 50% (Figure 2G, USP3/GAPDH ratio is mentioned below the blot).
- However, as suggested by the reviewer, we designed two more additional sgRNAs to target USP3 and validated their knockout efficiencies. The result showed that sgRNA1 has high cleavage efficiency when compared to all other sgRNAs targeting USP3 (refer to the revised Figure 2F-H). Thereafter, we used the best sgRNA1 targeting USP3 for further analyses and generated single cell-derived USP3 knockout SH-SY5Y cells showing complete aberration of USP3 expression (Figure 7D).
- Additionally, we have also implemented shRNA-based silencing of USP3 as an additional knockdown system and compared its downregulation of USP3 protein and subsequent effect on REST protein expression (Fig. 2I).

***Comment 4:*** *Fig. 5 and further) The authors perform all their functional assays only with 2 non-MYCN-amplified (MNA) cell lines. At least one MNA cell line should be included. SY5Y are ADR cells, while SKNSH is intermediate (Boeva, 2017). Since the authors investigate differentiation, at least one MES cell line should be included, such as SHEP. How are ADR and MES markers changing after USP3 knockdown?*

**Author response:** Thank you very much for the comments and suggestions. As suggested by the reviewer, we have now collected at least one neuroblastoma cell line that is classified under MYCN-amplified (MNA), MYCN-nonamplified, ADR and MES cell lines. In this study, we used the following neuroblastoma cell lines (details of the cell lines are mentioned in the materials and methods section under sub heading “cell culture and transfection”, page no. 9)

SK-N-DZ: MYCN-amplified/ADR

SK-N-AS: MYCN-nonamplified/MES

SH-SY5Y: MYCN-nonamplified/ADR

SK-N-SH: MYCN-nonamplified/intermediate

Using these cell lines, we performed several functional assays which are listed below:

- Neurosphere formation assay,
- Neurosphere limiting dilution assay,
- Cell proliferation assay,
- Colony formation assay,
- Cell migration assay,
- Cell invasion assay,
- Cell viability assay,
- RA differentiation,
- Immunostaining

All these functional assays are represented in Figure 5 and supplementary Figure 5-15:

Additionally, we have also checked the effect of USP3 knockdown on the ADR and MES markers in these cell lines (Please refer to the revised Supplementary Figure 5A, for text refer to page no. 26).

***Comment 5:*** *Fig. 6) The authors demonstrate by various techniques that USP3 stabilizes REST. The hypothesis is therefore that high USP3 and REST levels should be associated with an undifferentiated phenotype. However, when analyzing published datasets in Fig. 6B) the authors found no difference of REST expression in USP3 high and low samples. How do the authors explain this? Are there more regulatory factors involved? What is the expression of REST in the 2 groups analyzed in 6A, differentiated and non-differentiated? The authors could also explore published mass spec proteomics data from Hartlieb et al. 2021 to see whether there is a difference on the protein level. Further, the numerical changes in differentiation markers in Supp Fig 6B/C are small and should not be overstated.*

**Author response:** We sincerely agree with the reviewer’s suggestion that Fig. 6B data might mislead the reader. Therefore, we have removed Fig. 6B and C and Supplementary Fig. 3A–C from our revised manuscript.

**Explanation of Fig 6 B and C:**

Fig. 6 B shows the mRNA expression of REST in the neuroblastoma cell lines expressing high or low USP3 mRNA using the bioinformatics database. The purpose of this data is to show how the REST gene is expressed in neuroblastoma cells irrespective of the regulatory role of USP3 on the REST protein level. Our intention was just to report the mRNA expression behavior of REST in neuroblastoma cells with different levels of USP3 mRNA expression. Since these results might confuse the reader, we have now removed this data (previous Fig. 6B) including the supplementary data showing the expression of the differentiation markers (previous Sup. Fig. 3A–C) from our revised manuscript.

Additionally, as the reviewer suggested, we have included the REST mRNA expression in the neuroblastoma cell lines from the differentiated or undifferentiated group using the bioinformatics database (please refer to the revised Fig. 6B)

Regarding including the mass spec proteomics data from Hartlieb et al. to compare the protein level of USP3 and REST, no data related to the REST protein from this dataset was available. Therefore, we were not able to perform this analysis. I sincerely request the reviewer to consider our explanation.

***Comment 6:*** *Fig. 6F) While the differentiated phenotype for SK-N-SH cells is convincing, the morphological changes for SY5Y are less promoted. It seems that the control cells show the same cellular outgrowth, but are grown to a higher density, which makes it less visible. Seeding at identical densities (probably with adjusting to the fact that USP3 knockdown cells proliferate less, as shown in Fig. 5F) and a phalloidin immunofluorescence staining could help to make the cellular outgrowth more visible.*

**Author’s response:** Thank you very much for your suggestions. We have now repeated this experiment by seeding identical densities and considering how USP3 knockdown affects cell proliferation. Differentiation was induced in SH-SY5Y and SK-N-DZ cells due to the knockdown effect of USP3 (please refer to the revised Fig. 6E and Supplementary Figure 10A). Additionally, as the reviewer suggested, we have stained RA differentiated SH-SY5Y and SK-N-DZ cells from the USP3 knock-down group with phalloidin and represented it in the revised manuscript (please refer to revised Figure 6F and Supplementary Figure 10B).

***Comment 7:*** *It would be interesting to analyze under cancers, where differentiation plays a role, such as acute promyelocytic leukemia that responds as well to retinoic acid, for a potential role of USP3 and REST.*

**Author’s response:** To reflect your comments, we have now included in our discussion section the importance of studying the role of the USP3/REST axis in other cancers that responds to retinoic acid treatment (please refer to discussion section page no. 35).

However, we will seriously consider your suggestion for our follow up research on the role of USP3 and REST in acute promyelocytic leukemia. Our current study only focuses on demonstrating the REST protein stabilization by the deubiquitinating enzyme USP3 and its effect on neuroblastoma differentiation and also emphasizes USP3 as a therapeutic target in neuroblastoma tumors for improved survival rates of neuroblastoma patients. I sincerely request the reviewer to consider our explanation.

**Minor concerns:**

***Comment 8:*** *Fig. 1) The DUB KO screen is interesting. However, I was wondering, how the cell viability was affected after those knockouts. It would be helpful to show this at least in the supplement. Was the largest effect on viability for the USP 3,7 and 19 KO observed in comparison to the KO where no effect on REST was seen?*

**Author’s response:** Thank you very much for your insights.

As suggested by the reviewer, we have now performed an additional cell viability assay in SH-SY-5Y neuroblastoma cells for putative positive DUBs for REST protein and some DUB candidates that do not have any effect on the REST protein level but were included as supplementary data. We observed low cell viability in USP3, 7, and 19 KO with a down-regulating effect on the REST protein level when compared with a few other USPs KO cells where there is no effect on the REST protein level (please refer to the revised Supplementary Fig. 1, revised text in page no. 20).

***Comment 9:*** *Fig 3G) The CHX experiment should be combined with the USP3 KD/KO. Does this lead to a quicker degradation of REST?*

**Author’s response:** Thank you very much for your question. We have now performed additional CHX experiments using USP3 knockdown cells and USP3 knockdown cells reconstituted with Flag-USP3 or Flag-USP3CS. The REST protein level was rapidly degraded in the USP3 knockdown cells when compared with the wild type USP3 cells. However, the reconstitution of Flag-USP3 in USP3 knockdown cells regained the protein level of REST (please refer to revised Fig. 3G and 3H). Previously, original figure 3G representing the effect of overexpressing USP3 or USP3CS on REST protein half-life was moved to supplementary figure 2.

***Comment 10:*** *Fig. 4A) CCLE data are interesting, which NB cell lines are analyzed? The authors should probably test a panel of own cell lines and differ between MYCN amplified vs. non-amplified and ADR vs MES cell lines, since REST is important for differentiation. o       How is REST expressed?*

*o        How is the expression of REST/USP3 related to MYCN in tumor samples?*

**Author’s response:** Thank you very much for your suggestions. We have given detailed information about the cell lines obtained from the CCLE database (Fig. 4A) in supplementary Table S7.

As suggested by the reviewer, we have used the following neuroblastoma cell lines to check the expression of REST and USP3 in the revised manuscript (please refer to the revised Supplementary Fig. 5B, please refer to the text in page no. 26).

SK-N-DZ: MYCN-amplified/ ADR

SK-N-AS: MYCN-nonamplified/ MES

SH-SY5Y: MYCN-nonamplified/ ADR

SK-N-SH: MYCN-nonamplified/ intermediate

***Comment 11:*** *Fig 4C) What is the most highly upregulated USP, was it also found in the CRISPR KO screen?*

**Author’s response:** Thank you very much for your question. The most highly upregulated DUB was USP44 (which was not detected in our KO screening). So far there is no published report on the role of USP44 in neuroblastoma. However, a computational analysis by Paul Galardy from the Mayo clinic predicts that USP44 may play a critical role in patients with neuroblastoma (<https://www.technologynetworks.com/cancer-research/news/researchers-identify-gene-implicated-in-neuroblastoma-345726>) (PMID: 33355202). We have now included this information in our discussion section (please refer to page no. 35).

However, from our CRISPR KO screen, we did not find that USP44 regulates REST protein stability. USP44 might regulate other protein substrates, however, and its role in neuroblastoma is yet to be investigated.

***Comment 12:*** Fig 4F) REST is not expressed in all NB cell lines. Do the cell lines that express it have a common genomic profile, e.g. MYCN amp., p53 mut, ADR/MES, ALK mut ?

**Author’s response:** Thank you very much for your comment. Here, we are focusing this research to study the deubiquitinating function of USP3 on REST protein stabilization and its implications for neuroblastoma differentiation.

We sincerely agree with the reviewer’s suggestion. However, in order to check the common genomic profile, we need to perform experiments on several neuroblastoma cells from different categories such as MYCN-amplified (MNA), MYCN-nonamplified, ADR, and MES cell lines. At this point, performing genomic profiling is beyond the scope of our research goal. However, we will consider this suggestion in the future as follow up research. I sincerely request the reviewer to consider our explanation. However, we checked the expression of REST and USP3 in the SK-N-DZ, SK-N-AS, SH-SY5Y, and SK-N-SH neuroblastoma cell lines and updated this in the revised manuscript (please refer to the revised Supplementary Fig. 5B).

***Comment 13:*** Fig 4G, Supp Fig 2b) The correlation of USP3 and REST is very weak. The authors could try to do a subgroup analysis and check only MNA or non-MNA samples, or MES/ADR tumors?

**Author’s response:** Thank you very much for your suggestions. As suggested by the reviewer, we performed a correlation analysis between USP3 and REST in the MNA and non-MNA subgroups. However, the correlation coefficient between USP3 and REST was 0.36 and 0.39 in the MNA and non-MNA subgroups, respectively. The result is similar to the correlation coefficient between USP3 and REST (r = 0.32) from the combined neuroblastoma cell lines. Therefore, we wished to retain the results of Fig. 4G to maintain the uniformity in USP3/REST in the silico analysis throughout the manuscript. Additionally, we removed “positive” correlation word from the subheading. (please refer to result section subheading “A correlation between USP3 and REST in neuroblastoma”, page no. 25). We are providing the analyzed data for the reviewers only in the figure below:

***Comment 14:*** *Fig 5a) Knockdown of USP3 downregulates REST, which should promote differentiation. This is indicated by downregulating the marker of neuronal precursors Nestin and inducing beta 3 tubulin as a neuronal marker. However, further neuronal markers should be assessed, such as NEFL, TrkA and Map2. This is as well the case for Fig. 6B (actually 6D), the RA treatment.*

**Author’s response:** Thank you very much for your suggestions. We included the other neuronal markers during neuroblastoma differentiation (please refer to the revised Fig. 5A and 6C and Supplementary Fig. 6 and 8A).

***Comment 15:*** *Fig. 5b) The graphical representation of this plot is a bit hard to interpret, it should be changed so that it is easier to understand for the reader, e.g. by a box plot for the different numbers per well.*

**Author’s response:** Thank you very much for your suggestion. We used the standard bioinformatics software called Extreme Limiting Dilution Analysis (ELDA). ELDA is suitable for any limiting dilution assay result analysis that gives reliable results. We followed published articles in the field that have used the same software as the standard method and represented such graphs in a similar manner. (Examples of published articles are listed below).

- CircLRFN5 and glioblastoma progression (*Journal of Experimental & Clinical Cancer Research*, 2022 (PMID: 36266731)).
- UPF1/circRPPH/ATF3 and stemness of GSCs (Cell Death and Disease, 2022 (PMID: 35871061)).
- USP16 and stem cell aging and memory in an Alzheimer’s model (Elife, 2022 (PMID: 35311644))
- LGR5 and tumorigenicity and invasion of glioblastoma (The Journal of Pathology, 2019 (PMID: 30357839)).
- Assessment self-renewal in adult mouse neural stem cells by ELDA (Methods in molecular biology, 2022 (PMID: 35776362)).
- USP1 and targeting GBM growth by inhibiting stem cell maintenance (Neuro-oncology, 2016 (PMID: 26032834)).

***Comment 16:*** *Fig. 7) Neuroblastoma is not an epithelial tumor. Terms like EMT should not be used in this context and are misleading. I suggest removing assays concerning this, since it might confuse the reader.*

**Author’s response:** Thank you very much for your suggestions. We have now removed the context of EMT from our revised manuscript (deleted text was in page no. 31 in previous original manuscript).

***Comment 17:*** *There are already USP30 inhibitors commercially available. Do the authors envision the development of an USP3 inhibitor, as a treatment strategy? A drug screening would be an interesting follow-up study.*

**Author’s response:** Thank you very much for your idea. As the reviewer noticed, synthesizing a USP3 inhibitor would be an important potential therapeutic agent. We have already collaborated with a pharmaceutical company to synthesize a specific USP3 inhibitor. The work is in progress and will be updated as a research article in the future.

***Comment 18:*** *English language should be critically revised. Phrases like "Since acquisition of EMT phenotype plays a critical role in tumor cell invasion and migration" are for example hard to read.*

**Author’s response:** Thank you very much for your suggestion. We have corrected all the grammatical errors in the revised manuscript using an English proofreading service. The English edited certificate is attached as a supplementary file.

***Reviewer #2:***

*The authors have used a CRISPR/Cas9-based genome-wide screening of deubiquitinase subfamily to identify USP3 as a protein stabilizer of REST. They also claim that stabilization of REST results in block of neuronal differentiation and promotes neuroblastoma tumorigenesis.*

*More In detail they suggest USP3 as a prognostic marker for overall survival in neuroblastoma patients, and demonstrated that silencing of USP3 led to a decreased self-renewal capacity and promoted retinoic acid-induced differentiation in neuroblastoma cell lines. Furthermore, loss of USP3 led to attenuation of REST-mediated neuroblastoma tumorigenesis in a mouse xenograft model. The authors propose that targeting USP3 in neuroblastoma tumors might provide an effective therapeutic strategy for neuroblastoma patients.
The paper is in general well written and most of the figures present solid data. I believe the role of USP3 on REST has been demonstrated, and also the in vivo data presented are convincing. I believe that, given a few corrections, a revised version of the paper could be accepted for publication.*

**Author’s response:** Thank you very much for your positive response to our research study. We have done our best to answer all the comments and performed several additional experiments to improve our revised manuscript.

***Comment 1:*** *Inparticular: Figure 2, panels A and B: the IP experiments do not show the IgG control but only non transfected and transfected samples. This is not a proper control The correct representation of the data should be as in panel C.*

**Author’s response:** Thank you for pointing out the need for correction. We have now repeated this experiment by including IgG as well as non-transfected cells as control samples. In additional, we loaded both the USP3 and USP19 IP samples in the same gel (please refer to the revised Fig. 2A).

***Comment 2:*** *Figure 2: The size of the pictures in panel D (duolink PLA) is very small and the quality of the pictures does not allow to see the red dots. The TIFF picture that I have downloaded is good. What I think should be done, is to provide an enlargement not only of the USP+ REST expressing cells, but also of the others, to verify no aspecific coloration in the other samples.*

**Author’s response:** Thank you for your suggestion. We have now provided enlarged images of all three of the samples for better visualization (please refer to the revised Fig. 2C).

***Comment 3:*** *I have noticed that in figure 3 panel C, D, E and F, the amount of Immunoprecipitated protein seems relatively lower than I would have expected. Please explain.*

**Author’s response:** We truly agree with the reviewer’s comment.Generally, we take a larger amount of protein samples for immunoprecipitation. Therefore, the immunoprecipitated samples look relatively high in the western blot images.

However, recently we received feedback from several reviewers (at least from 6 to 8 reviewers) commenting on the saturated bands (blob) of the immunoprecipitated samples. They suggested that we load a lesser amount or avoid overexposure of the immunoprecipitated samples. This is the main reason why we loaded less immunoprecipitated samples for Figure 3 C, D, E and F to obtain a sharp band and avoid saturated protein bands (blobs) on the western blot images.

***Comment 4:*** *Figure 4D: I am not sure that normal brain is the best control for neuroblastoma samples. Peripheral nerve tissue could be more indicated.*

**Author’s response:** Thank you very much for your comments. Unfortunately, we did not get peripheral nerve tissue expression values in the Versteeg dataset. Therefore, we retained the same data. I hope this response explains our decision.

***Comment 5:*** *FIG 4 G: In the analysis of REST and USP3 in neuroblastoma samples, the authors highlight that there is a good correlation between the mRNA expression of both genes, and suggest that this is an evidence of the role played by USP3 on REST. This is probably the wrong way to discuss these data, that indeed do not demonstrate much. In fact the correlation of the two mRNA would not prove anything, since USP3 is expected to act at the level of REST protein, and should not have any effect on mRNA levels of REST. Indeed the authors demonstrate this fact in two panels Fig 6B and 7C, in which alteration of USP levels do not affect REST mRNA expression. I understand that is very difficult to have data that allow comparison of mRNA and protein expression from the same tumor samples. But if the authors want to highlight the relevance of the correlation in human tumor samples, they may be able to exploit the several cell lines that they show in figure X panel F, adding a western blot analysis that shows the protein levels of REST in the various line and verifying if the correlation is significant.*

**Author’s response:** Thank you very much for your suggestion. As suggested by the reviewer, we used the following neuroblastoma cell lines to check the expression of REST and USP3 by western blotting in the revised manuscript (please refer to the revised Fig. 5B).

SK-N-DZ: MYCN-amplified/ADR

SK-N-AS: MYCN-nonamplified/MES

SH-SY5Y: MYCN-nonamplified/ADR

SK-N-SH: MYCN-nonamplified/intermediate

Additionally, in the previous Fig. 6B, we showed no difference in the REST expression in the USP3 high and low samples, which we thought might mislead the reader. As suggest by the reviewer (another reviewer also suggested to remove this analysis result), we have removed Fig. 6B and C and Supplementary Fig. 3A–C from our revised manuscript.

**The reason to retain Fig. 4F and 4G in our manuscript:**

We agree with the reviewer’s comment that the mRNA co-relation between USP3 and REST does not prove anything, as USP3 is a DUB regulating REST protein level. As mentioned by the reviewer, we demonstrated that USP3 regulates the REST protein level but not at the mRNA level in Fig. 6B and 7C.

From Fig. 4A to 4E, we confined our investigation to the mRNA expression of USP3 in different neuroblastoma to determine whether USP3 is a prognostic factor in neuroblastoma. Only in Fig. 4F and 4G did we check the expression level of both USP3 and REST at the mRNA level in the neuroblastoma cells. The purpose of this data is to show how these two genes are expressed in neuroblastoma cells irrespective of the regulatory role of USP3 on the REST protein level. Our intention is to just report the mRNA expression behavior of USP3 and REST in different neuroblastoma. We have not misled the readers that the positive correlation between USP3 and REST has a functional role on neuroblastoma differentiation. I hope the reviewer considers our explanation sufficient to retain the mRNA co-relation data in our revised manuscript.

Recently, we have published several research articles showing a similar bioinformatic analysis between DUBs and its target proteins and reported whether it has a positive correlation or negative correlation. Some of the published examples are

- USP32 and SLC35F2 show a negative correlation (*Theranostics*, 2021 (PMID: 34815782)).
- USP1 and MAST1 show a positive correlation (Theranostics, 2022 (PMID: 35966591)).
- USP19 and survivin show a positive correlation (Molecular Therapy, 2022 (PMID: 35918893)).

***Comment 6:*** *In the discussion of Figure 6D I suggest the authors to stress the fact that RA treatment induces a reduction in USP3 levels some time before the reduction of REST, suggesting a role for USP3 in modulating REST levels.*

**Author’s response:** Thank you very much for your suggestion. We have now included the down-regulation of USP3 being much faster than REST during RA treatment in our discussion section (please refer to page no. 35).

**Minor point:**

***Comment 7:*** *panel 6I: is indicated NESTIN, but from the text, line 31, and the figure legend, it is indicated as NES.*

**Author’s response:** Thank you very much for your suggestion. We have now updated NESTIN in our revised manuscript (please refer to page no. 29).
